# Motif types, motif locations and base composition patterns around the RNA polyadenylation site in microorganisms, plants and animals

**DOI:** 10.1186/s12862-014-0162-7

**Published:** 2014-07-23

**Authors:** Xiu-Qing Li, Donglei Du

**Affiliations:** 1 Molecular Genetics Laboratory, Potato Research Centre, Agriculture and Agri-Food Canada, 850 Lincoln Road, Fredericton E3B 4Z7, New Brunswick, Canada; 2 Quantitative Methods Research Group, Faculty of Business Administration, University of New Brunswick, 7 Macaulay Lane, Fredericton E3B 5A3, NB, Canada

**Keywords:** Genome evolution, Conserved sequences, Deep branch, Unicellular eukaryotes, Gene selection, UTR length, Complex pattern evolution, NCBI mRNA database, Illumina RNA-Seq, Potato late blight

## Abstract

**Background:**

The polyadenylation of RNA is critical for gene functioning, but the conserved sequence motifs (often called signal or signature motifs), motif locations and abundances, and base composition patterns around mRNA polyadenylation [poly(A)] sites are still uncharacterized in most species. The evolutionary tendency for poly(A) site selection is still largely unknown.

**Results:**

We analyzed the poly(A) site regions of 31 species or phyla. Different groups of species showed different poly(A) signal motifs: UUACUU at the poly(A) site in the parasite *Trypanosoma cruzi*; UGUAAC (approximately 13 bases upstream of the site) in the alga *Chlamydomonas reinhardtii*; UGUUUG (or UGUUUGUU) at mainly the fourth base downstream of the poly(A) site in the parasite *Blastocystis hominis*; and AAUAAA at approximately 16 bases and approximately 19 bases upstream of the poly(A) site in animals and plants, respectively. Polyadenylation signal motifs are usually several hundred times more abundant around poly(A) sites than in whole genomes. These predominant motifs usually had very specific locations, whether upstream of, at, or downstream of poly(A) sites, depending on the species or phylum. The poly(A) site was usually an adenosine (A) in all analyzed species except for *B. hominis*, and there was weak A predominance in *C. reinhardtii*. Fungi, animals, plants, and the protist *Phytophthora infestans* shared a general base abundance pattern (or base composition pattern) of “U-rich—A-rich—U-rich—Poly(A) site—U-rich regions”, or U-A-U-A-U for short, with some variation for each kingdom or subkingdom.

**Conclusion:**

This study identified the poly(A) signal motifs, motif locations, and base composition patterns around mRNA poly(A) sites in protists, fungi, plants, and animals and provided insight into poly(A) site evolution.

## Background

The polyadenylation of RNA is under very active study at the level of individual or small groups of species, but the evolutionary tendency of RNA polyadenylation as the complexity of life increases is still unclear. Research is required to characterize motif types, locations, and abundances relative to the whole genome, as well as the conserved base composition patterns around polyadenylation [poly(A)] sites in a large number of species of different kingdoms and subkingdoms.

In the upstream region of mRNA poly(A) sites, the hexanucleotide motif AAUAAA (or AATAAA for DNA), which was first characterized in simian virus 40 (SV40) mRNAs, is highly conserved in human (*Homo sapiens*) [1],[2], some other mammals [3],[4], and the yeast *Schizosaccharomyces pombe*[5] and is therefore considered to be the poly(A) signal [6]–[9]. The AAUAAA motif is not detected only in RNAs synthesized by RNA polymerase II; some short interspersed element (SINE) transcripts generated by RNA polymerase III in HeLa cells can also be polyadenylated in an AAUAAA-signal-dependent manner [10]. The AAUAAA motif likely also plays a role in RNA 3′-end *trans*-splicing in the nematode *Caenorhabditis elegans*[11]. Many mRNAs have alternative poly(A) cleavage sites [3],[12],[4],[13],[14], but these alternative sites likely still use A[A/U]UAAA as a poly(A) signal [14]. This motif is recognized by the cleavage and polyadenylation specificity factor [15]. Software packages for poly(A) site prediction are often based mainly on the upstream motif AAUAAA or similar motifs [16]–[18]. Several other less frequent motifs are also found upstream of the poly(A) site, including a UUUGUA motif in human [19], a U-rich UUUUUUAU motif in *Xenopus* oocyte mRNA [20], and a C-rich motif (14 C’s upstream of AAUAAA) in *Xenopus* embryo mRNA [21]. It is proposed that interactions of different components of the plant polyadenylation apparatus with their respective sequence elements and with each other are needed for efficient mRNA 3′-end formation [22]. The total polyadenylation efficiency is likely a function of all related elements [23], but non-AAUAAA motifs in most species are still unknown. Although it is known that the AAUAAA motif is less frequent in plants [24] and yeast [25] than in animals, it is not known whether AAUAAA is still the most conserved or most frequent hexanucleotide motif in the region in plants and other non-model species.

Several motifs (elements) downstream of the poly(A) site were detected in human cells, including a CCUCCC element through an in vivo tandem polyadenylation assay using a plasmid [26], a UUAUUU motif through bioinformatic analysis [19], a UGUGUG element [19],[27], a UGUGUGUU and GUGUGUGUUUG elements [28], and an AUGCGUUCCUCGUCC element [29] through the binding assay using a cleavage stimulation factor (CstF). Since most of these elements were detected by promoting polyadenylation in one plasmid or binding to a specific protein, it is unclear what proportion of poly(A) sites having these downstream elements and how often these elements occur in other species.

In an early study using expressed sequence tag (EST) analysis, an adenosine (A) peak approximately 20 nucleotides upstream of the cleavage site was detected among ESTs in six species; however, only common fruit fly (*Drosophila melanogaster*) ESTs were mapped to the DNA sequences [23]. Some sites may have been duplicated, because the DNA sequences were not assembled into the genome, and the threshold allowing six mismatches per 60-nucleotide EST might have resulted in mapping to places that were not necessarily the genes corresponding to the ESTs. A PolyA-seq study found great variation in base composition in the poly(A) site region in pooled sequence reads of five mammals [4]. Using EST databases, researchers analyzed motifs and base frequencies of poly(A) regions in rice [30] and Arabidopsis (*Arabidopsis thaliana*) [31]; however, the mapped poly(A) sites also included the nontemplate-added nucleotides, and the real poly(A) tail starting position was actually one or a few nucleotides later in comparison with most other poly(A) sites. Even though studies on this mixture of the mapped poly(A) sites can also generate useful information, this approach may lower the heights of base frequency peaks (i.e., base abundance peaks) and change the average locations of base frequency peaks. Although valuable knowledge has been gained from analyzing poly(A) sites in individual species, at least a few species per subkingdom are needed in order to have a relatively general idea about that subkingdom. Further research is required to identify the precise location of each component of base frequency peaks in the poly(A) site region, to identify motif differences between species, and to evaluate motif abundance differences between the poly(A) site region and the whole genome. Research using nonredundant poly(A) sites may minimize the bias caused by developmental or environmental differences in transcript abundance and therefore may facilitate the comparison of different kingdoms or subkingdoms.

We previously studied the nucleotide frequencies at the “poly(A) tail attachment position” [i.e., the RNA nucleotide directly in attachment to the poly(A) tail] and the “poly(A) tail starting position” [i.e., the genomic or pre-mRNA nucleotide corresponding to the first A on the poly(A) tail] [32]. We found that all the 29 most mapped species (2 fungi, 2 protists, 18 animals, and 7 plants) typically used an A at the poly(A) tail starting position [i.e., A-type poly(A) sites], even though they sometimes used a uridine (U) and occasionally a cytidine (C) or guanosine (G) [32]. For the dinucleotides at poly(A) sites, UA, CA, and GA were the most representative in 17, 10, and 2 of the species, respectively [32]. Interestingly, animals, dicotyledonous plants, and monocotyledonous plants had clear differences in C/G ratios at the poly(A) tail attachment position of the non-A-type poly(A) sites, suggesting that the C/G ratio at that position is involved in the evolutionary differentiation of these three large groups of living organisms [32]. Compared with the whole-genome base composition, the uracil (U) content in the poly(A) site upstream and downstream regions in both plants and animals is significantly overrepresented, with greater overrepresentation in the region upstream of the poly(A) site (the 3′ untranslated region [UTR]) than in the downstream region (the 3′ cleaved-off region [COR]) [33]. The 3′ UTR and 3′ COR regions, as functional units, minimized the difference between dicotyledonous and monocotyledonous plants, while the dicotyledonous and monocotyledonous genomes evolved into two extreme groups in terms of base composition [33]. However, these studies did not address the selection of motifs, motif abundances and locations, and base composition patterns (e.g., frequency peak locations) in the upstream and downstream regions of poly(A) sites in different kingdoms and subkingdoms.

In the present study, the term “poly(A) site” usually means “the poly(A) tail starting position” if there is no explicit indication about the exact position (i.e., the tail attachment position or the tail starting position). We mapped mRNA sequences available from the National Center for Biotechnology Information (NCBI, www.ncbi.nlm.nih.gov) to their corresponding genomes in most species whose genomes had been completely sequenced or at least assembled. In our previous study, we removed redundant copies of mRNA before mapping [32], whereas in this study, we removed not only redundant mRNA sequences but also redundant copies of poly(A) sites from duplicated or repetitive genes to ensure that each poly(A) site was unique, because of lack of evidence about whether every copy of multiple-copy genes was actually expressed. We also did certain analyses of genomic 3′UTR length and Illumina RNA-Seq reads to determine whether the identified motifs likely belonged to the coding region or the 3′UTR and whether the NCBI mRNA and Illumina reads have similar suitability in poly(A) site mapping. The most frequent motifs and their locations and relative genomic abundances, and the base composition characteristics surrounding (upstream of, at, and downstream of) the poly(A) sites in protists, fungi, plants, and animals were described.

## Results

### Upstream hexanucleotide motifs

Overall, the most frequent hexanucleotide motif in the upstream region within 50 nucleotides of the poly(A) tail starting position among the 31 species or species groups that were analyzed (Table 1 for species names; Additional file 1 for genome/chromosome GI list) was AAUAAA, even though it was not the most frequent hexanucleotide for some species (Table 2). This motif occurred as the most frequent hexanucleotide in all the animal species (53.9% of mRNAs on average), fungi, and monocotyledonous plants within the 50-bp upstream mRNA region (for motifs whose first base started in this region), even though the frequency was only 11.8% on average in the monocotyledonous plants (Table 2). The frequency rankings of the AAUAAA motif among all 4096 possible hexanucleotide motifs for each species are listed in Additional file 2. A-rich motifs of AAUAAA and AAAAUA showed certain predominance in the potato late blight pathogen *Phytophthora infestans* (Table 2). Interestingly, the most frequent motifs upstream of poly(A) sites were UGUUUU in *Trypanosoma cruzi* (an animal parasite protist), UGUAAC in *Chlamydomonas reinhardtii* (a single-celled green alga), and UAUUUU in three of the four dicotyledonous plant species (barrel medic [*Medicago truncatula*], poplar [*Populus trichocarpa*], and tomato [*Solanum lycopersicum*]) (Table 2).

**Table 1:** Common names or descriptions of the species analyzed

**Species**	**Common name or description**
**Fungi**	
*Aspergillus nidulans*	A soil fungus that can self-fertilize without a mating partner
*Cryptococcus neoformans*	A causal pathogen of cryptococcal disease
*Gibberella zeae*	A plant pathogen that causes Fusarium head blight
*Magnaporthe oryzae*	Rice blast fungus; rice rotten neck
*Marssonina brunnea*	A fungal pathogen of the *Populus* genus
*Metarhizium anisopliae*	A pathogen of insects
*Myceliophthora thermophila*	A fungus that degrades cellulose (used in biofuel production)
*Neurospora crassa*	A type of red bread mould
*Pichia pastoris*	A fungus widely used for producing recombinant proteins
*Saccharomyces cerevisiae*	Yeast used in baking and brewing
*Schizosaccharomyces pombe*	Fission yeast
*Sclerotinia sclerotiorum*	A white mould on plants
*Thielavia terrestris*	A soil-borne thermophilic fungus
*Yarrowia lipolytica*	A fungus that can use unusual carbon sources, e.g., hydrocarbons
**Protists**	
*Babesia bovis*	A single-celled protozoan parasite of cattle
*Blastocystis hominis*	An intestinal protozoan parasite
*Chlamydomonas reinhardtii*	A single-celled green alga
*Phytophthora infestans*	An oomycete, the causal agent of potato late blight
*Trypanosoma cruzi*	A parasite that causes trypanosomiasis disease in animals
**Plants**	
*Arabidopsis thaliana*	Arabidopsis
*Medicago truncatula*	Barrel medic, an annual yellow flower related to alfalfa
*Oryza sativa*	Rice
*Populus trichocarpa*	Western balsam poplar; California poplar; black cottonwood
*Solanum lycopersicum*	Tomato
*Solanum tuberosum*	Potato
*Sorghum bicolor*	Sorghum
*Zea mays*	Maize (corn)
**Animals**	
*Apis mellifera*	Honey bee
*Bos taurus*	Cattle
*Caenorhabditis elegans*	A free-living, transparent nematode (roundworm)
*Callithrix jacchus*	Common marmoset, a New World monkey
*Canis lupus familiaris*	Dog
*Ciona intestinalis*	Sea squirt
*Danio rerio*	Zebrafish
*Drosophila melanogaster*	Common fruit fly
*Equus caballus*	Horse
*Gallus gallus*	Chicken
*Homo sapiens*	Human
*Mus musculus*	Mouse
*Oryctolagus cuniculus*	Rabbit
*Pongo abelii*	Sumatran orangutan
*Rattus norvegicus*	Rat
*Sus scrofa*	Pig
*Taeniopygia guttata*	Zebra finch

**Table 2:** Most frequent tetranucleotide (4mer) motifs at polyadenylation [poly(A)] tail starting positions and most frequent hexanucleotide (6mer) motifs within 50 nucleotides upstream of poly(A) tail starting positions in each species

**Species/group**	**Mapped unique mRNA (No.)**	**Most frequent 4mer motif at the poly(A) sites**^**1**^	**%**	**Most frequent 6mer motif within 50 bases upstream**^**2**^	**%**
**Microorganisms**					
Fungi^3^	209	UUU**A**	7.2	AAUAAA	19.6
**Protists**^4^					
*Blastocystis hominis*	1 717	UUU**G**	5.01	AAGAAG	10.34
*Chlamydomonas reinhardtii*	176	UCC**A**	4.0	UGUAAC	31.3
*Phytophthora infestans*	63	CUC**A**	6.4	AAAAUA^5^	20.6
*Trypanosoma cruzi*	52	UUG**A**	19.2	UGUUUU	38.5
**Dicotyledonous plants**					
*Arabidopsis thaliana*	4 431	UUU**A**	4.9	AAUAAA	13.4
*Medicago truncatula*	136	UUC**A**	9.6	UAUUUU	17.7
*Populus trichocarpa*	1 371	UUU**A**	5.6	UAUUUU	12.6
*Solanum lycopersicum*	450	UUU**A**	8.2	UAUUUU	12.9
**Monocotyledonous plants**					
*Oryza sativa*	693	UUU**A**	7.4	AAUAAA	15.3
*Sorghum bicolor*	1 685	UUC**A**	5.5	AAUAAA	11.2
*Zea mays*	10 491	UUC**A**	4.6	AAUAAA	9.0
**Animals**					
*Apis mellifera*	187	AAU**A**	9.6	AAUAAA	53.5
*Bos taurus*	2 679	UUU**A**	3.7	AAUAAA	63.6
*Caenorhabditis elegans*	389	UUU**A**	11.6	AAUAAA	50.6
*Callithrix jacchus*	79	CUU**A**	10.1	AAUAAA	55.7
*Canis lupus familiaris*	108	UUU**A**	8.3	AAUAAA	68.5
*Ciona intestinalis*	287	UUU**A**	6.3	AAUAAA	48.4
*Danio rerio*	7 246	UUU**A**	6.0	AAUAAA	61.7
*Drosophila melanogaster*	954	AAC**A**	9.0	AAUAAA	59.0
*Equus caballus*	97	AUU**A**	7.2	AAUAAA	46.4
*Gallus gallus*	788	AAC**A**	4.6	AAUAAA	58.8
*Homo sapiens*	30 499	UUU**A**	5.0	AAUAAA	58.2
*Mus musculus*	8 709	UUU**A**	5.4	AAUAAA	61.2
*Oryctolagus cuniculus*	224	AUU**A**	5.4	AAUAAA	59.8
*Pongo abelii*	1 965	CUC**A**	5.9	AAUAAA	58.5
*Rattus norvegicus*	14 263	UUU**A**	6.4	AAUAAA	56.0
*Sus scrofa*	8 114	GAC**A**	8.1	AAUAAA	40.5
*Taeniopygia guttata*	808	UGC**A**	4.2	AAUAAA	59.9
**Animal average**	**4 116**			**AAUAAA**	**53.9**

^1^The last nucleotide in bold in the tetranucleotide is the poly(A) tail starting position of the poly(A) site.

^2^The AAUAAA motif could be found in 11.8% of unique poly(A) sites in monocotyledonous plants and in 11.9% of unique poly(A) sites in all plants (average of all four dicotyledonous plants and all three monocotyledonous plants together). The frequencies of these motifs are all significantly higher than the mean frequencies of hexanucleotide motifs in the region according to chi-test (P < 0.000001).

^3^Pooled from the 14 fungal species listed in Table 1.

^4^The protist *Babesia bovis* was found to also have A predominance at the poly(A) tail starting position: 18 of 23 mapped unique poly(A) sites were A (data not shown).

^5^AAUAAA was among the most frequent upstream motifs (11.11%). In EST-based mapping [207 unique poly(A) sites], AAUAAA was the most frequent but the frequency was only 16.91%, and AAAAUA became the second most frequent. The analysis results suggested a weak predominance of AAUAAA and some other A-rich motifs including AAAAUA.

### Upstream pentanucleotide motifs

In the 48-base region two bases upstream of the poly(A) tail starting position, the five most frequent pentanucleotide motifs were found to be A-rich and to lack G in most animals, and to be U-rich and to have G in all plants (Additional file 3). The AUAAA motif was found to be the most frequent pentanucleotide in animals, with a frequency of over 70% in the nematode *C. elegans* and in dog (*Canis lupus familiaris*). Compared with animals, plants did not have any very frequent motifs. For example, the most frequent pentanucleotide motif (UGUUU) in maize (*Zea mays*) accounted for only 19.77% of mRNAs (Additional file 3).

### Tetranucleotide motifs at the poly(A) site

For the identified tetranucleotide motifs, whose last nucleotide covered the pre-mRNA poly(A) tail starting position, UUUA was the most predominant (Table 2). This motif was the most frequent in three of the seven plant species and eight of the 19 animal species, but the actual percentage of UUUA-carrying mRNA was still very low in the whole mRNA population (Table 2). None of the species used ACCA as the most frequent motif covering the poly(A) tail starting position (Table 2).

### Base composition patterns around poly(A) sites in protists

Note that in the text descriptions, U is used for RNA, but T (for DNA or genes) is used as the default in the sequence logo (seqlogo) graphs.

**Figure 1:**
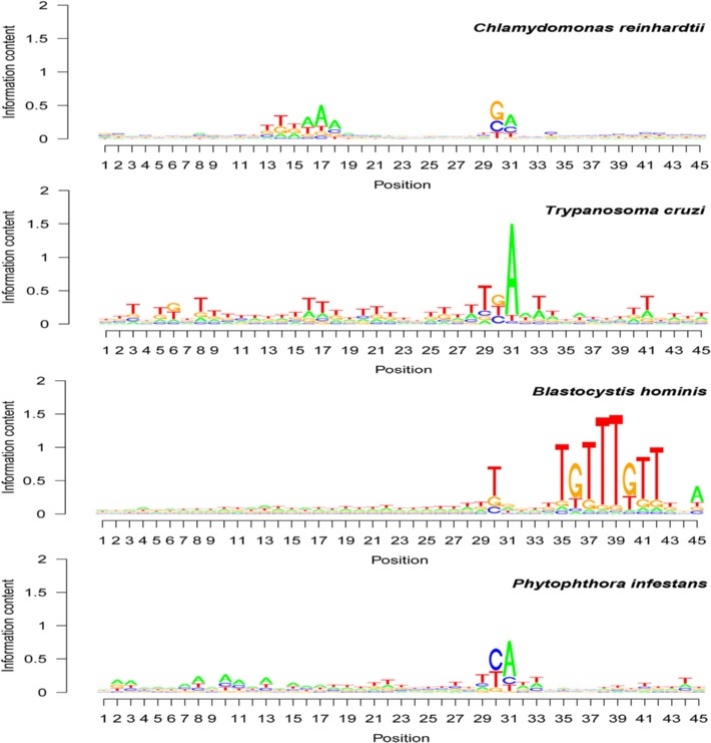
**Average base abundance at each position of the 45-nucleotide polyadenylation [poly(A)] site regions in protists.** Sequence logo (seqlogo) position from left to right: 5′ to 3′ of the DNA sequences. Position 31 in the seqlogo graphs is the poly(A) site—the poly(A) tail starting position. Note that i) the base abundance pattern was very different in each of the protist species, ii) the poly(A) site was mainly an A in *Trypanosoma cruzi*, *Phytophthora infestans*, and *Chlamydomonas reinhardtii* but was not an A in *Blastocystis hominis*, and iii) the signature region could be upstream of (*C. reinhardtii*), at (*T. cruzi*), or downstream of (*B. hominis*) the poly(A) site.

Base abundance around the poly(A) site regions in four protists is presented in seqlogo format (Figure 1). Three protists, namely *C. reinhardtii*, *P. infestans*, and *T. cruzi*, were found to have A predominance at the poly(A) site, that is, the poly(A) tail starting position (position 31 in the seqlogo graphs in Figure 1). The protist *Babesia bovis* was found to also have A predominance at the poly(A) tail starting position: 18 of the 23 mapped unique poly(A) sites were A (data not shown). This A abundance at the poly(A) site was absent (A: 30%; G: 35%) in *B. hominis* and relatively weak in *C. reinhardtii*.

At the poly(A) tail attachment position (position 30 in the seqlogo graphs in Figure 1), *C. reinhardtii* and *T. cruzi* used mainly G [i.e., GA dinucleotide at the poly(A) site], but *B. hominis* used U (T for genes) (Figure 1). In *B. hominis*, G was the second most abundant at this poly(A) tail attachment position. In *P. infestans*, C was the most abundant and U was the second most abundant, but the A and U abundances were quite similar (Figure 1). *C. reinhardtii* showed a U-rich region (see seqlogo positions 13–15 in Figure 1) and an A-rich region (see seqlogo positions 15–18 in Figure 1). These two regions jointly form a U-A–rich island in the seqlogo graphs (Figure 1). The poly(A) site upstream region in *T. cruzi* was essentially U-abundant, with G abundance at a few base positions (Figure 1). *P. infestans* showed an A-rich region (see seqlogo positions 8–17 in Figure 1) and a U-rich region (see seqlogo positions 18–29 in Figure 1). Clearly, the upstream base composition abundance pattern is not conserved among the four protists.

The downstream region was found to be essentially U-rich to a certain degree in *P. infestans* and *T. cruzi*. The most obvious pattern is that *B. hominis* had a strong U- and G-rich region as UGUUUGUU in RNA (or TGTTTGTT in DNA) (see seqlogo positions 35–45 in Figure 1). The base abundance among mRNA sequences in this downstream region was found to be clearly species-specific in the four protists analyzed.

### Base composition patterns around poly(A) sites in plants

**Figure 2:**
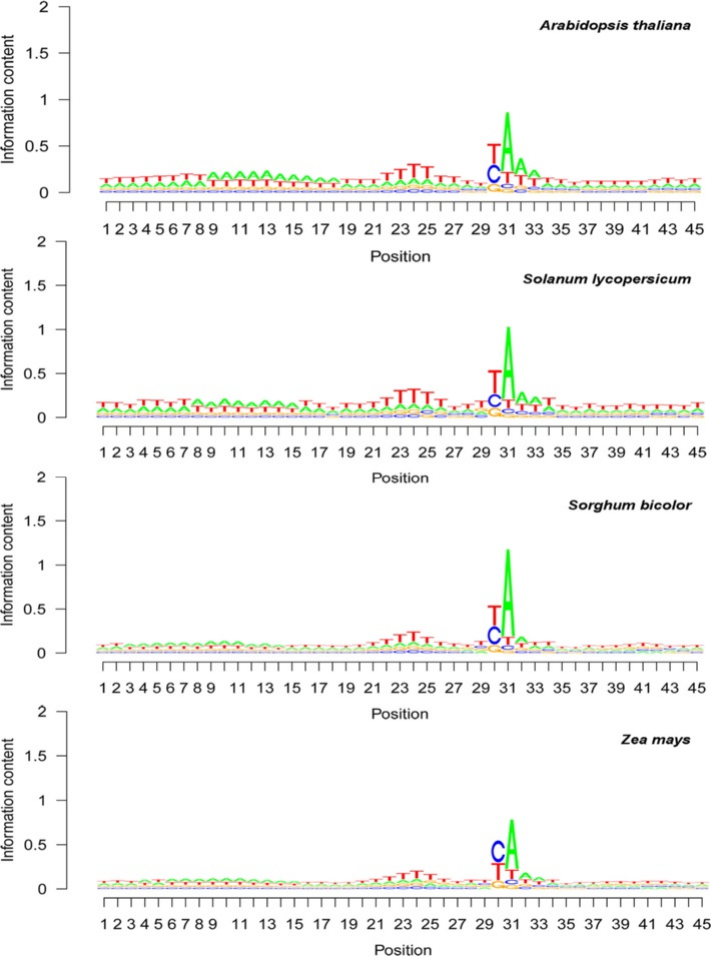
**Base composition pattern around polyadenylation [poly(A)] sites in Arabidopsis (*Arabidopsis thaliana*), tomato (*Solanum lycopersicum*), sorghum (*Sorghum bicolor*), and maize (*Zea mays*).** Sequence logo (seqlogo) graphs were drawn based on the average base composition among mapped poly(A) sites. Position 31 in these seqlogo graphs is the poly(A) tail starting position, corresponding to position 1 in Figure 5. Position 30 in these seqlogo graphs is the poly(A) tail attachment position, corresponding to position −1 in Figure 5. Positions 10 and 24 in these seqlogo graphs correspond to positions −21 and −7 in Figure 5. Note that the upstream A-rich region had its peak at seqlogo position 10 (i.e., position −21 in Figure 5) and that the upstream U-rich region had its peak at seqlogo position 24 (i.e., position −7 in Figure 5). Note that i) the poly(A) tail attachment position was usually a U, although in some species, such as maize, C and U were approximately equal, and ii) the base abundance regions from left to right showed a U-A-U-A-U pattern, where the second A in this pattern is the poly(A) site.

Base composition patterns around poly(A) sites showed high similarity among all seven plant species analyzed, even though each species had its own specificity, as shown in the four plants presented in Figure 2. The poly(A) site was predominantly an A (see seqlogo position 31 in Figure 2). The poly(A) tail attachment position was mainly a U (or T for the genome) in most species, except in maize (see seqlogo position 30 in Figure 2). Guanosine was always the least frequent at both the poly(A) tail starting position and the poly(A) tail attachment position (Figure 2).

The upstream region of the poly(A) site was very similar among the plant species. In the seqlogo graphs, there was clearly a U-rich region (see the first bases in the seqlogo graphs in Figure 2), followed by an A-rich region with a peak frequency location (seqlogo position 10 in Figure 2) at a distance of 21 bases from the poly(A) site. The remaining part up to the poly(A) tail attachment position was U-rich (T-rich for genes). The downstream region was essentially U-rich (T-rich for genes). The overall base composition pattern from the 201-base region analyzed [in the order of upstream, poly(A) site, and downstream] is “upstream U-rich—upstream A-rich—upstream U-rich—SiteA—downstream U-rich”. This pattern can be written as U-A-U-SiteA-U or U-A-U-A-U.

### Base composition patterns around poly(A) sites in animals

**Figure 3:**
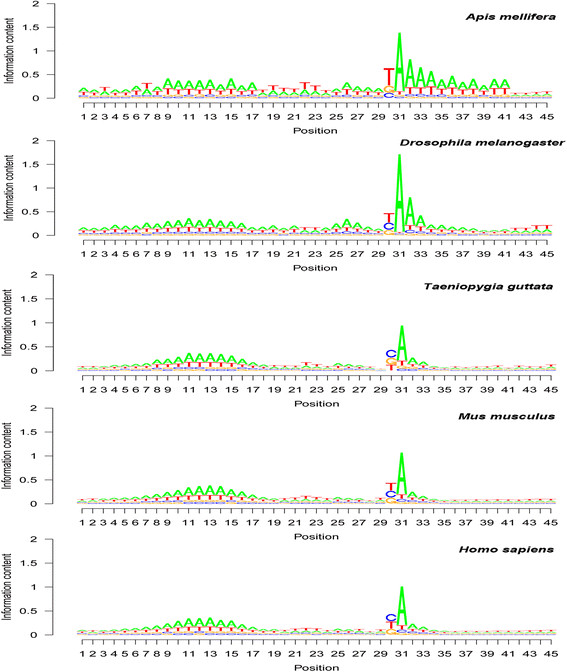
**Base composition pattern around polyadenylation [poly(A)] sites in honey bee (*Apis mellifera*), common fruit fly (*Drosophila melanogaster*), zebra finch (*Taeniopygia guttata*), mouse (*Mus musculus*), and human (*Homo sapiens*).** Sequence logo (seqlogo) graphs were drawn based on the average base composition among mapped poly(A) sites. Position 31 in these seqlogo graphs is the poly(A) tail starting position (corresponding to position 1 in Figure 5). Position 30 in these seqlogo graphs is the poly(A) tail attachment position (corresponding to position −1 in Figure 5). Positions 13 and 22 in these seqlogo graphs correspond to positions −18 and −9 in Figure 5. Note that i) the poly(A) site showed a strong A predominance, and ii) the predominance at the poly(A) tail attachment position was U or C, depending on the species.

Base composition patterns around poly(A) sites showed high similarity among all 17 animal species analyzed, even though each species had its own specificity, as shown in the five animals presented in Figure 3. The poly(A) site was predominantly an A (see seqlogo position 31 in Figure 3). The poly(A) tail attachment position was mainly a U or C (see seqlogo position 30 in Figure 3). At this poly(A) tail attachment position, U was very clearly predominant in the two insects (honey bee [*Apis mellifera*] and common fruit fly [*D. melanogaster*]), but the U and C frequency differences were not very large in mouse (*Mus musculus*) and human (*H. sapiens*) mRNAs (Figure 3).

The following common features in the upstream region for most animal species were seen: there was clearly a U-rich region (see the first bases in the seqlogo graphs in Figure 3), followed by an A-rich region with, usually, a peak frequency location (seqlogo position 13) at a distance of 18 bases from the poly(A) site (Figure 3). Then, there was a U-rich region with the frequency peak at seqlogo position 22, which was nine bases from the poly(A) site (Figure 3). Unlike the patterns in plants, the patterns in animals had an A-rich region with a frequency peak location at approximately the fifth base upstream of the poly(A) site (Figure 3). The downstream region was essentially U-rich (T-rich for genes). Both the upstream and downstream regions around poly(A) sites in the insects honey bee and common fruit fly were found to be richer in A than were those regions in other animals (Figure 3). The overall base composition pattern for this region [in the order of upstream, poly(A) site, and downstream] was “upstream U-rich—upstream A-rich—upstream U-rich—upstream A-rich and SiteA—downstream U-rich” if the poly(A) site and the A-rich element in the closely located upstream region were presented jointly as the same A-rich region. This pattern, similar to the one for plants, can be written as U-A-U-SiteA-U or U-A-U-A-U.

### Base composition patterns in the poly(A) site region—comparison between dicotyledonous plants and monocotyledonous plants

**Figure 4:**
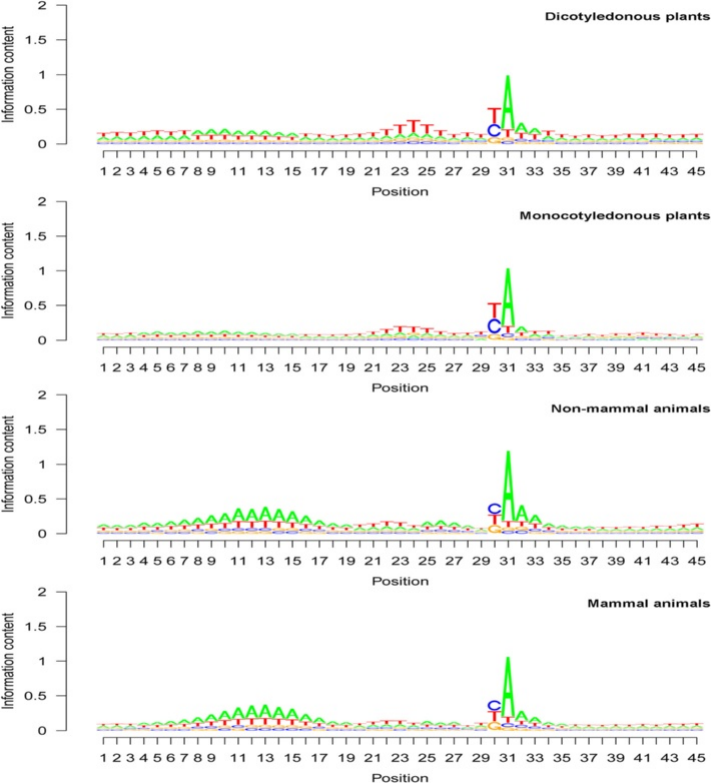
**Base composition for each position around the polyadenylation [poly(A)] site for dicotyledonous plants, monocotyledonous plants, non-mammal animals, and mammal animals.** See Table 3 for the values of the major base frequency peaks and the names of species in each group. Sequence logo (seqlogo) graphs were drawn based on the average base composition among mapped poly(A) sites. Position 31 in these seqlogo graphs is the poly(A) tail starting position (corresponding to position 1 in Figure 5). Position 30 in these seqlogo graphs is the poly(A) tail attachment position (corresponding to position −1 in Figure 5). Positions 13 and 22 in these seqlogo graphs correspond to positions −18 and −9 in Figure 5. Note that i) the locations of the upstream A peak and upstream U peak are identical between dicotyledonous plants and monocotyledonous plants and between non-mammal animals and mammals, and ii) the upstream A abundance peak (position 13 in animals) and U abundance peak (position 22 in animals) are at the same locations between subkingdoms but are two or three bases apart between the plant and animal kingdoms.

The frequency peak locations in the average base composition pattern U-A-U-SiteA-U or U-A-U-A-U were found to be identical in both dicotyledonous plants and monocotyledonous plants (Table 3; Figure 4). The first U and last U of this U-A-U-A-U pattern were two U-rich regions that did not usually have a specific frequency peak. The internal A- or U-rich regions had a clear peak at specific locations. This U-A-U-A-U pattern was found to have a U-rich upstream region without a clear peak; an A-rich region with a peak at seqlogo position 10 (Figure 4), which is at a distance of 21 bases from the poly(A) site; a U-rich region with a peak at seqlogo position 24 (Figure 4), which is seven bases from the poly(A) site; the poly(A) site (seqlogo position 31 in Figure 4); and a U-rich region one or two bases after the poly(A) site. The second upstream U peak location showed minor differences between the two subkingdoms: a strong U peak at the seventh base upstream of the poly(A) site in dicotyledonous plants and jointly at the seventh and eighth bases in monocotyledonous plants.

**Table 3:** Base abundance values at the peak locations of the upstream A-rich region and the upstream U-rich region as compared between plants and animals

**Subkingdom**	**Upstream A peak (at position −21)**	**Upstream A peak (at position −18)**	**Upstream U peak (at position −9)**	**Upstream U peak (at position −7)**
Dicotyledonous plants^1^	**41.03 B**	39.69 B	47.38 A	**56.18 A**
Monocotyledonous plants^2^	**37.84 B**	35.32 C	44.37 AB	**47.48 B**
Non-mammal animals^3^	50.14 A	**53.89 A**	**42.85 B**	35.36 C
Mammals^4^	49.12 A	**54.67 A**	**42.14 B**	35.10 C
Mean	44.53	45.89	44.19	43.53

**Figure 5:**
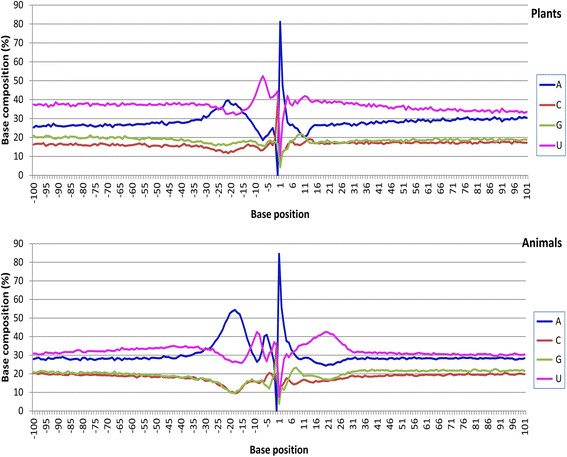
**Average base abundance at each position of the 201-nucleotide polyadenylation site region, showing the U-A-U-SiteA-U base abundance pattern in plants and animals.** Plants: *Arabidopsis thaliana*, *Medicago truncatula*, *Oryza sativa*, *Populus trichocarpa*, *Solanum lycopersicum*, *Sorghum bicolor*, and *Zea mays*. Animals: *Bos taurus*, *Danio rerio*, *Drosophila melanogaster*, *Gallus gallus*, *Homo sapiens*, *Mus musculus*, *Pongo abelii*, *Rattus norvegicus*, and *Taeniopygia guttata*. Note that the base composition showed several clear differences between plants and animals. For example, the upstream A peak was lower but the U peak was higher in plants in comparison with animals. The shape of the downstream U peak was flat in plants but more pointed in animals. There was also a small A peak at position −3 in plants and position −5 in animals in addition to the U-A-U-SiteA-U (or U-A-U-A-U) base abundance pattern.

The bold values are the peak values in Figure 5. The position (see Figure 5) is indicated in terms of nucleotides from the polyadenylation [poly(A)] tail starting position, i.e., the first A of the poly(A) tail.

^1^Dicotyledonous plants: *Arabidopsis thaliana*, *Medicago truncatula*, *Populus trichocarpa*, and *Solanum lycopersicum*.

^2^Monocotyledonous plants: *Oryza sativa*, *Sorghum bicolor*, and *Zea mays*. The upstream U peak in monocotyledonous plants was shared between positions −7 and −8 (see Figure 5 for the positions).

^3^Non-mammal animals: *Danio rerio*, *Drosophila melanogaster*, *Gallus gallus*, and *Taeniopygia guttata*.

^4^Mammals: *Bos taurus*, *Homo sapiens*, *Mus musculus*, *Pongo abelii*, and *Rattus norvegicus*.

Note that plants and animals were found to be different in terms of peak locations. At these locations, the subkingdom base frequency values are more similar within the same kingdom than between kingdoms. Means with the same letter are not significantly different (*P* < 0.05) according to ANOVA and Duncan’s multiple range test.

### Base composition patterns in the poly(A) site region—comparison between non-mammal animals and mammals

The overall base composition pattern in animals was found to be highly similar to the pattern in plants, despite differences in exact frequency values and base positions (Figure 4). Both non-mammal animals and mammals were found to have the following base composition pattern (Table 3; Figure 4): a strong A peak at the poly(A) tail starting position; predominance of C and U (nearly identical, 35%–37%) at the poly(A) tail attachment position; a moderate U peak at the ninth position upstream (Table 3; seqlogo position 22 in Figure 4) of the poly(A) site; a broad and strong A peak (seqlogo position 13 in Figure 4) at a distance of 18 bases from the poly(A) site; and U richness in all the remaining upstream and downstream regions. This pattern was also very typical, as was the summarized U-A-U-SiteA-U or U-A-U-A-U pattern. In animals, a weak or moderate A peak was found at five bases upstream of the poly(A) site; such a peak was not obvious in plants.

### Statistical comparison between plants and animals—position and frequency of base frequency peaks

The base abundance peaks in the 201-base region of the poly(A) sites are presented in Figure 5. Most peaks were within a 30-base distance from the poly(A) site (Figure 5). The average position among mRNAs for any given base frequency peak in this pattern was identical between non-mammal animals and mammals (Figure 4). Plants and animals showed minor differences in several peak locations: the upstream A peak was at −21 in plants but −18 in animals and was therefore three bases closer to the poly(A) site in animals than in plants (Table 3; Figure 5). However, the upstream U peak was found to be two bases farther from the poly(A) site in animals than in plants. Animals showed a strong and very obvious U peak in the downstream region 10 to 30 bases from the poly(A) tail starting position, but this region lacked a sharp U peak in plants even though the U content was found to also be high in this region (Table 3; Figure 5).

The peak values (average values among species) for base frequency were found to be very similar between dicotyledonous plants and monocotyledonous plants and between non-mammal animals and mammals according to ANOVA and Duncan’s multiple range test (Table 3). Plants and animals showed significant differences in base frequency at each peak (Table 3).

**Figure 6:**
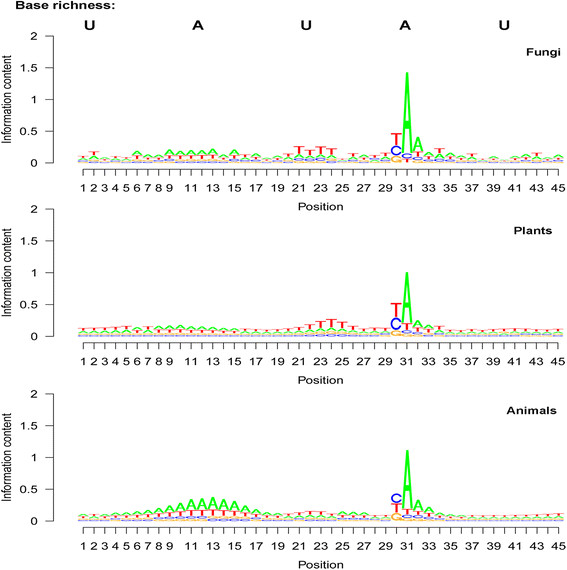
**Patterns of average base composition around polyadenylation [poly(A)] sites in fungi, plants, and animals.** Sequence logo (seqlogo) graphs were drawn based on the average base composition among mapped poly(A) sites. Position 31 in these seqlogo graphs is the poly(A) tail starting position (corresponding to position 1 in Figure 5). Position 30 in these seqlogo graphs is the poly(A) tail attachment position (corresponding to position −1 in Figure 5). Positions 13 and 22 in these seqlogo graphs correspond to positions −18 and −9 in Figure 5. Note that the base composition pattern was highly conserved among the fungal, plant, and animal kingdoms. In the U-A-U-A-U pattern, the first U was the relatively far-upstream U-rich region (1st UUR), the first A was the upstream A-rich region (UAR), the second U was the upstream U-rich region (2nd UUR), the second A was the A at the poly(A) site (SiteA), and the last U was the downstream U-rich region (DUR). The 70-base segment upstream of this seqlogo region was U-rich in fungi, plants, and animals (partly shown in Figure 5).

High similarity in overall base composition patterns was found among fungi, plants, and animals (Figure 6). They all showed strong A predominance at the poly(A) site, U or C predominance at the poly(A) tail attachment position, and a general U-A-U-A-U base composition pattern in the poly(A) site region, even though the pattern in fungi was not as typical as it was in plants and animals (Figure 6). In this general U-A-U-A-U base composition pattern of these three kingdoms, the first U is the long and far upstream U-rich region (1st UUR), the first A is the upstream A rich region (UAR), the second U is the upstream U-rich region (2nd UUR), the second A is the A at the poly(A) site (SiteA), and the last U is the downstream U-rich region (DUR). The 1st UUR and the DUR covered relative longer regions compared with other regions in this pattern (Figure 5) and were less obvious in predominance in fungi than in plants and animals (Figure 6; also see Figure 5 for plants and animals). The UAR on the seqlogo graphs was more prominent in animals than in plants and fungi (Figure 6); whereas, the 2nd UUR on seqlogo graphs was more obvious in fungi and plants than in animals (Figure 2).

### Comparison between plants and animals—base compositions in the upstream region 30 to 100 nucleotides away from the poly(A) site

For plants, the upstream region 30 to 100 nucleotides away from the poly(A) site was U> > A> > G > C in terms of the base content. For animals, however, this order was U > A> > G ≥ C (Figure 5). For the downstream region 30 to 100 nucleotides away from the poly(A) site, the base composition for plants was in the pattern U> > A> > G ≥ C but for animals was U > A> > G > C (Figure 5).

### Frequencies of known downstream elements

The frequencies of various known elements within 50 bases downstream of the poly(A) site were computed in the unique poly(A) site pool in the present study (Additional file 4). The motif UUAUUU [19] was detected from 9.97% poly(A) sites, the element CCUCCC [26] from 1.18% poly(A) sites, UGUGUG [19] from 5.38% poly(A) sites, the GUGUGU portion of GUGUGUGUUUG [28] from 4.46% poly(A) sites, the UGUUUG portion of GUGUGUGUUUG [28] from 5.68% poly(A) sites, and the AUGCGU portion of AUGCGUUCCUCGUCC [29] from 0.56% poly(A) sites. The UUAUUU, UGUGUG, GUGUGU, and UGUUUG elements were significantly (P < 0.01) more frequent than the hexamer averages, CCUCCC was more or less frequent depending on species, and AUGCGU was either significantly less frequent or not significant from the hexamer mean frequency in the downstream region of the poly(A) site. Compared with the upstream most frequent motifs (Table 2), these downstream motifs/elements were much less frequent (Additional file 4). Further research is required to verify whether these less frequent downstream motifs function only in specific groups of genes and regulate differential expression of these genes.

### Most frequent motifs around the poly(A) site in humans

**Figure 7:**
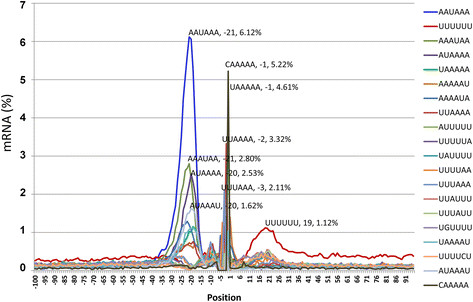
**Most frequent 20 hexamer motifs in the poly(A) site region in 30499 unique poly(A) sites mapped to the human genome.** Order in the peak point labeling: motif sequence, position, and frequency at the single specific position in the mapped poly(A) site regions (pre-mRNA). The AAAAAA motif was the highest at position 1, but the frequencies or AAAAAA and UUUUUU were inflated in counting, for example, a sequence of 8 A’s was counted as three copies of AAAAAA motifs when counting motifs at each position. It is why AAAAAA was not showing in the figure here. The UUUUUU motif listed here was to show the location of the frequency peak. The most abundant five motifs, in terms of the values of the highest peaks were AAUAAA, CAAAAA, UAAAAA, UUAAAA, and AAAUAA. The top five motifs, in terms of the number of accumulated copies along the 201-base region were AAAAAA (135.84%), AAUAAA (75.64%), UUUUUU (73.87%), AAAUAA (46.93%), AUAAAA (41.40%). Note that the most frequent motifs are mainly on three places: the −21 position region (upstream A-rich element; mainly AAUAAA), the poly(A) site, and the downstream U-rich region with peak location approximately at +19. No highly abundant hexanucleotide motifs were detected from other regions.

To verify the results of downstream motif frequencies, we determined the top 20 most frequent motifs in the 201-base region around the poly(A) site from the 4096 hexanucleotide motifs in 30499 unique poly(A) sites mapped to the human genome (Figure 7). The most abundant five motifs, in terms of the values of the highest peaks were AAUAAA, CAAAAA, UAAAAA, UUAAAA, and AAAUAA. The top five motifs, in terms of the number of accumulated copies along the 201-base region were AAAAAA (135.84%), AAUAAA (75.64%), UUUUUU (73.87%), AAAUAA (46.93%), AUAAAA (41.40%) (Figure 7). All the most frequent motifs were mainly concentrated in three regions: the −21 position area, the poly(A) site, and the downstream U-rich region with peak location approximately at +19. No highly abundant hexanucleotide motifs were detected from other regions (Figure 7).

### Motif locations in pre-mRNA in different species

**Figure 8:**
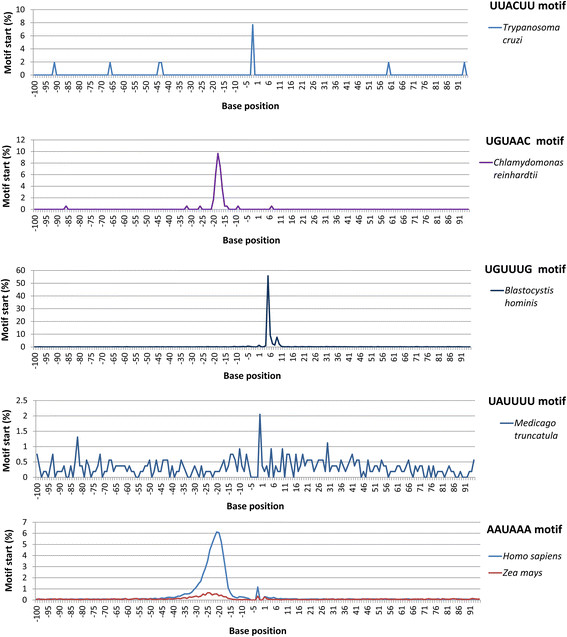
**Motif location frequency around polyadenylation [poly(A)] sites.** The frequency (%) is the percentage of mRNAs that have the motif’s first nucleotide starting from the position. The UUACUU motif in *Trypanosoma cruzi* started at position −2 [with the nucleotide A directly at the poly(A) site]. The UGUAAC motif in *Chlamydomonas reinhardtii* was at position −18 [i.e., the poly(A) site and the motif’s last base were separated by 12 bases]. The UGUUUG motif in *Blastocystis hominis* was located at downstream position 5--the poly(A) tail attachment position and the motif’s first base were separated by four bases [i.e., the poly(A) site and the motif were separated by three bases]. The UAUUUU motif in *Medicago truncatula* had a peak value at position −1, with the nucleotide A of the motif located at the poly(A) site, but the motif was not concentrated at one location. The AAUAAA motif had its peak value at position −21 in human and most animals, position −22 in Arabidopsis (data not shown), and position −24 in maize (but the peak was weak). Note that each of the predominant motifs was found to occur usually at a specific location, whether upstream of, at, or downstream of the poly(A) site. The AAUAAA motif corresponded to the A-rich upstream region in both maize (representing the plant kingdom) and human (representing the animal kingdom), and the location of AAUAAA was highly conserved, with a difference of a few bases between plants and animals.

The predominant motifs were found to occur usually at a specific location, whether upstream of, at, or downstream of the poly(A) site (Figure 8). The AAUAAA motif corresponded to the A-rich upstream region in both maize (representing the plant kingdom) and human (representing the animal kingdom) (Figure 8).

The UGUAAC motif is a predominant upstream motif in *C. reinhardtii.* Its frequency peak value location was at position −18, that is, the first U of UGUAAC was at this 18th position upstream. Since this is a hexamer, the distance between the last C of this motif and the poly(A) site was 12 bases. In the mapped unique poly(A) sites, 9.7% of the sites had the UGUAAC motif at this base. Nearly all the UGUAAC motifs were found within two bases of this location. It should be noted that the predominant motif was not necessarily the direct reading of the most predominant bases. In this upstream region in *C. reinhardtii*, the seqlogo reading was TTTAAA (or UUUAAA for RNA) (Figure 1), but the motif or called element at the exact location was actually UGUAAC (Figure 8). The frequency of the UUUAAA motif at this exact location was only 1.1% (data not shown). This is because an mRNA sequence could have U’s or A’s at these locations but not with the UUUAAA motif in the same single mRNA. This difference between motif and predominant base may suggest that not only the specific motif but also base predominance at a specific region can play a role in determining the poly(A) site. A similar phenomenon was also observed for the downstream U-rich region. The downstream region from the poly(A) site was found be strongly U-rich in both plants and animals, but the UUUUUU motif was not the strongest at any single location in the downstream region in most species. It is likely that the base composition pattern (or regions rich in certain nucleotides) and the motif type (upstream elements or downstream elements) should be viewed as two molecular traits, even though the base frequency peak and predominant motif location usually overlap, as was the case with the AAUAAA motif.

The AAUAAA motif was a typical upstream motif, usually located at position −21 in animals and −24 or −25 in plants such as maize, as shown in Figure 8 [meaning that between the motif and the poly(A) site, there were 15 bases in animals and 17 or 18 bases in plants]. The AAUAAA motif was also more abundant at position −21 in fungi (data not shown). Even though the AAUAAA motif frequency was very low in maize and fungi (Table 2) and very high in animals, the location of AAUAAA was found to be highly conserved.

At the poly(A) site, there was a UUACUU motif in *T. cruzi*, which had the first U of this motif at two bases upstream of the poly(A) site (Figure 8). This means that the base A in this UUACUU motif is the exact poly(A) site [i.e., poly(A) tail starting position]. In plants, another motif, namely UAUUUU, was also found to be mainly at the poly(A) site in some dicotyledonous plants such as *M. truncatula*; however, this motif was not as concentrated at one location as the UUACUU motif was (Figure 8).

In the downstream region, the strongest motif was found to be UGUUUG or UGUUUGUU (or TGTTTGTT for DNA) in *B. hominis*, whose first U of the motif was at position 5, that is, at a distance of four bases downstream of the poly(A) site (Figure 8). In the downstream region, AAAAAA was often counted as the most frequent in animals, but it is unknown how much effect conflation for the AAAAAA frequency from internal priming had at this stage.

### Most frequent tetramer and hexamer motifs in whole genomes

In order to know whether the identified motif frequencies were random reflections of their average frequencies in the whole genome, we analyzed all the tetramer and hexamer motifs in the chromosomes of *S. pombe*, *A. thaliana*, *Oryza sativa* (rice), *Z. mays*, *D. melanogaster*, *C. elegans*, and *H. sapiens* (Additional file 5). If the uniform motif AAAA or TTTT is not listed, the highest frequency of the non-uniform tetramer motif was 1.9%, and the most frequent tetramer motif was found to be AAAU in *S. pombe* chromosome 1. The most frequent poly(A) site tetramer UUUA in RNA (Table 2) was found to be lower, at 1.1% in genome. For hexamer motifs, no motifs except for AAAAAA and TTTTTT reached 0.2% (Additional file 5). The count of motifs with only a single type of nucleotide in Additional file 5 and the count of motif copy numbers in the genome by DNAStar could be conflated. For example, a DNA sequence of AAAAAAA (seven A’s) could be counted as two AAAAAA motifs. Therefore, the real, non-overlapping motif frequencies of AAAAAA or TTTTTT could be much lower than the counted frequency (0.2%). The highest frequency of the AAUAAA motif was 0.1% in three common fruit fly chromosomes. The AAUAAA motif was not among the top eight most frequent motifs and was usually far less than 0.1% in most cases. Clearly, the major hexamer motifs presented in Table 2 and Figures 2, 3, 4, 5 and 6 were approximately a few hundred times more frequent in the poly(A) site region than in the whole genome and much more frequent than in the other regions of the genome.

### UTR length

**Figure 9:**
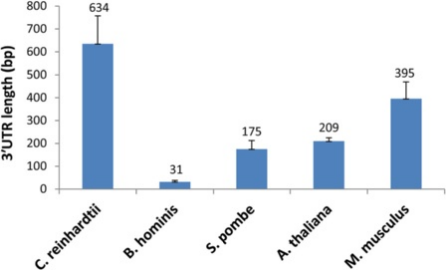
**Average length of the 3′ untranslated region (UTR) of 10 mRNA sequences per species for*Chlamydomonas reinhardtii*(alga),*Blastocystis hominis*(protozoan parasite),*Schizosaccharomyces pombe*(fission yeast),*Arabidopsis thaliana*(Arabidopsis), and*Mus musculus*(mouse).** Note that the 3′UTR length varied greatly among species, but there is no mixture between the poly(A) site signature motifs presented in Figure 8 and stop codons.

The length of the 3′UTR of several species was reviewed previously, and average lengths of 273 nucleotides in Liliopsidae, 207.7 nucleotides in other viridiplantae, 607.3 nucleotides in rodent, and 1027.7 nucleotides in human were found [34]. Subunits of the cleavage factor I_m_ complex may regulate 3′UTR length [35]. However, information on 3′UTR length for most of the species that we analyzed in the present study was still missing. Although 3′UTR length was not the focus of the present study, we conducted some preliminary analyses of 3′UTR length using 10 polyadenylated mRNAs in order to assist with data interpretation for the base composition patterns identified in this study. The average UTR length varied considerably among species (*C. reinhardtii, S. pombe*, *A. thaliana*, and *M. musculus*), but all lengths were much longer than the 100-bp poly(A) site upstream regions analyzed, except the region in *B. hominis* (Figure 9). As described in the previous figures (Figures 2, 3, 4, 5, 6, 7 and 8), most of the motifs and the base abundance bias regions were within a 30-base distance from the poly(A) sites. *Blastocystis hominis* UTRs were only 31 bp on average and occasionally UTR-less [e.g., the stop codon and poly(A) tail overlap in mRNA FQ822747.1], but the most obvious motif in the poly(A) region was after the poly(A) site. Therefore, the predominant motifs and base frequency peaks identified in this study were mainly from the 3′UTR or 3′COR, but not motifs of the translation stop codons.

#### Comparison between NCBI mRNA and illumina RNA-seq reads in poly(A) site mapping

**Figure 10:**
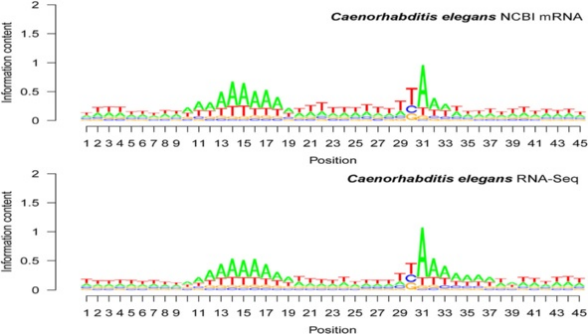
**Base composition patterns around polyadenylation [poly(A)] sites: comparison between NCBI mRNA sequences and Illumina TruSeq reads.** Note that the poly(A) site and the bases located immediately downstream of it were richer in A in Illumina reads than in NCBI mRNA sequences, suggesting a higher proportion of internal priming and likely also artifact poly(A) tails created by RNA/cDNA fragmentation in the RNA-Seq (TruSeq) reads than in the NCBI mRNA sequences.

In order to increase the number of mapped poly(A) sites, we conducted mapping using deep-sequencing RNA-Seq reads in several species. The comparison of the results between NCBI mRNA and Illumina mRNA TruSeq reads in the nematode *C. elegans* is presented in Figure 10. The poly(A) site and the immediately downstream six-base region is richer in A in Illumina reads-based mapping than in NCBI mRNA-based mapping (see seqlogo positions 31 to 37 in Figure 10). The predominance of U (seqlogo position 30 in Figure 10) was lower in RNA-Seq reads than in mRNA. It is known from Table 2 that the most frequent tetramer motif was UUUA at the poly(A) site in *C. elegans* (Table 2; seqlogo positions 28 to 31 in Figure 10). The UUUA motif was only 7.08% in 579 Illumina RNA-Seq reads, which was significantly less frequent than the 11.60% in 389 mRNA-mapped poly(A) sites (*P* < 0.001, according to the chi-test). In Figure 10, T is lower (meaning less predominant) in the RNA-Seq seqlogo than in the NCBI mRNA seqlogo. Therefore, compared with the NCBI mRNA-based poly(A) sites, the Illumina RNA-Seq reads-based poly(A) sites were richer in A in the poly(A) site region, and the poly(A) site signal motif UUUA was less frequent. These results suggest that Illumina TruSeq RNA-Seq reads were more sensitive to internal priming from A-rich regions.

## Discussion

### NCBI mRNA and illumina RNA-seq reads

Enhancement of the average A content for the 3′COR was detected in potato (*S. tuberosum*), honey bee, and common fruit fly in Illumina RNA-Seq-based mapping [33]. The present study determined the degree of A richness at each position in the poly(A) site region as shown by seqlogo graphs (Figure 10), confirming the sensitivity to internal priming and fragmentation-created artificial poly(A) tails in Illumina-reads-based mapping. Data from the previous study [33] and the present study support each other. The major advantages of using the NCBI mRNA database for the mapping and quantitative analysis of poly(A) sites are as follows: i) the quality of sequences is high, because researchers usually verify the sequences by repeated sequencing of the same cDNA clone or clones before submission, particularly if there is a poly(A) tail in the sequence; ii) mRNA sequences are available for many species because of the accumulation of sequences over decades; and iii) the database contains long sequences. The major disadvantages of using the NCBI mRNA database for this purpose is its smaller number of sequences, in comparison with deep-sequencing reads. The major advantage of using Illumina RNA-Seq reads for the mapping and quantitative analysis of poly(A) sites is the larger number of sequence reads, in comparison with NCBI mRNA. The major disadvantages of using Illumina RNA-Seq reads for this purpose are as follows: i) these reads are single-run reads without resequencing verification; ii) the sequences are short and become even shorter after the poly(A) tail has been removed; iii) these reads are more sensitive to internal priming in A-rich regions, because the first strand of cDNA synthesis uses random hexamers, including hexamer oligo dT, and a very low annealing temperature (25°C for the first strand and 16°C for the second strand synthesis [Illumina Cat # RS-930-1001]); iv) some artifact poly(A) tails are present owing to the fragmentation process of mRNA/cDNA; and v) only a limited number of species with read lengths of 100 bases or more are currently available in the NCBI Sequence Read Archive (SRA) dataset. Therefore, compared with RNA-seq reads, the NCBI mRNA database is presently more reliable for poly(A) site analysis.

Because only a certain percentage of genes have entries in the NCBI mRNA database, the mRNA-mapped poly(A) sites for most species in this study should be viewed as a sample of the total pool of poly(A) sites. Even though we identified 30499 unique poly(A) sites in human (Table 2), some poly(A) sites are likely still not included. This is because we excluded the mRNAs that had any mismatch to the reference genome, in order to avoid ambiguity. It is understood that not all genes in an actual individual have sequences identical to the reference genome; therefore, in the human mRNA analysis, our mapping was likely close to the maximum rate of identifying unique poly(A) sites using a mapping-to-reference-genome approach. Some experimental methods and mapping-to-own-genome approaches should be considered for characterizing additional unique poly(A) sites. The low number of mapped unique poly(A) sites in some species was likely due to the low number of mRNA entries available in the NCBI database. For a number of species, their EST databases were larger than the mRNA ones. For example, in potato, mRNA-based mapping identified only 139 unique poly(A) sites (data not shown), whereas EST-based mapping identified 3182 (data not shown). The NCBI EST database can be valuable for studying the relationship between gene expression profiles and gene expression-related poly(A) site selection. However, we have avoided as much as possible the comparison of mRNA-mapped species with EST-mapped species. This is because we found that some upstream motif frequencies and the A predominance at the poly(A) tail starting position were lower in EST-based mapping than in mRNA-based mapping, suggesting that EST sequences may have been lower in quality than the mRNA sequences were (Xiu-Qing Li, unpublished data). In this mRNA-analysis study, we used ESTs of some species such as the protists *Babesia bovis* and *Blastocystis hominis*, because their poly(A) site patterns were very different from those of plants and animals, and therefore the minor differences between mRNA-based mapping and EST-based mapping would not significantly affect the discussion. The main results of this study represent the surveyed average information for each studied species and characterize the general evolutionary tendency of the poly(A) sites. Further research is required if the goal is to profile all or nearly all the poly(A) sites in a specific species when considerably more sequences are available. Further research is also required to explore the advantage of large numbers of reads and to minimize the disadvantages associated with the issues of low-annealing temperature and DNA fragmentation involved in deep-sequencing-based poly(A) site mapping.

### Species dependence of the most frequent motif

We found that the most frequent hexanucleotide motif in the 50-nucleotide region upstream of the poly(A) tail starting position was different depending on the species: AAUAAA in most species, UGUUUU in the parasite *T. cruzi*, UGUAAC in the alga *C. reinhardtii*, and UAUUUU in three of the four dicotyledonous plant species (barrel medic [*M. truncatula*], poplar [*P. trichocarpa*], and tomato [*S. lycopersicum*]) (Table 2). Unlike in histone mRNA maturation, where endonucleolytic cleavage favours the ACCA motif, although the cleaved ends are likely not polyadenylated [36],[37], in our study ACCA was not the most frequent motif at mRNA poly(A) sites for mRNA in general in any kingdom or species analyzed (Table 2). Since AAUAAA was found to be the most frequent motif in fungi and higher eukaryotes (most plants and animals), it is likely that the use of AAUAAA as a poly(A) signal emerged as early as the common ancestor for fungi, plants, and animals. This interpretation may explain why most of the protists analyzed in this study were found to use motifs other than AAUAAA (Table 2). Further research is required to determine how often protist species do or do not use the AAUAAA motif as a poly(A) signal. The AAUAAA motif was found to be more frequent than any other hexamer in the upstream region in fungi, Arabidopsis, rice, sorghum, and maize, but that motif’s actual frequency in plant mRNA populations was very low compared with its frequency in animal mRNA (Table 2). The results for the study of motifs suggest that some non-animal species did not frequently use AAUAAA as a poly(A) signal and that the most frequent motif for RNA polyadenylation was kingdom-dependent and species-dependent. However, it is interesting that the AAUAAA motif location was highly conserved among fungi, plants, and animals. Further research is required to investigate which groups of genes maintained the use of AAUAAA as the poly(A) signal, as it is known that poly(A) tail lengths were conserved among orthologous mRNAs [38]. Since the predominant motifs are useful factors for predicting poly(A) sites [39], the identification of different predominant motifs or signal motifs in different species and kingdoms in this study is likely very useful for improving poly(A) site prediction in different species and phyla. It is known that *cis*-acting elements in the pre-mRNA surrounding the cleavage site play important roles in pre-mRNA processing and subsequent polyadenylation [40]. Since the identified hexamer motifs were found to be a few hundred times more abundant in the poly(A) site region than in the whole genome on average (this study), the results suggest that the most abundant signature *cis*-motifs (a called *cis*-elements) in the poly(A) site region (Table 2; Figure 8) must play significant roles in pre-mRNA processing or mRNA function during gene expression and therefore may be tentatively called “polyadenylation signal motifs” or “poly(A) signal motifs”.

### U-A-U-A-U base abundance pattern in pre-mRNA and U-A-U-A-A pattern in mature mRNA

We discovered a high degree of conservation of the U-A-U-SiteA-U (or U-A-U-A-U) pattern among different subkingdoms, including at least fungi, plants, and animals. For each species, the base composition obtained in this study was largely in agreement with previous studies in specific species, such as human and mouse [3] and *S. pombe*[5]. Given that the AAUAAA motif was found in only 46.16% of unique mRNAs on average in the 18 AAUAAA-predominant species (calculated from Table 2) and that the on-site UUUA motif was even less frequent, this U-A-U-A-U base abundance pattern may play a greater role in determining the poly(A) site than any known motif alone.

This study not only discovered the high conservation of the U-A-U-A-U base abundance pattern but also determined the exact nucleotide at the peak locations in these patterns and discovered exactly the same nucleotide locations at the same peaks among subkingdoms within the plant kingdom and within the animal kingdom (Table 3; Figure 2). This measurement in different subkingdoms and kingdoms is an advance in precision in terms of characterizing poly(A) signals. In comparison with the findings from previous studies [1],[2],[6],[7],[3],[8],[4],[9],[14], the attributable factors for the high precision of the peak position and the clear U-A-U-A-U base abundance pattern in this study may be the following: i) the use of mRNAs, which are usually verified by repeated sequencing and are therefore higher in quality than ESTs and other single-run-based sequencing reads; ii) the use of all or nearly all the mRNA sequences in the NCBI databases to minimize the bias in sampling sets; iii) the use of unique mRNA and unique poly(A) sites to minimize transposon-caused alterations in the frequencies of mapped poly(A) sites or genes; and iv) the zero-tolerance threshold for mismatches in mRNA–genome mapping.

### RNA structure and signal motif selection

The pre-mRNA U-A-U-A-U base composition pattern (Figure 5), conserved among the fungal, plant, and animal kingdoms may suggest the existence of a relatively conserved RNA folding structure in the poly(A) site region. Further research will be required, particularly on the structural locations of the poly(A) signal motifs and the poly(A) site. An *in vitro* selection assay of human immunodeficiency virus elements suggested that structure, rather than specific sequences, may determine the poly(A) site location [19]. Motifs that are T-rich are frequently found immediately before and after the poly(A) site in some yeast genes [23]. It is known that the poly(A) tail binding protein is also a eukaryotic translation initiation factor, which interacts with protein eIF4G of the mRNA 5′-cap structure [41]. In humans, auxiliary non-AAUAAA elements may aid in polyadenylation efficiency [26]. It is known that many poly(A) sites in human and mouse harbour multiple cleavage sites, leading to heterogeneous 3′-end formation for transcripts [3].

In the present study, the AAUAAA location in relation to the poly(A) site started mainly from the 21th upstream base in human and approximately the 24th upstream base in maize (Figure 8), which means that there were 15 bases between the signal hexamer motif and the poly(A) site in animals. Although further research is required for verification, this highly conserved distance between the poly(A) signal motif and the poly(A) site suggests that in each transcript, the poly(A) site interacted mainly with the closest upstream AAUAAA motif regardless of how many AAUAAA motifs existed in a pre-mRNA.

### Somatic genome variation and poly(A) site region stability

The distance between the major motifs and the poly(A) sites (Figure 8) and between the base frequency peaks (Table 3) was found to be very stable in the pre-mRNAs in this study. The pre-mRNAs and their reference genomes analyzed in the present study were mainly from somatic cells, and more and more evidence suggests that the genome in somatic cells varies during development or in response to the environment [42]–[44]. If the somatic genome varies, how can the poly(A) site patterns from somatic cells be so stable between species and between subkingdoms, such as the nearly identical positions of base abundance peaks in the poly(A) site region between large plant and animal genomes? It is likely that somatic genome variation does not occur randomly and that the poly(A) site region may be a relatively stable part of the somatic genome. Further research is required to investigate the stability differences between the poly(A) site region and other regions on the genome.

### Potential involvement of the U-A-U-A-U pattern in transcription termination

It may be plausible to hypothesize that the U-A-U-A-U base abundance pattern (Figures 5 and 6) likely plays an indirect role in transcription termination, in addition to its major role in polyadenylation. In yeast [45] and human [25] genes, it is known that the segment between the poly(A) site and transcription termination is usually longer than the 101 bases that we analyzed. The U-rich region downstream of the poly(A) site that we analyzed may extend beyond the 101-nucleotide downstream sequences. It is known that mutation in mouse P-globin poly(A) signals displays a markedly reduced efficiency of transcription termination [46]. The U-A-U-A-U pattern may provide an ideal template for testing mutation effects on both transcription termination and poly(A) site selection.

### Base composition at the poly(A) tail attachment position and the poly(A) tail starting position

It is known that base selection is not random for either the poly(A) tail attachment position or the poly(A) tail starting position. The cleavage sites occur usually at a UA or CA dinucleotide in yeast alcohol dehydrogenase genes [47], a CA or UA in *Vitis vinifera* ESTs [48], a CA in simian virus 40 (SV40) [49], and analyzed pooled poly(A) sites from five mammals [4]. In our previous study, for 28 of 29 species or groups of species that included microorganisms, plants, and animals, UA or CA was usually predominant, with U or C at the poly(A) tail attachment position and A at the poly(A) tail starting position [32]. We did not use only orthologous genes in the mRNA database, because the number of genes that were orthologous over such a large spectrum of living organisms (protists, fungi, plants, and animals) was unlikely to be high enough to represent the average poly(A) sites of each species. In the NCBI mRNA database, it is likely that most mRNAs are stable, non-aberrant mRNAs because of the elimination of aberrant mRNAs by the nonsense-mediated mRNA decay pathway [50].

Only *T. cruzi* used predominantly GA at the two positions for the poly(A) site, and in all the previously studied species, the poly(A) tail starting position was mainly an A [32]. In this study, we analyzed more protist species and found that GA was also the most abundant dinucleotide covering both the poly(A) tail attachment position and the poly(A) tail starting position in the unicellular alga *C. reinhardtii* (Table 1). Adenosine did not show any predominance at the poly(A) site in *B. hominis*, and A predominance was not very strong in *C. reinhardtii* either (Figure 1). These results indicate that poly(A) site types in protists are very diverse, whereas the last common ancestor of fungi, plants, animals and some protists likely had A predominance at the poly(A) starting position.

The analysis method differed between this study and our previous study [32] in that this study removed all the redundant poly(A) sites (keeping only one copy per group), whereas the previous study [32] kept all poly(A) site copies on the reference genome, although both studies removed the redundant copies of mRNA before the mapping was conducted. This difference in method means that the present study generated only unique poly(A) sites and that the previous study contained copies from multiple-copy genes. The predominant bases at the poly(A) tail attachment position and the poly(A) tail starting position from these two studies are essentially the same, but maize and human switched from UA predominance [32] to CA predominance (this study), although the CA and UA frequencies were still very similar in this study (Figures 2 and 3). This change in base predominance frequency among mRNAs at the poly(A) tail attachment position suggests that gene amplification or duplication in these two species was done preferentially by genes that had a UA dinucleotide at poly(A) sites (this study).

### Greater stability of the poly(A) region base composition pattern than of gene direction and genomic C + G content

This high evolutionary conservation of base composition pattern in the poly(A) region in fungi, plants, and animals (the present study) was very different from the obvious evolutionary changes in genomic base composition [51] and gene direction on chromosomes [52] in a large number of the living organisms that we analyzed. Among the higher eukaryotes, base composition is extremely poor in C + G content in dicotyledonous plants but extremely rich in C + G content in monocotyledonous plants [51]. Genes on chromosomes in fungi have opposite directions from each other, but there is still a weak predominance of same-direction neighbour genes in most plants and animals [52]. It is known that a higher degree of gene or structural conservation is generally associated with a higher level of importance in function. Proteins that are more critical (identified by high rank positions) in the human signal transduction network are more conserved than other proteins [53]. Similarly, the high conservation of the U-A-U-A-U pattern suggests a high importance in gene functionality.

### Importance of the specific distance between base frequency peaks

The nearly identical positions of averaged base frequency peaks among plant subkingdoms and among animal subkingdoms (Table 3; Figure 4) suggest that both the peak region base composition and the non-peak region length are critical for the function of the poly(A) site region. Just as the third position in a triplet codon must be single and exist even though it wobbles [54], the length of the non-peak region may be critical for proper folding of the structure and for the combination of different subunits of the polyadenylation protein complex. This hypothesis is consistent with the finding that the most abundant motifs were located at a very specific distance from the poly(A) site (Figure 8). The poly(A) complex is known to be composed of some 80 polypeptides [9]. Further research is required to determine the RNA structure of this U-A-U-A-U pattern and to use the pattern/structure to discuss the function of the members in the protein complex responsible for RNA 3′ cleavage and polyadenylation.

### Reasons for the high similarity in the U-A-U-A-U base abundance pattern among subkingdoms

The high similarity of the base abundance patterns in fungi, plants, and animals suggests that the U-A-U-A-U base abundance pattern that we identified is one of the most conserved patterns in higher eukaryote genomes. How is the pattern so highly conserved across at least those three kingdoms? Our hypothesis is that the U-A-U-A-U pattern is highly structured and that a loss of or dramatic change in a component would usually negatively affect the coordinated function of the region. The mutant may often get eliminated during evolution before a new functional balance evolves to benefit from the mutation. An improvement of the complex patterns often requires co-evolutionary improvement of related partners in the pattern. For example, in a co-evolution system previously discovered between a cytoplasmic male-sterility gene and its fertility-restorer gene in the nuclear genome in *Brassica*, the survival of specific alleles of the nuclear fertility-restorer gene in natural populations and modern cultivars is dependent on the existence of its corresponding mitochondrial male-sterility gene in the cytoplasm [55]. In the germplasm lines or cultivars, the nuclear male-fertility-restorer gene *Rfn* co-exists with a mitochondrial male-sterility gene, *orf222*. Similarly, nuclear fertility-restorer gene *Rfp* coexists with mitochondrial male-sterility gene *orf224*[55]. Co-evolution also occurs between the two subunits of ribulose-1,5-bisphosphate carboxylase/oxygenase (RuBisCO) genomes [56].

Another reason for the lack of dramatic change in the U-A-U-A-U pattern in the poly(A) region in fungi, plants, and animals is likely that the system is essential for the performance of an individual and is probably controlled by multiple genes, and that the poly(A) system must maintain functioning during evolution. In another gene system, namely the potato proteinase inhibitor II gene family, evidence was found to indicate that intermediate versions of a proteinase inhibitor II protein domain had to be functional and respond to selection (could not have a pseudogene/dysfunctional-gene stage) during the evolutionary creation of a new predominant type of protein domain from an existing one [57]. Similarly, for this U-A-U-A-U poly(A) site region pattern in fungi, plants, and animals, it is likely that the transient versions during evolution should be functional to support the mutated individual. This “maintenance of functionality” may explain why there were only minor changes between dicotyledonous plants and monocotyledonous plants, between non-mammal animals and mammals, and even among the fungal, plant, and animal kingdoms (this study). The dramatic variation of the poly(A) site base composition pattern in deep-branching, unicellular eukaryotic protists (Figure 1) suggests the following: i) the poly(A) site system was likely controlled by very few genes at the early stage of poly(A) site evolution; and ii) these protists are evolutionarily many times more distant from each other in comparison with the evolutionary distance among the fungal, plant, and animal kingdoms.

Why does a genome make the poly(A) site regions both highly conserved in pattern and poorly conserved in sequence identity? Our hypothesis is that the highly conserved base abundance pattern serves the interaction with the protein complex to ensure RNA cleavage and polyadenylation, and that the purpose of the poor sequence identity between genes in the poly(A) site region (3′UTR and 3′COR) is to avoid genome instability resulting from the recombination of repeated sequences.

### Evolution of base abundance for the poly(A) site region

In this study, signs of evolution can be seen in the U-A-U-A-U base abundance pattern, as follows: i) the A predominance at the poly(A) tail starting position was found to exist in all species analyzed, with the exception of the absence of A preference at the poly(A) site in *B. hominis* and the relative weakness of A preference in *C. reinhardtii*[32]; ii) the overall U predominance in the upstream and downstream regions was detected in fungi and most protists, with the exception of the alga *C. reinhardtii*, in which G was weakly predominant along the two regions (data not shown); iii) the upstream A peak was absent in the human parasite *T. cruzi* but was detected in fungi and the potato late blight pathogen *P. infestans* (Figure 1); iv) the order of the upstream A peak and the U peak in the protist *C. reinhardtii* was the opposite of the order in *P. infestans*, fungi, plants, and animals (Figures 1 and 4); v) there were minor differences in base abundance peaks between plants and animals, but little differences in average-abundance peak locations were found between subkingdoms within the same kingdom (Table 3; Figure 4); and vi) some differences in base composition were detected between subkingdoms (see seqlogo positions 1 to 4 in Figure 4).

## Conclusions

This study determined the sequences and locations of the most predominant motifs [i.e., signature motifs and likely poly(A) signal motifs] in the poly(A) site region, as follows:

i) the UGUAAC motif in *C. reinhardtii*, with a distance of approximately 12 bases between its last C and the poly(A) site;

ii) the UUACUU motif at the poly(A) site in *T. cruzi*;

iii) the weakly abundant UAUUUU motif at the poly(A) site in various dicotyledonous plants;

iv) the UGUUUUGUU motif four bases downstream of the poly(A) site in *B. hominis*;

v) the AAUAAA motif in fungi, plants, and animals. This motif was most predominant even though the actual frequencies were not high in fungi and plants. The location of this motif was found to be highly conserved, with its first A mainly at positions −21 in humans, −22 in Arabidopsis and at position −24 in maize upstream of the poly(A) site; and

vi) the predominance of a specific motif was not found in all the base abundance peak areas, which means that the base composition pattern was also important.

This study identified a highly conserved U-A-U-A-U base abundance pattern that showed only minor variation in terms of peak location among fungi, plants, and animals. The base composition pattern in the poly(A) site region, despite being more conserved than the AAUAAA motif, showed great variation among protists. Compared with higher plants and animals, *B. hominis* and *T. cruzi* were the most different, *C. reinhardtii* did not show U richness in the 3′UTR and 3′COR, and the potato late blight pathogen *P. infestans* was the least different protist among the protists analyzed in the present study. The base composition pattern differences between protist species were many times larger than the differences among the fungal, plant, and animal kingdoms.

These results from this study suggest the following evolutionary process for the poly(A) site: i) the A predominance at the poly(A) site likely emerged earlier than the AAUAAA signal motif; ii) deep-branching unicellular eukaryotes were assayed to have polyadenylation motifs in different locations, including upstream of, at, or downstream of the poly(A) site; iii) the 3′UTR and 3′COR became U-rich with a U > A> > G ≥ C pattern in most species; iv) AAUAAA emerged as a frequent motif in some protists (such as in an ancestor of *P. infestans*) and became the predominant signal motif for polyadenylation in the common ancestor of fungi, plants, and animals; v) the upstream U region with its U-richness peak usually at approximately seven to nine bases from the poly(A) site emerged at approximately the same time or earlier than the AAUAAA motif; vi) the A-rich region at approximately three bases upstream of the poly(A) site emerged in the ancestor shared by fungi and animals but not by plants; and vii) minor differentiation is occurring between subkingdoms within plants and animals in terms of the degree of U and A contents in the 3′UTR and 3′COR.

This study i) provided an actual overview of conserved motifs and base composition patterns surrounding the poly(A) site region in microorganisms, plants, and animals, and ii) determined the approximate stage of the emergence of different components of the base abundance patterns for the poly(A) site region. The peak locations and the distance between the base frequency peaks for the base composition patterns identified in this study can also be used to improve poly(A) site prediction for different organisms in bioinformatics software packages, to investigate the positions and functions of the subunits of the poly(A) complex, and to regulate gene expression in genetic engineering. These discoveries enrich our knowledge on the characteristics and evolution of the poly(A) site.

## Methods

### Nucleotide sequences and species analyzed

The scientific names and common names or descriptions of the species (14 fungi, 5 protists, 5 dicotyledonous plants, 3 monocotyledonous plants, and 17 animals) used in this study are listed in Table 1. Because the potato (*S. tuberosum*) mRNA database was small, we used potato ESTs in mapping. The potato reference genome was a diploid rather than a full cultivated line, but most mRNAs or ESTs were from tetraploid cultivars. To avoid mRNA/EST differences or differences caused by ploidy, we did not quantitatively compare the potato EST-mapped poly(A) sites with the mRNA-mapped poly(A) sites of other species. The nucleotide sequences of completed or at least assembled genomes were downloaded from the NCBI site at http://www.ncbi.nlm.nih.gov/sites/genome, and some sequences were downloaded from http://www.ncbi.nlm.nih.gov/nucleotide/. All the mRNA entries for species corresponding to the analyzed genomes were downloaded from an NCBI nucleotide search (RefSeq, GenBank, EMBL, DDBJ, and PDB databases) at http://www.ncbi.nlm.nih.gov/nucleotide/. Illumina RNA-Seq files for several species were downloaded from the NCBI Sequence Read Archive database (http://www.ncbi.nlm.nih.gov/sra/). A list of the IDs of the genome or chromosomal DNA sequences analyzed can be found in Additional file 1, which includes more species than did the ID list in our previous study [32]. Since individual fungal species do not on their own have sufficient poly(A)-tailed mRNA available in NCBI, we pooled the sequences of fungal species as a fungal group in the analysis, in order to add additional information about these kingdoms. Sequences in the mRNA database are expected to have been verified by resequencing before submission to NCBI and are therefore more reliable than single-run-based sequence reads or ESTs. In the present comparative study, we did not mix the NCBI mRNA sequences with high-throughput sequence reads. We did not conduct a large-scale analysis of deep-sequencing reads, because i) some of the analyzed species did not have high-throughput sequence reads that were publicly available, and ii) most of the available reads were too short [<100 bp after poly(A) tail sequences had been removed] to be analyzed with the same strict thresholds used in this study.

### Poly(A)-tailed mRNA and mapping

Poly(A)-tailed transcript sequences were screened as described in our previous study [32]. In brief, only the transcripts that each had a poly(A) tail of at least 12 continuous A’s at the 3′ end were considered poly(A)-tailed mRNA. The 100 nucleotides immediately upstream of the poly(A) tail starting position were used to screen the mRNA datasets to eliminate any redundant copies. As a result, each sequence in the poly(A)-tailed mRNA dataset was unique. The 100-nucleotide sequence (mainly the 3′UTR) immediately upstream of the poly(A) tail was used to map the mRNAs to their corresponding genomes with zero tolerance for mismatches with the reference genome. The 100 bases of the 3′COR were obtained from the reference genome based on the mapping. The retained 201-nucleotide sequence for each poly(A) site region consisted of the 100 nucleotides of the 3′UTR, the poly(A) tail starting position corresponding to the first A of the poly(A) tail, and the 100 nucleotides downstream of the poly(A) site, also called the 3′COR (Figure 5).

Unlike our recent study [32], this study counted each group of poly(A) sites as one unique site if the 100 nucleotides upstream of the poly(A) sites were identical, regardless of whether the 3′COR was different or not. Therefore, in the present study every poly(A) site region in the final poly(A) site dataset was unique (with redundant copies eliminated). We used the unique mRNAs for genome-level comparisons among organisms by minimizing the overrepresentations (environmental or developmental) related to gene activity. We used the unique poly(A) sites to avoid inflation effects from gene copy number variation, because there is no information on whether all the copies are expressed. We used the poly(A) tail starting position [i.e., the genomic or pre-mRNA nucleotide corresponding to the first A on the poly(A) tail] [32] as a reference (as position 1 in Table 3 and Figure 5, but as position 31 in the seqlogo graphs in Figures 1, 2, 3 and 4, which show 45 bases in the region) for calculating the distance between a base in the region and the poly(A) site. The exact poly(A) site should be the cleaved chemical bond between the poly(A) tail attachment position and the poly(A) tail starting position, as discussed in our previous study [32]. Therefore, in the base composition pattern presentation (Figure 5), we refer to the poly(A) tail attachment position and the poly(A) tail starting position as position −1 and position 1, respectively.

### Motifs and base frequency peaks along the 201-nucleotide region around the poly(A) site

For each unique mRNA, we counted the base composition of each of the 201 nucleotides of the poly(A) site region. For each position of the 201 bases, we searched for the tetranucleotide, pentanucleotide, and hexanucleotide motifs and the base composition abundance at each of the 201 nucleotide positions/locations. When calculating the percentage of mRNAs that had at least one copy of the motif per mRNA in a region, we calculated the unique mRNAs [the unique poly(A) sites that had at least one copy of the motif]. The distance (i.e., base position) of a motif from the poly(A) site was the distance in terms of nucleotide number from the poly(A) tail starting position. A large number of mapped mRNAs in *Pan troglodytes* and *Macaca mulatta*, two primates, were mRNAs predicted by computer software. Therefore, we excluded these two species from further comparative analysis, even though AAUAAA was found to be the most predominant motifs in these species as well.

For the survey of motif frequency (percentage) at each of the 201 locations, we calculated the percentage of mRNAs that had the motif starting from that position. The most abundant motifs, in terms of at least one motif copy per mRNA sequence, for the 50-base region upstream of the poly(A) site were calculated (Table 2). These motifs were used to search for their distribution along the entire 201-base region of the poly(A) site using the motif frequency (mRNA number) at each of the 201 bases. The most abundant upstream motifs in Table 2 and various on-site and downstream motifs were selected to be presented in graphs to show their distribution (Figure 8).

### UTR length

The 3′UTR length in this study means the number of nucleotides between the stop codon and the poly(A) site. The minimal length of the 3′UTR is −1 or −2, or the pre-mRNA is called 3′UTR-less, if an open reading frame (ORF) and the poly(A) tail overlap (such as some mRNA in *B. hominis*). When several ORFs were detected in the same mRNA, only the ORF in the sense direction is considered to be an ORF. The last ORF, if it could potentially encode 110 amino acids or more, was used to calculate the length of the 3′UTR.

### Motif frequency on entire chromosomes

Tetranucleotide and hexanucleotide motifs on entire chromosomes of different species were estimated using DNASTAR Lasergene (www.dnastar.com). However, the poly(A) tail screening, mRNA–genome alignment, and redundant-copy elimination work was assisted by Perl scripts or R programs (http://www.r-project.org/).

### Sequence logo

Sequence logo (seqlogo) graphs were calculated in accordance with Schneider and Stephens [58]. Seqlogo graphs display stacked letters for the nucleotides (i.e., A, C, G, T) at each aligned position, with the size of each letter being proportional to its frequency in the sequences and with the total height of all letters on each position reflecting the information content of the position. We used the seqLogo R package to create all the seqlogos (http://www.bioconductor.org/packages/release/bioc/html/seqLogo.html).

To facilitate the reading and understanding of the seqlogo graphs, we will briefly summarize the calculation and description as follows (readers are referred to the manual for details). Given a position-weight matrix (*p*_*jw*_)_*J*×*W*_ such that each *p*_*jw*_ is the probability of observing nucleotide *j* in position *w* of an alignment of length *W*, where *J* denotes the number of letters in the alphabet from which the sequences were derived, the total height of all letters on each position is decided by the following information content (IC) measure defined as:

(1) $$\mathrm{IC}\left(w\right)=\log_{2}J+\sum_{j=1}^{J}p_{\mathrm{wj}}\;\log_{2}p_{\mathrm{wj}}=\log_{2}J-\mathrm{Entropy}\left(w\right)$$

The information content measured in bits can be shown to be in the range of [0,2] in the case of DNA sequences, with 0 corresponding to the case where all nucleotides occur with equal probability, and 2 corresponding to the case where only a single nucleotide occurs. Therefore, a higher value of information content at a given position indicates higher conservation of nucleotides at that position.

## Competing interests

The authors declare that they have no competing interests.

## Authors’ contributions

XQL conceived and designed the experiments, analyzed the data, and drafted the paper. DD wrote most of the computer scripts/programs. Both authors read, revised, and approved the manuscript.

## Additional files

## Supplementary Material

Additional file 1: List of genome/chromosome/scaffolds that served as reference genomes in this unique polyadenylation site mapping study. Click here for file

Additional file 2: Predominance ranking of the upstream AAUAAA motif among all 4096 hexanucleotide motifs. Click here for file

Additional file 3: Top five most frequent pentanucleotides in each species in the 48-base upstream region. Click here for file

Additional file 4: Frequencies of known downstream motifs. Click here for file

Additional file 5: Most frequent tetramer and hexamer motifs of whole genomes or chromosomes. Click here for file
