# Supplementary material for: Motif types, motif locations and base composition patterns around the RNA polyadenylation site in microorganisms, plants and animals
Source: BMC Evol Biol. 2014 Jul 23;14:162. doi: 10.1186/s12862-014-0162-7 (PMC4360255; doi:10.1186/s12862-014-0162-7)
Supplement: Additional file 1: — List of genome/chromosome/scaffolds that served as reference genomes in this unique polyadenylation site mapping study. [file s12862-014-0162-7-S1.docx]

Li and Du (2014) Motif types, motif locations and base composition patterns around the RNA polyadenylation site in microorganisms, plants and animals. BMC Evol. Biol.

**Additional file 1** List of genome/chromosome/scaffolds that served as reference genomes in this unique polyadenylation site mapping study

| Species Name | | chromosome ID | | |
| --- | --- | --- | --- | --- |
| Anopheles_gambiae | | >NT_078265\| Anopheles gambiae str. PEST chromosome 2L, whole genome shotgun | | |
| Anopheles_gambiae | | >NT_078266\|Anopheles gambiae str. PEST chromosome 2R, whole genome shotgun | | |
| Anopheles_gambiae | | >NT_078267\| Anopheles gambiae str. PEST chromosome 3L, whole genome shotgun | | |
| Anopheles_gambiae | | >NT_078268\| Anopheles gambiae str. PEST chromosome 3R, whole genome shotgun | | |
| Anopheles_gambiae | | >NC_004818\| Anopheles gambiae str. PEST chromosome X, whole genome shotgun | | |
| Anopheles_gambiae | | >NC_002084\| Anopheles gambiae mitochondrion, complete genome. | | |
| Apis_mellifera | | >gi\|110825701\|ref\|NC_007070.2\|NC_007070 Apis mellifera linkage group 1, reference assembly (based on Amel_4.0), whole genome shotgun sequence | | |
| Apis_mellifera | | >gi\|110825711\|ref\|NC_007079.2\|NC_007079 Apis mellifera linkage group 10, reference assembly (based on Amel_4.0), whole genome shotgun sequence | | |
| Apis_mellifera | | >gi\|110825732\|ref\|NC_007080.2\|NC_007080 Apis mellifera linkage group 11, reference assembly (based on Amel_4.0), whole genome shotgun sequence | | |
| Apis_mellifera | | >gi\|110825763\|ref\|NC_007083.2\|NC_007083 Apis mellifera linkage group 14, reference assembly (based on Amel_4.0), whole genome shotgun sequence | | |
| Apis_mellifera | | >gi\|110825764\|ref\|NC_007084.2\|NC_007084 Apis mellifera linkage group 15, reference assembly (based on Amel_4.0), whole genome shotgun sequence | | |
| Apis_mellifera | | >gi\|110825765\|ref\|NC_007085.2\|NC_007085 Apis mellifera linkage group 16, reference assembly (based on Amel_4.0), whole genome shotgun sequence | | |
| Apis_mellifera | | >gi\|110825766\|ref\|NC_007071.2\|NC_007071 Apis mellifera linkage group 2, reference assembly (based on Amel_4.0), whole genome shotgun sequence | | |
| Apis_mellifera | | >gi\|110825767\|ref\|NC_007072.2\|NC_007072 Apis mellifera linkage group 3, reference assembly (based on Amel_4.0), whole genome shotgun sequence | | |
| Apis_mellifera | | >gi\|110825768\|ref\|NC_007073.2\|NC_007073 Apis mellifera linkage group 4, reference assembly (based on Amel_4.0), whole genome shotgun sequence | | |
| Apis_mellifera | | >gi\|110825769\|ref\|NC_007074.2\|NC_007074 Apis mellifera linkage group 5, reference assembly (based on Amel_4.0), whole genome shotgun sequence | | |
| Apis_mellifera | | >gi\|110825770\|ref\|NC_007075.2\|NC_007075 Apis mellifera linkage group 6, reference assembly (based on Amel_4.0), whole genome shotgun sequence | | |
| Apis_mellifera | | >gi\|110825771\|ref\|NC_007076.2\|NC_007076 Apis mellifera linkage group 7, reference assembly (based on Amel_4.0), whole genome shotgun sequence | | |
| Apis_mellifera | | >gi\|110825772\|ref\|NC_007077.2\|NC_007077 Apis mellifera linkage group 8, reference assembly (based on Amel_4.0), whole genome shotgun sequence | | |
| Apis_mellifera | | >gi\|110825774\|ref\|NC_007078.2\|NC_007078 Apis mellifera linkage group 9, reference assembly (based on Amel_4.0), whole genome shotgun sequence | | |
| Arabidopsis_thaliana | | >ref\|NC_003070.9\|:1-30427671 Arabidopsis thaliana chromosome 1, complete sequence | | |
| Arabidopsis_thaliana | | >ref\|NC_003071.7\|:1-19698289 Arabidopsis thaliana chromosome 2, complete sequence | | |
| Arabidopsis_thaliana | | >ref\|NC_003074.8\|:1-23459830 Arabidopsis thaliana chromosome 3, complete sequence | | |
| Arabidopsis_thaliana | | >ref\|NC_003075.7\|:1-18585056 Arabidopsis thaliana chromosome 4, complete sequence | | |
| Arabidopsis_thaliana | | >ref\|NC_003076.8\|:1-26975502 Arabidopsis thaliana chromosome 5, complete sequence | | |
| Aspergillus_nidulans | | >Aspergillus nidulans FGSC A4 scaffold_27, whole genome shotgun | | |
| Aspergillus_nidulans | | >Aspergillus nidulans FGSC A4 chromosome VIII scaffold_16, whole | | |
| Aspergillus_nidulans | | >Aspergillus nidulans FGSC A4 chromosome IV scaffold_15, whole | | |
| Aspergillus_nidulans | | >Aspergillus nidulans FGSC A4 chromosome VII scaffold_14, whole | | |
| Aspergillus_nidulans | | >Aspergillus nidulans FGSC A4 chromosome III scaffold_13, whole | | |
| Aspergillus_nidulans | | >Aspergillus nidulans FGSC A4 scaffold_12, whole genome shotgun | | |
| Aspergillus_nidulans | | >Aspergillus nidulans FGSC A4 chromosome II scaffold_11, whole | | |
| Aspergillus_nidulans | | >Aspergillus nidulans FGSC A4 chromosome IV scaffold_10, whole | | |
| Aspergillus_nidulans | | >Aspergillus nidulans FGSC A4 chromosome IV scaffold_9, whole genome | | |
| Aspergillus_nidulans | | >Aspergillus nidulans FGSC A4 chromosome I scaffold_8, whole genome | | |
| Aspergillus_nidulans | | >Aspergillus nidulans FGSC A4 chromosome I scaffold_7, whole genome | | |
| Aspergillus_nidulans | | >Aspergillus nidulans FGSC A4 chromosome V scaffold_6, whole genome | | |
| Aspergillus_nidulans | | >Aspergillus nidulans FGSC A4 chromosome III scaffold_5, whole | | |
| Aspergillus_nidulans | | >Aspergillus nidulans FGSC A4 chromosome II scaffold_4, whole genome | | |
| Aspergillus_nidulans | | >Aspergillus nidulans FGSC A4 chromosome VI scaffold_3, whole genome | | |
| Aspergillus_nidulans | | >Aspergillus nidulans FGSC A4 chromosome VII scaffold_2, whole | | |
| Aspergillus_nidulans | | >Aspergillus nidulans FGSC A4 chromosome VIII scaffold_1, whole | | |
| Babesia_bovis | | AAXT01.1.fsa_nt.fasta | | |
| Blastocystis hominis | | ASM15166v1 | | |
| Bos_taurus | | >gi\|257481614\|tpg\|GK000001.2\| TPA: Bos taurus chromosome 1, whole genome shotgun sequence | | |
| Bos_taurus | | >gi\|257481335\|tpg\|GK000010.2\| TPA: Bos taurus chromosome 10, whole genome shotgun sequence | | |
| Bos_taurus | | >gi\|257481612\|tpg\|GK000003.2\| TPA: Bos taurus chromosome 3, whole genome shotgun sequence | | |
| Bos_taurus | | >gi\|257481341\|tpg\|GK000004.2\| TPA: Bos taurus chromosome 4, whole genome shotgun sequence | | |
| Bos_taurus | | >gi\|257481340\|tpg\|GK000005.2\| TPA: Bos taurus chromosome 5, whole genome shotgun sequence | | |
| Bos_taurus | | >gi\|257481339\|tpg\|GK000006.2\| TPA: Bos taurus chromosome 6, whole genome shotgun sequence | | |
| Bos_taurus | | >gi\|257481338\|tpg\|GK000007.2\| TPA: Bos taurus chromosome 7, whole genome shotgun sequence | | |
| Bos_taurus | | >gi\|257481337\|tpg\|GK000008.2\| TPA: Bos taurus chromosome 8, whole genome shotgun sequence | | |
| Bos_taurus | | >gi\|257481336\|tpg\|GK000009.2\| TPA: Bos taurus chromosome 9, whole genome shotgun sequence | | |
| Bos_taurus | |  | | |
| Brachypodium_distachyon | | >gi\|289163340\|gb\|CM000880.1\| Brachypodium distachyon strain Bd21 chromosome 1, whole genome shotgun sequence | | |
| Brachypodium_distachyon | | >gi\|289163332\|gb\|GG745432.1\| Brachypodium distachyon strain Bd21 unplaced genomic scaffold BRADIscaffold_10, whole genome shotgun sequence | | |
| Brachypodium_distachyon | | >gi\|289163331\|gb\|GG745433.1\| Brachypodium distachyon strain Bd21 unplaced genomic scaffold BRADIscaffold_11, whole genome shotgun sequence | | |
| Brachypodium_distachyon | | >gi\|289163339\|gb\|CM000881.1\| Brachypodium distachyon strain Bd21 chromosome 2, whole genome shotgun sequence | | |
| Brachypodium_distachyon | | >gi\|289163338\|gb\|CM000882.1\| Brachypodium distachyon strain Bd21 chromosome 3, whole genome shotgun sequence | | |
| Brachypodium_distachyon | | >gi\|289163337\|gb\|CM000883.1\| Brachypodium distachyon strain Bd21 chromosome 4, whole genome shotgun sequence | | |
| Brachypodium_distachyon | | >gi\|289163336\|gb\|CM000884.1\| Brachypodium distachyon strain Bd21 chromosome 5, whole genome shotgun sequence | | |
| Brachypodium_distachyon | | >gi\|289163335\|gb\|GG745429.1\| Brachypodium distachyon strain Bd21 unplaced genomic scaffold BRADIscaffold_7, whole genome shotgun sequence | | |
| Brachypodium_distachyon | | >gi\|289163334\|gb\|GG745430.1\| Brachypodium distachyon strain Bd21 unplaced genomic scaffold BRADIscaffold_8, whole genome shotgun sequence | | |
| Brachypodium_distachyon | | >gi\|289163333\|gb\|GG745431.1\| Brachypodium distachyon strain Bd21 unplaced genomic scaffold BRADIscaffold_9, whole genome shotgun sequence | | |
| Caenorhabditis_elegans | | >gi\|67514031\|emb\|BX284601.3\| Caenorhabditis elegans chromosome I, complete sequence | | |
| Caenorhabditis_elegans | | >gi\|67514032\|emb\|BX284602.3\| Caenorhabditis elegans chromosome II, complete sequence | | |
| Caenorhabditis_elegans | | >gi\|67514029\|emb\|BX284603.2\| Caenorhabditis elegans chromosome III, complete sequence | | |
| Caenorhabditis_elegans | | >gi\|67514030\|emb\|BX284604.2\| Caenorhabditis elegans chromosome IV, complete sequence | | |
| Caenorhabditis_elegans | | >gi\|67514033\|emb\|BX284605.3\| Caenorhabditis elegans chromosome V, complete sequence | | |
| Caenorhabditis_elegans | | >gi\|67424289\|emb\|BX284606.3\| Caenorhabditis elegans chromosome X, complete sequence | | |
| Callithrix_jacchus | | >gi\|290467399\|ref\|NC_013905.1\| Callithrix jacchus chromosome 10, Callithrix jacchus-3.2, whole genome shotgun sequence | | |
| Callithrix_jacchus | | >gi\|290467398\|ref\|NC_013906.1\| Callithrix jacchus chromosome 11, Callithrix jacchus-3.2, whole genome shotgun sequence | | |
| Callithrix_jacchus | | >gi\|290467397\|ref\|NC_013907.1\| Callithrix jacchus chromosome 12, Callithrix jacchus-3.2, whole genome shotgun sequence | | |
| Callithrix_jacchus | | >gi\|290467396\|ref\|NC_013908.1\| Callithrix jacchus chromosome 13, Callithrix jacchus-3.2, whole genome shotgun sequence | | |
| Callithrix_jacchus | | >gi\|290467395\|ref\|NC_013909.1\| Callithrix jacchus chromosome 14, Callithrix jacchus-3.2, whole genome shotgun sequence | | |
| Callithrix_jacchus | | >gi\|290467394\|ref\|NC_013910.1\| Callithrix jacchus chromosome 15, Callithrix jacchus-3.2, whole genome shotgun sequence | | |
| Callithrix_jacchus | | >gi\|290467393\|ref\|NC_013911.1\| Callithrix jacchus chromosome 16, Callithrix jacchus-3.2, whole genome shotgun sequence | | |
| Callithrix_jacchus | | >gi\|290467392\|ref\|NC_013912.1\| Callithrix jacchus chromosome 17, Callithrix jacchus-3.2, whole genome shotgun sequence | | |
| Callithrix_jacchus | | >gi\|290467391\|ref\|NC_013913.1\| Callithrix jacchus chromosome 18, Callithrix jacchus-3.2, whole genome shotgun sequence | | |
| Callithrix_jacchus | | >gi\|290467390\|ref\|NC_013914.1\| Callithrix jacchus chromosome 19, Callithrix jacchus-3.2, whole genome shotgun sequence | | |
| Callithrix_jacchus | | >gi\|290467389\|ref\|NC_013915.1\| Callithrix jacchus chromosome 20, Callithrix jacchus-3.2, whole genome shotgun sequence | | |
| Callithrix_jacchus | | >gi\|290467388\|ref\|NC_013916.1\| Callithrix jacchus chromosome 21, Callithrix jacchus-3.2, whole genome shotgun sequence | | |
| Callithrix_jacchus | | >gi\|290467387\|ref\|NC_013917.1\| Callithrix jacchus chromosome 22, Callithrix jacchus-3.2, whole genome shotgun sequence | | |
| Callithrix_jacchus | | >gi\|290467405\|ref\|NC_013899.1\| Callithrix jacchus chromosome 4, Callithrix jacchus-3.2, whole genome shotgun sequence | | |
| Callithrix_jacchus | | >gi\|290467404\|ref\|NC_013900.1\| Callithrix jacchus chromosome 5, Callithrix jacchus-3.2, whole genome shotgun sequence | | |
| Callithrix_jacchus | | >gi\|290467403\|ref\|NC_013901.1\| Callithrix jacchus chromosome 6, Callithrix jacchus-3.2, whole genome shotgun sequence | | |
| Callithrix_jacchus | | >gi\|290467402\|ref\|NC_013902.1\| Callithrix jacchus chromosome 7, Callithrix jacchus-3.2, whole genome shotgun sequence | | |
| Callithrix_jacchus | | >gi\|290467401\|ref\|NC_013903.1\| Callithrix jacchus chromosome 8, Callithrix jacchus-3.2, whole genome shotgun sequence | | |
| Callithrix_jacchus | | >gi\|290467400\|ref\|NC_013904.1\| Callithrix jacchus chromosome 9, Callithrix jacchus-3.2, whole genome shotgun sequence | | |
| Callithrix_jacchus | | >gi\|290467386\|ref\|NC_013918.1\| Callithrix jacchus chromosome X, Callithrix jacchus-3.2, whole genome shotgun sequence | | |
| Callithrix_jacchus | | >gi\|290467385\|ref\|NC_013919.1\| Callithrix jacchus chromosome Y, Callithrix jacchus-3.2, whole genome shotgun sequence | | |
| Callithrix_jacchus | | >gi\|289734502\|ref\|NW_003187718.1\| Callithrix jacchus chromosome Y unlocalized genomic scaffold, Callithrix jacchus-3.2 CJAY_random_049, whole genome shotgun sequence | | |
| Callithrix_jacchus | | >gi\|289722669\|ref\|NW_003199274.1\| Callithrix jacchus unplaced genomic scaffold, Callithrix jacchus-3.2 CJAUN_random_11556, whole genome shotgun sequence | | |
| Canis_lupus_familiaris | | >gi\|70724219\|gb\|CM000001.2\| Canis familiaris chromosome 1, whole genome shotgun sequence | | |
| Canis_lupus_familiaris | | >gi\|70724210\|gb\|CM000010.2\| Canis familiaris chromosome 10, whole genome shotgun sequence | | |
| Canis_lupus_familiaris | | >gi\|70724218\|gb\|CM000002.2\| Canis familiaris chromosome 2, whole genome shotgun sequence | | |
| Canis_lupus_familiaris | | >gi\|70724216\|gb\|CM000004.2\| Canis familiaris chromosome 4, whole genome shotgun sequence | | |
| Canis_lupus_familiaris | | >gi\|70724215\|gb\|CM000005.2\| Canis familiaris chromosome 5, whole genome shotgun sequence | | |
| Canis_lupus_familiaris | | >gi\|70724214\|gb\|CM000006.2\| Canis familiaris chromosome 6, whole genome shotgun sequence | | |
| Canis_lupus_familiaris | | >gi\|70724213\|gb\|CM000007.2\| Canis familiaris chromosome 7, whole genome shotgun sequence | | |
| Canis_lupus_familiaris | | >gi\|70724212\|gb\|CM000008.2\| Canis familiaris chromosome 8, whole genome shotgun sequence | | |
| Canis_lupus_familiaris | | >gi\|70724211\|gb\|CM000009.2\| Canis familiaris chromosome 9, whole genome shotgun sequence | | |
| Canis_lupus_familiaris | | >gi\|70724181\|gb\|CM000039.2\| Canis familiaris chromosome X, whole genome shotgun sequence | | |
| Ciona_intestinalis | | >gi\|48860548\|ref\|NC_004447.2\| Ciona intestinalis mitochondrion, complete genome | | |
| Ciona_intestinalis | | >gi\|193885917\|ref\|NW_001955232.1\|CinUn_WGA226_1 Ciona intestinalis genomic contig, reference assembly (based on Ciona_intestinalis_v1.0 ciona2276) | | |
| Chlamydomonas_reinhardtii | | All Scafolds downloaded from NCBI on March 6, 2012. | | |
| Danio_rerio | | >gi\|15079186\|ref\|NC_002333.2\| Danio rerio mitochondrion, complete genome | | |
| Danio_rerio | | >gi\|258456220\|ref\|NC_007112.4\| Danio rerio strain Tuebingen chromosome 1, Zv8 | | |
| Danio_rerio | | >gi\|258456211\|ref\|NC_007121.4\| Danio rerio strain Tuebingen chromosome 10, Zv8 | | |
| Danio_rerio | | >gi\|258456210\|ref\|NC_007122.4\| Danio rerio strain Tuebingen chromosome 11, Zv8 | | |
| Danio_rerio | | >gi\|258456209\|ref\|NC_007123.4\| Danio rerio strain Tuebingen chromosome 12, Zv8 | | |
| Danio_rerio | | >gi\|258456208\|ref\|NC_007124.4\| Danio rerio strain Tuebingen chromosome 13, Zv8 | | |
| Danio_rerio | | >gi\|258456207\|ref\|NC_007125.4\| Danio rerio strain Tuebingen chromosome 14, Zv8 | | |
| Danio_rerio | | >gi\|258456206\|ref\|NC_007126.4\| Danio rerio strain Tuebingen chromosome 15, Zv8 | | |
| Danio_rerio | | >gi\|258456205\|ref\|NC_007127.4\| Danio rerio strain Tuebingen chromosome 16, Zv8 | | |
| Danio_rerio | | >gi\|258456204\|ref\|NC_007128.4\| Danio rerio strain Tuebingen chromosome 17, Zv8 | | |
| Danio_rerio | | >gi\|258456203\|ref\|NC_007129.4\| Danio rerio strain Tuebingen chromosome 18, Zv8 | | |
| Danio_rerio | | >gi\|258456202\|ref\|NC_007130.4\| Danio rerio strain Tuebingen chromosome 19, Zv8 | | |
| Danio_rerio | | >gi\|258456219\|ref\|NC_007113.4\| Danio rerio strain Tuebingen chromosome 2, Zv8 | | |
| Danio_rerio | | >gi\|258456201\|ref\|NC_007131.4\| Danio rerio strain Tuebingen chromosome 20, Zv8 | | |
| Danio_rerio | | >gi\|258456200\|ref\|NC_007132.4\| Danio rerio strain Tuebingen chromosome 21, Zv8 | | |
| Danio_rerio | | >gi\|258456199\|ref\|NC_007133.4\| Danio rerio strain Tuebingen chromosome 22, Zv8 | | |
| Danio_rerio | | >gi\|258456198\|ref\|NC_007134.4\| Danio rerio strain Tuebingen chromosome 23, Zv8 | | |
| Danio_rerio | | >gi\|258456197\|ref\|NC_007135.4\| Danio rerio strain Tuebingen chromosome 24, Zv8 | | |
| Danio_rerio | | >gi\|258456196\|ref\|NC_007136.4\| Danio rerio strain Tuebingen chromosome 25, Zv8 | | |
| Danio_rerio | | >gi\|258456218\|ref\|NC_007114.4\| Danio rerio strain Tuebingen chromosome 3, Zv8 | | |
| Danio_rerio | | >gi\|258456217\|ref\|NC_007115.4\| Danio rerio strain Tuebingen chromosome 4, Zv8 | | |
| Danio_rerio | | >gi\|258456216\|ref\|NC_007116.4\| Danio rerio strain Tuebingen chromosome 5, Zv8 | | |
| Danio_rerio | | >gi\|258456215\|ref\|NC_007117.4\| Danio rerio strain Tuebingen chromosome 6, Zv8 | | |
| Danio_rerio | | >gi\|258456214\|ref\|NC_007118.4\| Danio rerio strain Tuebingen chromosome 7, Zv8 | | |
| Danio_rerio | | >gi\|258456213\|ref\|NC_007119.4\| Danio rerio strain Tuebingen chromosome 8, Zv8 | | |
| Danio_rerio | | >gi\|258456212\|ref\|NC_007120.4\| Danio rerio strain Tuebingen chromosome 9, Zv8 | | |
| Danio_rerio | | >gi\|258425119\|ref\|NW_003052456.1\| Danio rerio strain Tuebingen unplaced genomic scaffold, Zv8_NA4098 | | |
| Drosophila_melanogaster | | >ref\|NT_033779.4\|:1-23011544 Drosophila melanogaster chromosome 2L, complete sequence | | |
| Drosophila_melanogaster | | >ref\|NT_033778.3\|:1-21146708 Drosophila melanogaster chromosome 2R, complete sequence | | |
| Drosophila_melanogaster | | >ref\|NT_037436.3\|:1-24543557 Drosophila melanogaster chromosome 3L, complete sequence | | |
| Drosophila_melanogaster | | >ref\|NT_033777.2\|:1-27905053 Drosophila melanogaster chromosome 3R, complete sequence | | |
| Drosophila_melanogaster | | >ref\|NC_004353.3\|:1-1351857 Drosophila melanogaster chromosome 4, complete sequence | | |
| Drosophila_melanogaster | | >ref\|NC_004354.3\|:1-22422827 Drosophila melanogaster chromosome X, complete sequence | | |
| Equus_caballus | | >gi\|194246357\|ref\|NC_009144.2\|NC_009144 Equus caballus chromosome 1, reference assembly (based on EquCab2), whole genome shotgun sequence | | |
| Equus_caballus | | >gi\|194246358\|ref\|NC_009153.2\|NC_009153 Equus caballus chromosome 10, reference assembly (based on EquCab2), whole genome shotgun sequence | | |
| Equus_caballus | | >gi\|194246359\|ref\|NC_009154.2\|NC_009154 Equus caballus chromosome 11, reference assembly (based on EquCab2), whole genome shotgun sequence | | |
| Equus_caballus | | >gi\|194246360\|ref\|NC_009155.2\|NC_009155 Equus caballus chromosome 12, reference assembly (based on EquCab2), whole genome shotgun sequence | | |
| Equus_caballus | | >gi\|194246361\|ref\|NC_009156.2\|NC_009156 Equus caballus chromosome 13, reference assembly (based on EquCab2), whole genome shotgun sequence | | |
| Equus_caballus | | >gi\|194246362\|ref\|NC_009157.2\|NC_009157 Equus caballus chromosome 14, reference assembly (based on EquCab2), whole genome shotgun sequence | | |
| Equus_caballus | | >gi\|194246363\|ref\|NC_009158.2\|NC_009158 Equus caballus chromosome 15, reference assembly (based on EquCab2), whole genome shotgun sequence | | |
| Equus_caballus | | >gi\|194246364\|ref\|NC_009159.2\|NC_009159 Equus caballus chromosome 16, reference assembly (based on EquCab2), whole genome shotgun sequence | | |
| Equus_caballus | | >gi\|194246365\|ref\|NC_009160.2\|NC_009160 Equus caballus chromosome 17, reference assembly (based on EquCab2), whole genome shotgun sequence | | |
| Equus_caballus | | >gi\|194246366\|ref\|NC_009161.2\|NC_009161 Equus caballus chromosome 18, reference assembly (based on EquCab2), whole genome shotgun sequence | | |
| Equus_caballus | | >gi\|194246370\|ref\|NC_009162.2\|NC_009162 Equus caballus chromosome 19, reference assembly (based on EquCab2), whole genome shotgun sequence | | |
| Equus_caballus | | >gi\|194246371\|ref\|NC_009145.2\|NC_009145 Equus caballus chromosome 2, reference assembly (based on EquCab2), whole genome shotgun sequence | | |
| Equus_caballus | | >gi\|194246372\|ref\|NC_009163.2\|NC_009163 Equus caballus chromosome 20, reference assembly (based on EquCab2), whole genome shotgun sequence | | |
| Equus_caballus | | >gi\|194246373\|ref\|NC_009164.2\|NC_009164 Equus caballus chromosome 21, reference assembly (based on EquCab2), whole genome shotgun sequence | | |
| Equus_caballus | | >gi\|194246374\|ref\|NC_009165.2\|NC_009165 Equus caballus chromosome 22, reference assembly (based on EquCab2), whole genome shotgun sequence | | |
| Equus_caballus | | >gi\|194246375\|ref\|NC_009166.2\|NC_009166 Equus caballus chromosome 23, reference assembly (based on EquCab2), whole genome shotgun sequence | | |
| Equus_caballus | | >gi\|194246376\|ref\|NC_009167.2\|NC_009167 Equus caballus chromosome 24, reference assembly (based on EquCab2), whole genome shotgun sequence | | |
| Equus_caballus | | >gi\|194246377\|ref\|NC_009168.2\|NC_009168 Equus caballus chromosome 25, reference assembly (based on EquCab2), whole genome shotgun sequence | | |
| Equus_caballus | | >gi\|194246378\|ref\|NC_009169.2\|NC_009169 Equus caballus chromosome 26, reference assembly (based on EquCab2), whole genome shotgun sequence | | |
| Equus_caballus | | >gi\|194246379\|ref\|NC_009170.2\|NC_009170 Equus caballus chromosome 27, reference assembly (based on EquCab2), whole genome shotgun sequence | | |
| Equus_caballus | | >gi\|194246380\|ref\|NC_009171.2\|NC_009171 Equus caballus chromosome 28, reference assembly (based on EquCab2), whole genome shotgun sequence | | |
| Equus_caballus | | >gi\|194246381\|ref\|NC_009172.2\|NC_009172 Equus caballus chromosome 29, reference assembly (based on EquCab2), whole genome shotgun sequence | | |
| Equus_caballus | | >gi\|194246382\|ref\|NC_009146.2\|NC_009146 Equus caballus chromosome 3, reference assembly (based on EquCab2), whole genome shotgun sequence | | |
| Equus_caballus | | >gi\|194246383\|ref\|NC_009173.2\|NC_009173 Equus caballus chromosome 30, reference assembly (based on EquCab2), whole genome shotgun sequence | | |
| Equus_caballus | | >gi\|194246384\|ref\|NC_009174.2\|NC_009174 Equus caballus chromosome 31, reference assembly (based on EquCab2), whole genome shotgun sequence | | |
| Equus_caballus | | >gi\|194246385\|ref\|NC_009147.2\|NC_009147 Equus caballus chromosome 4, reference assembly (based on EquCab2), whole genome shotgun sequence | | |
| Equus_caballus | | >gi\|194246386\|ref\|NC_009148.2\|NC_009148 Equus caballus chromosome 5, reference assembly (based on EquCab2), whole genome shotgun sequence | | |
| Equus_caballus | | >gi\|194246387\|ref\|NC_009149.2\|NC_009149 Equus caballus chromosome 6, reference assembly (based on EquCab2), whole genome shotgun sequence | | |
| Equus_caballus | | >gi\|194246388\|ref\|NC_009150.2\|NC_009150 Equus caballus chromosome 7, reference assembly (based on EquCab2), whole genome shotgun sequence | | |
| Equus_caballus | | >gi\|194246389\|ref\|NC_009151.2\|NC_009151 Equus caballus chromosome 8, reference assembly (based on EquCab2), whole genome shotgun sequence | | |
| Equus_caballus | | >gi\|194246401\|ref\|NC_009152.2\|NC_009152 Equus caballus chromosome 9, reference assembly (based on EquCab2), whole genome shotgun sequence | | |
| Equus_caballus | | >gi\|194246402\|ref\|NC_009175.2\|NC_009175 Equus caballus chromosome X, reference assembly (based on EquCab2), whole genome shotgun sequence | | |
| Felis_catus | | >gi\|224998174\|gb\|CM000695.1\| Felis catus breed mixed chromosome A1, whole genome shotgun sequence | | |
| Felis_catus | | >gi\|224998172\|gb\|CM000697.1\| Felis catus breed mixed chromosome A3, whole genome shotgun sequence | | |
| Felis_catus | | >gi\|224998171\|gb\|CM000698.1\| Felis catus breed mixed chromosome B1, whole genome shotgun sequence | | |
| Felis_catus | | >gi\|224998170\|gb\|CM000699.1\| Felis catus breed mixed chromosome B2, whole genome shotgun sequence | | |
| Felis_catus | | >gi\|224998169\|gb\|CM000700.1\| Felis catus breed mixed chromosome B3, whole genome shotgun sequence | | |
| Felis_catus | | >gi\|224998168\|gb\|CM000701.1\| Felis catus breed mixed chromosome B4, whole genome shotgun sequence | | |
| Felis_catus | | >gi\|224998167\|gb\|CM000702.1\| Felis catus breed mixed chromosome C1, whole genome shotgun sequence | | |
| Felis_catus | | >gi\|224998166\|gb\|CM000703.1\| Felis catus breed mixed chromosome C2, whole genome shotgun sequence | | |
| Felis_catus | | >gi\|224998161\|gb\|CM000708.1\| Felis catus breed mixed chromosome E1, whole genome shotgun sequence | | |
| Felis_catus | | >gi\|224998158\|gb\|CM000711.1\| Felis catus breed mixed chromosome F1, whole genome shotgun sequence | | |
| Felis_catus | | >gi\|224998156\|gb\|CM000713.1\| Felis catus breed mixed chromosome X, whole genome shotgun sequence | | |
| Fungi_Neurospora_crassa_uid132 | | >gi\|194294104\|gb\|EU815636.1\| Neurospora crass chromosome LGVII translocation T(AR173) breakpoint sequence | | |
| Fungi_Phytophthora_infestans | | >gi\|48249219\|gb\|AY561505.1\| Phytophthora infestans transposon hAT-like transposable element DodoPi-5, partial sequence | | |
| Gallus_gallus | | >gi\|117306184\|gb\|CM000093.2\| Gallus gallus chromosome 1, whole genome shotgun sequence | | |
| Gallus_gallus | | >gi\|117306152\|gb\|CM000102.2\| Gallus gallus chromosome 10, whole genome shotgun sequence | | |
| Gallus_gallus | | >gi\|117306183\|gb\|CM000094.2\| Gallus gallus chromosome 2, whole genome shotgun sequence | | |
| Gallus_gallus | | >gi\|117306159\|gb\|CM000095.2\| Gallus gallus chromosome 3, whole genome shotgun sequence | | |
| Gallus_gallus | | >gi\|117306158\|gb\|CM000096.2\| Gallus gallus chromosome 4, whole genome shotgun sequence | | |
| Gallus_gallus | | >gi\|117306157\|gb\|CM000097.2\| Gallus gallus chromosome 5, whole genome shotgun sequence | | |
| Gallus_gallus | | >gi\|117306156\|gb\|CM000098.2\| Gallus gallus chromosome 6, whole genome shotgun sequence | | |
| Gallus_gallus | | >gi\|117306155\|gb\|CM000099.2\| Gallus gallus chromosome 7, whole genome shotgun sequence | | |
| Gallus_gallus | | >gi\|117306154\|gb\|CM000100.2\| Gallus gallus chromosome 8, whole genome shotgun sequence | | |
| Gallus_gallus | | >gi\|117306153\|gb\|CM000101.2\| Gallus gallus chromosome 9, whole genome shotgun sequence | | |
| Gallus_gallus | | >gi\|117306007\|gb\|CM000121.2\| Gallus gallus chromosome W, whole genome shotgun sequence | | |
| Gallus_gallus | | >gi\|117306003\|gb\|CM000122.2\| Gallus gallus chromosome Z, whole genome shotgun sequence | | |
| Homo__sapiens | | >ref\|NT_008705.16\|:1-39094935 Homo sapiens chromosome 10 genomic contig, GRCh37 reference primary assembly | | |
| Homo__sapiens | | >ref\|NT_033985.7\|:1-4072029 Homo sapiens chromosome 10 genomic contig, GRCh37 reference primary assembly | | |
| Homo__sapiens | | >ref\|NT_031847.8\|:1-952205 Homo sapiens chromosome 10 genomic contig, GRCh37 reference primary assembly | | |
| Homo__sapiens | | >ref\|NT_077570.1\|:1-263307 Homo sapiens chromosome 10 genomic contig, GRCh37 reference primary assembly | | |
| Homo__sapiens | | >ref\|NT_077571.1\|:1-163231 Homo sapiens chromosome 10 genomic contig, GRCh37 reference primary assembly | | |
| Homo__sapiens | | >ref\|NT_030772.10\|:1-989829 Homo sapiens chromosome 10 genomic contig, GRCh37 reference primary assembly | | |
| Homo__sapiens | | >ref\|NT_030059.13\|:1-79420533 Homo sapiens chromosome 10 genomic contig, GRCh37 reference primary assembly | | |
| Homo__sapiens | | >ref\|NT_008818.16\|:1-6758678 Homo sapiens chromosome 10 genomic contig, GRCh37 reference primary assembly | | |
| Homo__sapiens | | >ref\|NT_009237.18\|:1-50723853 Homo sapiens chromosome 11 genomic contig, GRCh37 reference primary assembly | | |
| Homo__sapiens | | >ref\|NT_035158.2\|:1-503352 Homo sapiens chromosome 11 genomic contig, GRCh37 reference primary assembly | | |
| Homo__sapiens | | >ref\|NT_167190.1\|:1-41593379 Homo sapiens chromosome 11 genomic contig, GRCh37 reference primary assembly | | |
| Homo__sapiens | | >ref\|NT_033899.8\|:1-38508932 Homo sapiens chromosome 11 genomic contig, GRCh37 reference primary assembly | | |
| Homo__sapiens | | >ref\|NT_009759.16\|:1-7129876 Homo sapiens chromosome 12 genomic contig, GRCh37 reference primary assembly | | |
| Homo__sapiens | | >ref\|NT_009714.17\|:1-27616818 Homo sapiens chromosome 12 genomic contig, GRCh37 reference primary assembly | | |
| Homo__sapiens | | >ref\|NT_029419.12\|:1-71516776 Homo sapiens chromosome 12 genomic contig, GRCh37 reference primary assembly | | |
| Homo__sapiens | | >ref\|NT_009775.17\|:1-13107153 Homo sapiens chromosome 12 genomic contig, GRCh37 reference primary assembly | | |
| Homo__sapiens | | >ref\|NT_009755.19\|:1-10126369 Homo sapiens chromosome 12 genomic contig, GRCh37 reference primary assembly | | |
| Homo__sapiens | | >ref\|NT_024477.14\|:1-1034903 Homo sapiens chromosome 12 genomic contig, GRCh37 reference primary assembly | | |
| Homo__sapiens | | >ref\|NT_024524.14\|:1-67740324 Homo sapiens chromosome 13 genomic contig, GRCh37 reference primary assembly | | |
| Homo__sapiens | | >ref\|NT_009952.14\|:1-25443670 Homo sapiens chromosome 13 genomic contig, GRCh37 reference primary assembly | | |
| Homo__sapiens | | >ref\|NT_027140.6\|:1-1821999 Homo sapiens chromosome 13 genomic contig, GRCh37 reference primary assembly | | |
| Homo__sapiens | | >ref\|NT_077627.3\|:1-213955 Homo sapiens chromosome 13 genomic contig, GRCh37 reference primary assembly | | |
| Homo__sapiens | | >ref\|NT_024498.12\|:1-369930 Homo sapiens chromosome 13 genomic contig, GRCh37 reference primary assembly | | |
| Homo__sapiens | | >ref\|NT_026437.12\|:1-88289540 Homo sapiens chromosome 14 genomic contig, GRCh37 reference primary assembly | | |
| Homo__sapiens | | >ref\|NT_037852.6\|:1-2212114 Homo sapiens chromosome 15 genomic contig, GRCh37 reference primary assembly | | |
| Homo__sapiens | | >ref\|NT_077631.1\|:1-334079 Homo sapiens chromosome 15 genomic contig, GRCh37 reference primary assembly | | |
| Homo__sapiens | | >ref\|NT_078094.2\|:1-868660 Homo sapiens chromosome 15 genomic contig, GRCh37 reference primary assembly | | |
| Homo__sapiens | | >ref\|NT_026446.14\|:1-5594590 Homo sapiens chromosome 15 genomic contig, GRCh37 reference primary assembly | | |
| Homo__sapiens | | >ref\|NT_010194.17\|:1-53620202 Homo sapiens chromosome 15 genomic contig, GRCh37 reference primary assembly | | |
| Homo__sapiens | | >ref\|NT_077661.3\|:1-2104828 Homo sapiens chromosome 15 genomic contig, GRCh37 reference primary assembly | | |
| Homo__sapiens | | >ref\|NT_010274.17\|:1-17486919 Homo sapiens chromosome 15 genomic contig, GRCh37 reference primary assembly | | |
| Homo__sapiens | | >ref\|NT_010393.16\|:1-33963150 Homo sapiens chromosome 16 genomic contig, GRCh37 reference primary assembly | | |
| Homo__sapiens | | >ref\|NT_024773.11\|:1-1112651 Homo sapiens chromosome 16 genomic contig, GRCh37 reference primary assembly | | |
| Homo__sapiens | | >ref\|NT_010498.15\|:1-42003582 Homo sapiens chromosome 16 genomic contig, GRCh37 reference primary assembly | | |
| Homo__sapiens | | >ref\|NT_010542.15\|:1-1855370 Homo sapiens chromosome 16 genomic contig, GRCh37 reference primary assembly | | |
| Homo__sapiens | | >ref\|NT_024972.8\|:1-296626 Homo sapiens chromosome 17 genomic contig, GRCh37 reference primary assembly | | |
| Homo__sapiens | | >ref\|NT_010718.16\|:1-21169982 Homo sapiens chromosome 17 genomic contig, GRCh37 reference primary assembly | | |
| Homo__sapiens | | >ref\|NT_024862.14\|:1-596398 Homo sapiens chromosome 17 genomic contig, GRCh37 reference primary assembly | | |
| Homo__sapiens | | >ref\|NT_010799.15\|:1-9412842 Homo sapiens chromosome 17 genomic contig, GRCh37 reference primary assembly | | |
| Homo__sapiens | | >ref\|NT_010783.15\|:1-44983201 Homo sapiens chromosome 17 genomic contig, GRCh37 reference primary assembly | | |
| Homo__sapiens | | >ref\|NT_010663.15\|:1-1436161 Homo sapiens chromosome 17 genomic contig, GRCh37 reference primary assembly | | |
| Homo__sapiens | | >ref\|NT_010859.14\|:1-15400898 Homo sapiens chromosome 18 genomic contig, GRCh37 reference primary assembly | | |
| Homo__sapiens | | >ref\|NT_010966.14\|:1-33548238 Homo sapiens chromosome 18 genomic contig, GRCh37 reference primary assembly | | |
| Homo__sapiens | | >ref\|NT_025028.14\|:1-25808112 Homo sapiens chromosome 18 genomic contig, GRCh37 reference primary assembly | | |
| Homo__sapiens | | >ref\|NT_011255.14\|:1-7286004 Homo sapiens chromosome 19 genomic contig, GRCh37 reference primary assembly | | |
| Homo__sapiens | | >ref\|NT_077812.2\|:1-1291194 Homo sapiens chromosome 19 genomic contig, GRCh37 reference primary assembly | | |
| Homo__sapiens | | >ref\|NT_011295.11\|:1-15894584 Homo sapiens chromosome 19 genomic contig, GRCh37 reference primary assembly | | |
| Homo__sapiens | | >ref\|NT_011109.16\|:1-31387201 Homo sapiens chromosome 19 genomic contig, GRCh37 reference primary assembly | | |
| Homo__sapiens | | >ref\|NT_077402.2\|:1-257719 Homo sapiens chromosome 1 genomic contig, GRCh37 reference primary assembly | | |
| Homo__sapiens | | >ref\|NT_113797.1\|:1-126477 Homo sapiens chromosome 1 genomic contig, GRCh37 reference primary assembly | | |
| Homo__sapiens | | >ref\|NT_079485.4\|:1-224781 Homo sapiens chromosome 1 genomic contig, GRCh37 reference primary assembly | | |
| Homo__sapiens | | >ref\|NT_079497.3\|:1-78698 Homo sapiens chromosome 1 genomic contig, GRCh37 reference primary assembly | | |
| Homo__sapiens | | >ref\|NT_077933.2\|:1-347932 Homo sapiens chromosome 1 genomic contig, GRCh37 reference primary assembly | | |
| Homo__sapiens | | >ref\|NT_167185.1\|:1-3353625 Homo sapiens chromosome 1 genomic contig, GRCh37 reference primary assembly | | |
| Homo__sapiens | | >ref\|NT_113799.1\|:1-185320 Homo sapiens chromosome 1 genomic contig, GRCh37 reference primary assembly | | |
| Homo__sapiens | | >ref\|NT_078067.3\|:1-376183 Homo sapiens chromosome 9 genomic contig, GRCh37 reference primary assembly | | |
| Homo__sapiens | | >ref\|NT_086602.2\|:1-259514 Homo sapiens chromosome 1 genomic contig, GRCh37 reference primary assembly | | |
| Homo__sapiens | | >ref\|NT_167186.1\|:1-42425989 Homo sapiens chromosome 1 genomic contig, GRCh37 reference primary assembly | | |
| Homo__sapiens | | >ref\|NT_032968.8\|:1-182411 Homo sapiens chromosome 1 genomic contig, GRCh37 reference primary assembly | | |
| Homo__sapiens | | >ref\|NT_077912.1\|:1-153649 Homo sapiens chromosome 1 genomic contig, GRCh37 reference primary assembly | | |
| Homo__sapiens | | >ref\|NT_004350.19\|:1-3323900 Homo sapiens chromosome 1 genomic contig, GRCh37 reference primary assembly | | |
| Homo__sapiens | | >ref\|NT_021937.19\|:1-9224644 Homo sapiens chromosome 1 genomic contig, GRCh37 reference primary assembly | | |
| Homo__sapiens | | >ref\|NT_004610.19\|:1-16558170 Homo sapiens chromosome 1 genomic contig, GRCh37 reference primary assembly | | |
| Homo__sapiens | | >ref\|NT_032977.9\|:1-90908613 Homo sapiens chromosome 1 genomic contig, GRCh37 reference primary assembly | | |
| Homo__sapiens | | >ref\|NT_077389.3\|:1-398739 Homo sapiens chromosome 1 genomic contig, GRCh37 reference primary assembly | | |
| Homo__sapiens | | >ref\|NT_113793.2\|:1-432327 Homo sapiens chromosome 1 genomic contig, GRCh37 reference primary assembly | | |
| Homo__sapiens | | >ref\|NT_113796.2\|:1-426764 Homo sapiens chromosome 1 genomic contig, GRCh37 reference primary assembly | | |
| Homo__sapiens | | >ref\|NT_011387.8\|:1-26259569 Homo sapiens chromosome 20 genomic contig, GRCh37 reference primary assembly | | |
| Homo__sapiens | | >ref\|NT_025215.4\|:1-234339 Homo sapiens chromosome 20 genomic contig, GRCh37 reference primary assembly | | |
| Homo__sapiens | | >ref\|NT_011362.10\|:1-31409461 Homo sapiens chromosome 20 genomic contig, GRCh37 reference primary assembly | | |
| Homo__sapiens | | >ref\|NT_011333.6\|:1-1702151 Homo sapiens chromosome 20 genomic contig, GRCh37 reference primary assembly | | |
| Homo__sapiens | | >ref\|NT_113952.1\|:1-184355 Homo sapiens chromosome 21 genomic contig, GRCh37 reference primary assembly | | |
| Homo__sapiens | | >ref\|NT_113954.1\|:1-129889 Homo sapiens chromosome 21 genomic contig, GRCh37 reference primary assembly | | |
| Homo__sapiens | | >ref\|NT_113958.2\|:1-209483 Homo sapiens chromosome 21 genomic contig, GRCh37 reference primary assembly | | |
| Homo__sapiens | | >ref\|NT_113953.1\|:1-131056 Homo sapiens chromosome 21 genomic contig, GRCh37 reference primary assembly | | |
| Homo__sapiens | | >ref\|NT_113955.2\|:1-281920 Homo sapiens chromosome 21 genomic contig, GRCh37 reference primary assembly | | |
| Homo__sapiens | | >ref\|NT_029490.4\|:1-490233 Homo sapiens chromosome 21 genomic contig, GRCh37 reference primary assembly | | |
| Homo__sapiens | | >ref\|NT_011512.11\|:1-28617430 Homo sapiens chromosome 21 genomic contig, GRCh37 reference primary assembly | | |
| Homo__sapiens | | >ref\|NT_011515.12\|:1-5114336 Homo sapiens chromosome 21 genomic contig, GRCh37 reference primary assembly | | |
| Homo__sapiens | | >ref\|NT_028395.3\|:1-647850 Homo sapiens chromosome 22 genomic contig, GRCh37 reference primary assembly | | |
| Homo__sapiens | | >ref\|NT_011519.10\|:1-3661581 Homo sapiens chromosome 22 genomic contig, GRCh37 reference primary assembly | | |
| Homo__sapiens | | >ref\|NT_011520.12\|:1-29755346 Homo sapiens chromosome 22 genomic contig, GRCh37 reference primary assembly | | |
| Homo__sapiens | | >ref\|NT_011526.7\|:1-829789 Homo sapiens chromosome 22 genomic contig, GRCh37 reference primary assembly | | |
| Homo__sapiens | | >ref\|NT_022221.13\|:1-3519312 Homo sapiens chromosome 2 genomic contig, GRCh37 reference primary assembly | | |
| Homo__sapiens | | >ref\|NT_005403.17\|:1-84213159 Homo sapiens chromosome 2 genomic contig, GRCh37 reference primary assembly | | |
| Homo__sapiens | | >ref\|NT_005120.16\|:1-5748237 Homo sapiens chromosome 2 genomic contig, GRCh37 reference primary assembly | | |
| Homo__sapiens | | >ref\|NT_022173.11\|:1-952154 Homo sapiens chromosome 2 genomic contig, GRCh37 reference primary assembly | | |
| Homo__sapiens | | >ref\|NT_005416.13\|:1-2380241 Homo sapiens chromosome 2 genomic contig, GRCh37 reference primary assembly | | |
| Homo__sapiens | | >ref\|NT_022139.13\|:1-1439476 Homo sapiens chromosome 2 genomic contig, GRCh37 reference primary assembly | | |
| Homo__sapiens | | >ref\|NT_005334.16\|:1-11160936 Homo sapiens chromosome 2 genomic contig, GRCh37 reference primary assembly | | |
| Homo__sapiens | | >ref\|NT_015926.15\|:1-4823389 Homo sapiens chromosome 2 genomic contig, GRCh37 reference primary assembly | | |
| Homo__sapiens | | >ref\|NT_022184.15\|:1-68452323 Homo sapiens chromosome 2 genomic contig, GRCh37 reference primary assembly | | |
| Homo__sapiens | | >ref\|NT_032994.6\|:1-714667 Homo sapiens chromosome 2 genomic contig, GRCh37 reference primary assembly | | |
| Homo__sapiens | | >ref\|NT_034508.2\|:1-731068 Homo sapiens chromosome 2 genomic contig, GRCh37 reference primary assembly | | |
| Homo__sapiens | | >ref\|NT_022171.15\|:1-14783166 Homo sapiens chromosome 2 genomic contig, GRCh37 reference primary assembly | | |
| Homo__sapiens | | >ref\|NT_022135.16\|:1-39439245 Homo sapiens chromosome 2 genomic contig, GRCh37 reference primary assembly | | |
| Homo__sapiens | | >ref\|NT_022517.18\|:1-66110270 Homo sapiens chromosome 3 genomic contig, GRCh37 reference primary assembly | | |
| Homo__sapiens | | >ref\|NT_022459.15\|:1-24234584 Homo sapiens chromosome 3 genomic contig, GRCh37 reference primary assembly | | |
| Homo__sapiens | | >ref\|NT_005612.16\|:1-100537107 Homo sapiens chromosome 3 genomic contig, GRCh37 reference primary assembly | | |
| Homo__sapiens | | >ref\|NT_029928.13\|:1-3915179 Homo sapiens chromosome 3 genomic contig, GRCh37 reference primary assembly | | |
| Homo__sapiens | | >ref\|NT_037622.5\|:1-1413146 Homo sapiens chromosome 4 genomic contig, GRCh37 reference primary assembly | | |
| Homo__sapiens | | >ref\|NT_016354.19\|:1-115591997 Homo sapiens chromosome 4 genomic contig, GRCh37 reference primary assembly | | |
| Homo__sapiens | | >ref\|NT_006051.18\|:1-7320557 Homo sapiens chromosome 4 genomic contig, GRCh37 reference primary assembly | | |
| Homo__sapiens | | >ref\|NT_006316.16\|:1-23002714 Homo sapiens chromosome 4 genomic contig, GRCh37 reference primary assembly | | |
| Homo__sapiens | | >ref\|NT_022794.10\|:1-997221 Homo sapiens chromosome 4 genomic contig, GRCh37 reference primary assembly | | |
| Homo__sapiens | | >ref\|NT_016297.16\|:1-7455758 Homo sapiens chromosome 4 genomic contig, GRCh37 reference primary assembly | | |
| Homo__sapiens | | >ref\|NT_006238.11\|:1-9041845 Homo sapiens chromosome 4 genomic contig, GRCh37 reference primary assembly | | |
| Homo__sapiens | | >ref\|NT_037645.2\|:1-171176 Homo sapiens chromosome 4 genomic contig, GRCh37 reference primary assembly | | |
| Homo__sapiens | | >ref\|NT_022853.15\|:1-7079216 Homo sapiens chromosome 4 genomic contig, GRCh37 reference primary assembly | | |
| Homo__sapiens | | >ref\|NT_022778.16\|:1-15638046 Homo sapiens chromosome 4 genomic contig, GRCh37 reference primary assembly | | |
| Homo__sapiens | | >ref\|NT_006576.16\|:1-46395641 Homo sapiens chromosome 5 genomic contig, GRCh37 reference primary assembly | | |
| Homo__sapiens | | >ref\|NT_006713.15\|:1-42230487 Homo sapiens chromosome 5 genomic contig, GRCh37 reference primary assembly | | |
| Homo__sapiens | | >ref\|NT_034772.6\|:1-47100945 Homo sapiens chromosome 5 genomic contig, GRCh37 reference primary assembly | | |
| Homo__sapiens | | >ref\|NT_029289.11\|:1-16301654 Homo sapiens chromosome 5 genomic contig, GRCh37 reference primary assembly | | |
| Homo__sapiens | | >ref\|NT_023133.13\|:1-25716533 Homo sapiens chromosome 5 genomic contig, GRCh37 reference primary assembly | | |
| Homo__sapiens | | >ref\|NT_007592.15\|:1-58720166 Homo sapiens chromosome 6 genomic contig, GRCh37 reference primary assembly | | |
| Homo__sapiens | | >ref\|NT_007299.13\|:1-33800377 Homo sapiens chromosome 6 genomic contig, GRCh37 reference primary assembly | | |
| Homo__sapiens | | >ref\|NT_025741.15\|:1-75224524 Homo sapiens chromosome 6 genomic contig, GRCh37 reference primary assembly | | |
| Homo__sapiens | | >ref\|NT_007819.17\|:1-50360631 Homo sapiens chromosome 7 genomic contig, GRCh37 reference primary assembly | | |
| Homo__sapiens | | >ref\|NT_033968.6\|:1-7643700 Homo sapiens chromosome 7 genomic contig, GRCh37 reference primary assembly | | |
| Homo__sapiens | | >ref\|NT_023603.5\|:1-256182 Homo sapiens chromosome 7 genomic contig, GRCh37 reference primary assembly | | |
| Homo__sapiens | | >ref\|NT_077528.2\|:1-556644 Homo sapiens chromosome 7 genomic contig, GRCh37 reference primary assembly | | |
| Homo__sapiens | | >ref\|NT_007933.15\|:1-77412220 Homo sapiens chromosome 7 genomic contig, GRCh37 reference primary assembly | | |
| Homo__sapiens | | >ref\|NT_007914.15\|:1-14866257 Homo sapiens chromosome 7 genomic contig, GRCh37 reference primary assembly | | |
| Homo__sapiens | | >ref\|NT_007741.14\|:1-4758029 Homo sapiens chromosome 7 genomic contig, GRCh37 reference primary assembly | | |
| Homo__sapiens | | >ref\|NT_023736.17\|:1-7464649 Homo sapiens chromosome 8 genomic contig, GRCh37 reference primary assembly | | |
| Homo__sapiens | | >ref\|NT_077531.4\|:1-4567205 Homo sapiens chromosome 8 genomic contig, GRCh37 reference primary assembly | | |
| Homo__sapiens | | >ref\|NT_167187.1\|:1-31697033 Homo sapiens chromosome 8 genomic contig, GRCh37 reference primary assembly | | |
| Homo__sapiens | | >ref\|NT_023678.16\|:1-1291612 Homo sapiens chromosome 8 genomic contig, GRCh37 reference primary assembly | | |
| Homo__sapiens | | >ref\|NT_008183.19\|:1-38440852 Homo sapiens chromosome 8 genomic contig, GRCh37 reference primary assembly | | |
| Homo__sapiens | | >ref\|NT_008046.16\|:1-58606137 Homo sapiens chromosome 8 genomic contig, GRCh37 reference primary assembly | | |
| Homo__sapiens | | >ref\|NT_037704.5\|:1-871434 Homo sapiens chromosome 8 genomic contig, GRCh37 reference primary assembly | | |
| Homo__sapiens | | >ref\|NT_008413.18\|:1-39964796 Homo sapiens chromosome 9 genomic contig, GRCh37 reference primary assembly | | |
| Homo__sapiens | | >ref\|NT_086755.3\|:1-499094 Homo sapiens chromosome 9 genomic contig, GRCh37 reference primary assembly | | |
| Homo__sapiens | | >ref\|NT_078078.3\|:1-157546 Homo sapiens chromosome 9 genomic contig, GRCh37 reference primary assembly | | |
| Homo__sapiens | | >ref\|NT_078052.4\|:1-450681 Homo sapiens chromosome 9 genomic contig, GRCh37 reference primary assembly | | |
| Homo__sapiens | | >ref\|NT_167189.1\|:1-1139474 Homo sapiens chromosome 9 genomic contig, GRCh37 reference primary assembly | | |
| Homo__sapiens | | >ref\|NT_079533.1\|:1-158462 Homo sapiens chromosome 9 genomic contig, GRCh37 reference primary assembly | | |
| Homo__sapiens | | >ref\|NT_078066.4\|:1-471702 Homo sapiens chromosome 9 genomic contig, GRCh37 reference primary assembly | | |
| Homo__sapiens | | >ref\|NT_078067.3\|:1-376183 Homo sapiens chromosome 9 genomic contig, GRCh37 reference primary assembly | | |
| Homo__sapiens | | >ref\|NT_078068.1\|:1-174765 Homo sapiens chromosome 9 genomic contig, GRCh37 reference primary assembly | | |
| Homo__sapiens | | >ref\|NT_078070.3\|:1-1229783 Homo sapiens chromosome 9 genomic contig, GRCh37 reference primary assembly | | |
| Homo__sapiens | | >ref\|NT_113816.1\|:1-187806 Homo sapiens chromosome 9 genomic contig, GRCh37 reference primary assembly | | |
| Homo__sapiens | | >ref\|NT_078049.4\|:1-401038 Homo sapiens chromosome 9 genomic contig, GRCh37 reference primary assembly | | |
| Homo__sapiens | | >ref\|NT_113817.1\|:1-178933 Homo sapiens chromosome 9 genomic contig, GRCh37 reference primary assembly | | |
| Homo__sapiens | | >ref\|NT_008470.19\|:1-62237592 Homo sapiens chromosome 9 genomic contig, GRCh37 reference primary assembly | | |
| Homo__sapiens | | >ref\|NT_035014.4\|:1-3818133 Homo sapiens chromosome 9 genomic contig, GRCh37 reference primary assembly | | |
| Homo__sapiens | | >ref\|NT_019501.13\|:1-2075804 Homo sapiens chromosome 9 genomic contig, GRCh37 reference primary assembly | | |
| Homo__sapiens | | >ref\|NT_024000.16\|:1-1936434 Homo sapiens chromosome 9 genomic contig, GRCh37 reference primary assembly | | |
| Homo__sapiens | | >ref\|NT_078041.2\|:1-464507 Homo sapiens chromosome 9 genomic contig, GRCh37 reference primary assembly | | |
| Homo__sapiens | | >ref\|NT_113812.2\|:1-375452 Homo sapiens chromosome 9 genomic contig, GRCh37 reference primary assembly | | |
| Homo__sapiens | | >ref\|NT_078043.5\|:1-1198162 Homo sapiens chromosome 9 genomic contig, GRCh37 reference primary assembly | | |
| Homo__sapiens | | >ref\|NT_078055.4\|:1-549743 Homo sapiens chromosome 9 genomic contig, GRCh37 reference primary assembly | | |
| Homo__sapiens | | >ref\|NT_167188.1\|:1-1936505 Homo sapiens chromosome 9 genomic contig, GRCh37 reference primary assembly | | |
| Homo__sapiens | | >ref\|NT_078053.4\|:1-465318 Homo sapiens chromosome 9 genomic contig, GRCh37 reference primary assembly | | |
| Homo__sapiens | | >ref\|NT_113813.2\|:1-595518 Homo sapiens chromosome 9 genomic contig, GRCh37 reference primary assembly | | |
| Homo__sapiens | | >ref\|NT_167191.1\|:1-34821 Homo sapiens chromosome X genomic contig, GRCh37 reference primary assembly | | |
| Homo__sapiens | | >ref\|NT_011638.13\|:1-2371741 Homo sapiens chromosome X genomic contig, GRCh37 reference primary assembly | | |
| Homo__sapiens | | >ref\|NT_011630.14\|:1-6136098 Homo sapiens chromosome X genomic contig, GRCh37 reference primary assembly | | |
| Homo__sapiens | | >ref\|NT_011669.17\|:1-14971680 Homo sapiens chromosome X genomic contig, GRCh37 reference primary assembly | | |
| Homo__sapiens | | >ref\|NT_011651.17\|:1-36813976 Homo sapiens chromosome X genomic contig, GRCh37 reference primary assembly | | |
| Homo__sapiens | | >ref\|NT_028405.12\|:1-2114622 Homo sapiens chromosome X genomic contig, GRCh37 reference primary assembly | | |
| Homo__sapiens | | >ref\|NT_011786.16\|:1-27775034 Homo sapiens chromosome X genomic contig, GRCh37 reference primary assembly | | |
| Homo__sapiens | | >ref\|NT_011681.16\|:1-5474738 Homo sapiens chromosome X genomic contig, GRCh37 reference primary assembly | | |
| Homo__sapiens | | >ref\|NT_167198.1\|:1-6178498 Homo sapiens chromosome X genomic contig, GRCh37 reference primary assembly | | |
| Homo__sapiens | | >ref\|NT_167192.1\|:1-86563 Homo sapiens chromosome X genomic contig, GRCh37 reference primary assembly | | |
| Homo__sapiens | | >ref\|NT_167193.1\|:1-766173 Homo sapiens chromosome X genomic contig, GRCh37 reference primary assembly | | |
| Homo__sapiens | | >ref\|NT_167194.1\|:1-36556 Homo sapiens chromosome X genomic contig, GRCh37 reference primary assembly | | |
| Homo__sapiens | | >ref\|NT_167195.1\|:1-80121 Homo sapiens chromosome X genomic contig, GRCh37 reference primary assembly | | |
| Homo__sapiens | | >ref\|NT_167196.1\|:1-754004 Homo sapiens chromosome X genomic contig, GRCh37 reference primary assembly | | |
| Homo__sapiens | | >ref\|NT_167197.1\|:1-34980018 Homo sapiens chromosome X genomic contig, GRCh37 reference primary assembly | | |
| Homo__sapiens | | >ref\|NT_079573.4\|:1-12094741 Homo sapiens chromosome X genomic contig, GRCh37 reference primary assembly | | |
| Homo__sapiens | | >ref\|NT_086939.3\|:1-681176 Homo sapiens chromosome X genomic contig, GRCh37 reference primary assembly | | |
| Homo__sapiens | | >ref\|NT_167199.1\|:1-34821 Homo sapiens chromosome Y genomic contig, GRCh37 reference primary assembly | | |
| Homo__sapiens | | >ref\|NT_011878.9\|:1-813231 Homo sapiens chromosome Y genomic contig, GRCh37 reference primary assembly | | |
| Homo__sapiens | | >ref\|NT_087001.1\|:1-39401 Homo sapiens chromosome Y genomic contig, GRCh37 reference primary assembly | | |
| Homo__sapiens | | >ref\|NT_113819.1\|:1-554624 Homo sapiens chromosome Y genomic contig, GRCh37 reference primary assembly | | |
| Homo__sapiens | | >ref\|NT_011875.12\|:1-10102850 Homo sapiens chromosome Y genomic contig, GRCh37 reference primary assembly | | |
| Homo__sapiens | | >ref\|NT_011903.12\|:1-4867933 Homo sapiens chromosome Y genomic contig, GRCh37 reference primary assembly | | |
| Homo__sapiens | | >ref\|NT_025975.2\|:1-98295 Homo sapiens chromosome Y genomic contig, GRCh37 reference primary assembly | | |
| Homo__sapiens | | >ref\|NT_091573.1\|:1-66393 Homo sapiens chromosome Y genomic contig, GRCh37 reference primary assembly | | |
| Homo__sapiens | | >ref\|NT_167206.1\|:1-329517 Homo sapiens chromosome Y genomic contig, GRCh37 reference primary assembly | | |
| Homo__sapiens | | >ref\|NT_167200.1\|:1-86563 Homo sapiens chromosome Y genomic contig, GRCh37 reference primary assembly | | |
| Homo__sapiens | | >ref\|NT_167201.1\|:1-766173 Homo sapiens chromosome Y genomic contig, GRCh37 reference primary assembly | | |
| Homo__sapiens | | >ref\|NT_167202.1\|:1-36556 Homo sapiens chromosome Y genomic contig, GRCh37 reference primary assembly | | |
| Homo__sapiens | | >ref\|NT_167203.1\|:1-80121 Homo sapiens chromosome Y genomic contig, GRCh37 reference primary assembly | | |
| Homo__sapiens | | >ref\|NT_167204.1\|:1-754004 Homo sapiens chromosome Y genomic contig, GRCh37 reference primary assembly | | |
| Homo__sapiens | | >ref\|NT_167205.1\|:1-581282 Homo sapiens chromosome Y genomic contig, GRCh37 reference primary assembly | | |
| Homo__sapiens | | >ref\|NT_011896.9\|:1-6265435 Homo sapiens chromosome Y genomic contig, GRCh37 reference primary assembly | | |
| Homo__sapiens | | >ref\|NT_086998.1\|:1-276367 Homo sapiens chromosome Y genomic contig, GRCh37 reference primary assembly | | |
| Macaca_mulatta | | >gi\|49146236\|ref\|NC_005943.1\| Macaca mulatta mitochondrion, complete genome | | |
| Macaca_mulatta | | >gi\|109156578\|ref\|NC_007858.1\| Macaca mulatta chromosome 1, Mmul_051212 chromosome, whole genome shotgun sequence | | |
| Macaca_mulatta | | >gi\|109156579\|ref\|NC_007867.1\| Macaca mulatta chromosome 10, Mmul_051212 chromosome, whole genome shotgun sequence | | |
| Macaca_mulatta | | >gi\|109156580\|ref\|NC_007868.1\| Macaca mulatta chromosome 11, Mmul_051212 chromosome, whole genome shotgun sequence | | |
| Macaca_mulatta | | >gi\|109156645\|ref\|NC_007869.1\| Macaca mulatta chromosome 12, Mmul_051212 chromosome, whole genome shotgun sequence | | |
| Macaca_mulatta | | >gi\|109156646\|ref\|NC_007870.1\| Macaca mulatta chromosome 13, Mmul_051212 chromosome, whole genome shotgun sequence | | |
| Macaca_mulatta | | >gi\|109156648\|ref\|NC_007871.1\| Macaca mulatta chromosome 14, Mmul_051212 chromosome, whole genome shotgun sequence | | |
| Macaca_mulatta | | >gi\|109156649\|ref\|NC_007872.1\| Macaca mulatta chromosome 15, Mmul_051212 chromosome, whole genome shotgun sequence | | |
| Macaca_mulatta | | >gi\|109156650\|ref\|NC_007873.1\| Macaca mulatta chromosome 16, Mmul_051212 chromosome, whole genome shotgun sequence | | |
| Macaca_mulatta | | >gi\|109156884\|ref\|NC_007874.1\| Macaca mulatta chromosome 17, Mmul_051212 chromosome, whole genome shotgun sequence | | |
| Macaca_mulatta | | >gi\|109156885\|ref\|NC_007875.1\| Macaca mulatta chromosome 18, Mmul_051212 chromosome, whole genome shotgun sequence | | |
| Macaca_mulatta | | >gi\|109156886\|ref\|NC_007876.1\| Macaca mulatta chromosome 19, Mmul_051212 chromosome, whole genome shotgun sequence | | |
| Macaca_mulatta | | >gi\|109156887\|ref\|NC_007859.1\| Macaca mulatta chromosome 2, Mmul_051212 chromosome, whole genome shotgun sequence | | |
| Macaca_mulatta | | >gi\|109156888\|ref\|NC_007877.1\| Macaca mulatta chromosome 20, Mmul_051212 chromosome, whole genome shotgun sequence | | |
| Macaca_mulatta | | >gi\|109156890\|ref\|NC_007860.1\| Macaca mulatta chromosome 3, Mmul_051212 chromosome, whole genome shotgun sequence | | |
| Macaca_mulatta | | >gi\|109156893\|ref\|NC_007861.1\| Macaca mulatta chromosome 4, Mmul_051212 chromosome, whole genome shotgun sequence | | |
| Macaca_mulatta | | >gi\|109156895\|ref\|NC_007862.1\| Macaca mulatta chromosome 5, Mmul_051212 chromosome, whole genome shotgun sequence | | |
| Macaca_mulatta | | >gi\|109157119\|ref\|NC_007863.1\| Macaca mulatta chromosome 6, Mmul_051212 chromosome, whole genome shotgun sequence | | |
| Macaca_mulatta | | >gi\|109158192\|ref\|NC_007864.1\| Macaca mulatta chromosome 7, Mmul_051212 chromosome, whole genome shotgun sequence | | |
| Macaca_mulatta | | >gi\|109158193\|ref\|NC_007865.1\| Macaca mulatta chromosome 8, Mmul_051212 chromosome, whole genome shotgun sequence | | |
| Macaca_mulatta | | >gi\|109158194\|ref\|NC_007866.1\| Macaca mulatta chromosome 9, Mmul_051212 chromosome, whole genome shotgun sequence | | |
| Macaca_mulatta | | >gi\|109158195\|ref\|NC_007878.1\| Macaca mulatta chromosome X, Mmul_051212 chromosome, whole genome shotgun sequence | | |
| Macaca_mulatta | | >gi\|90704326\|ref\|NW_001219154.1\| Macaca mulatta chromosome X unlocalized genomic scaffold, Mmul_051212, whole genome shotgun sequence | | |
| Macaca_mulatta | | >gi\|90780474\|ref\|NW_001211473.1\| Macaca mulatta unplaced genomic scaffold, Mmul_051212, whole genome shotgun sequence | | |
| \| \| Medicago_truncatula \| >gi\|357521745\|ref\|NC_016407.1\| Medicago truncatula chromosome 1 \| \| --- \| --- \| \| Medicago_truncatula \| >gi\|357521756\|ref\|NC_016408.1\| Medicago truncatula chromosome 2 \| \| Medicago_truncatula \| >gi\|357521765\|ref\|NC_016409.1\| Medicago truncatula chromosome 3 \| \| Medicago_truncatula \| >gi\|357521768\|ref\|NC_016410.1\| Medicago truncatula chromosome 4 \| \| Medicago_truncatula \| >gi\|357521778\|ref\|NC_016411.1\| Medicago truncatula chromosome 5 \| \| Medicago_truncatula \| >gi\|357521773\|ref\|NC_016412.1\| Medicago truncatula chromosome 6 \| \| Medicago_truncatula \| >gi\|357521775\|ref\|NC_016413.1\| Medicago truncatula chromosome 7 \| \| Medicago_truncatula_Chrs \| >gi\|357521776\|ref\|NC_016414.1\| Medicago truncatula chromosome 8 \| \| \| \| --- \| --- \| --- \| --- \| --- \| --- \| --- \| --- \| --- \| --- \| --- \| --- \| --- \| --- \| --- \| --- \| --- \| --- \| | | \| \| Medicago_truncatula \| >gi\|357521745\|ref\|NC_016407.1\| Medicago truncatula chromosome 1 \| \| --- \| --- \| \| Medicago_truncatula \| >gi\|357521756\|ref\|NC_016408.1\| Medicago truncatula chromosome 2 \| \| Medicago_truncatula \| >gi\|357521765\|ref\|NC_016409.1\| Medicago truncatula chromosome 3 \| \| Medicago_truncatula \| >gi\|357521768\|ref\|NC_016410.1\| Medicago truncatula chromosome 4 \| \| Medicago_truncatula \| >gi\|357521778\|ref\|NC_016411.1\| Medicago truncatula chromosome 5 \| \| Medicago_truncatula \| >gi\|357521773\|ref\|NC_016412.1\| Medicago truncatula chromosome 6 \| \| Medicago_truncatula \| >gi\|357521775\|ref\|NC_016413.1\| Medicago truncatula chromosome 7 \| \| Medicago_truncatula_Chrs \| >gi\|357521776\|ref\|NC_016414.1\| Medicago truncatula chromosome 8 \| \| \| \| --- \| --- \| --- \| --- \| --- \| --- \| --- \| --- \| --- \| --- \| --- \| --- \| --- \| --- \| --- \| --- \| --- \| --- \| | | |
| Monodelphis_domestica | | >gi\|126361933\|ref\|NC_008801.1\|NC_008801 Monodelphis domestica chromosome 1, reference assembly (based on MonDom5), whole genome shotgun sequence | | |
| Monodelphis_domestica | | >gi\|126362075\|ref\|NC_008802.1\|NC_008802 Monodelphis domestica chromosome 2, reference assembly (based on MonDom5), whole genome shotgun sequence | | |
| Monodelphis_domestica | | >gi\|126362809\|ref\|NC_008803.1\|NC_008803 Monodelphis domestica chromosome 3, reference assembly (based on MonDom5), whole genome shotgun sequence | | |
| Monodelphis_domestica | | >gi\|126362810\|ref\|NC_008804.1\|NC_008804 Monodelphis domestica chromosome 4, reference assembly (based on MonDom5), whole genome shotgun sequence | | |
| Monodelphis_domestica | | >gi\|126362941\|ref\|NC_008805.1\|NC_008805 Monodelphis domestica chromosome 5, reference assembly (based on MonDom5), whole genome shotgun sequence | | |
| Monodelphis_domestica | | >gi\|126362942\|ref\|NC_008806.1\|NC_008806 Monodelphis domestica chromosome 6, reference assembly (based on MonDom5), whole genome shotgun sequence | | |
| Monodelphis_domestica | | >gi\|126362943\|ref\|NC_008807.1\|NC_008807 Monodelphis domestica chromosome 7, reference assembly (based on MonDom5), whole genome shotgun sequence | | |
| Monodelphis_domestica | | >gi\|126362944\|ref\|NC_008808.1\|NC_008808 Monodelphis domestica chromosome 8, reference assembly (based on MonDom5), whole genome shotgun sequence | | |
| Monodelphis_domestica | | >gi\|126362945\|ref\|NC_008809.1\|NC_008809 Monodelphis domestica chromosome X, reference assembly (based on MonDom5), whole genome shotgun sequence | | |
| Mus_musculus | | >ref\|NT_039169.7\|Mm1_39209_37:1-19423349 Mus musculus chromosome 1 genomic contig, strain C57BL/6J | | |
| Mus_musculus | | >ref\|NT_039170.7\|Mm1_39210_37:1-52628781 Mus musculus chromosome 1 genomic contig, strain C57BL/6J | | |
| Mus_musculus | | >ref\|NT_039173.7\|Mm1_39213_37:1-10225548 Mus musculus chromosome 1 genomic contig, strain C57BL/6J | | |
| Mus_musculus | | >ref\|NT_078297.6\|Mm1_78362_37:1-70622195 Mus musculus chromosome 1 genomic contig, strain C57BL/6J | | |
| Mus_musculus | | >ref\|NT_039185.7\|Mm1_39225_37:1-26822937 Mus musculus chromosome 1 genomic contig, strain C57BL/6J | | |
| Mus_musculus | | >ref\|NT_039189.7\|Mm1_39229_37:1-8497847 Mus musculus chromosome 1 genomic contig, strain C57BL/6J | | |
| Mus_musculus | | >ref\|NT_039190.7\|Mm1_39230_37:1-3319775 Mus musculus chromosome 1 genomic contig, strain C57BL/6J | | |
| Mus_musculus | | >ref\|NT_039202.7\|Mm2_39242_37:1-19347252 Mus musculus chromosome 2 genomic contig, strain C57BL/6J | | |
| Mus_musculus | | >ref\|NT_039206.7\|Mm2_39246_37:1-36511446 Mus musculus chromosome 2 genomic contig, strain C57BL/6J | | |
| Mus_musculus | | >ref\|NT_039207.7\|Mm2_39247_37:1-116366104 Mus musculus chromosome 2 genomic contig, strain C57BL/6J | | |
| Mus_musculus | | >ref\|NT_166284.1\|Mm2_163458_37:1-617724 Mus musculus chromosome 2 genomic contig, strain C57BL/6J | | |
| Mus_musculus | | >ref\|NT_078355.6\|Mm2_78420_37:1-1356266 Mus musculus chromosome 2 genomic contig, strain C57BL/6J | | |
| Mus_musculus | | >ref\|NT_039212.6\|Mm2_39252_37:1-4299295 Mus musculus chromosome 2 genomic contig, strain C57BL/6J | | |
| Mus_musculus | | >ref\|NT_078380.6\|Mm3_78445_37:1-12610690 Mus musculus chromosome 3 genomic contig, strain C57BL/6J | | |
| Mus_musculus | | >ref\|NT_162143.3\|Mm3_159849_37:1-24788849 Mus musculus chromosome 3 genomic contig, strain C57BL/6J | | |
| Mus_musculus | | >ref\|NT_039229.7\|Mm3_39269_37:1-10113108 Mus musculus chromosome 3 genomic contig, strain C57BL/6J | | |
| Mus_musculus | | >ref\|NT_039240.7\|Mm3_39280_37:1-80458789 Mus musculus chromosome 3 genomic contig, strain C57BL/6J | | |
| Mus_musculus | | >ref\|NT_166285.1\|Mm3_163459_37:1-28473347 Mus musculus chromosome 3 genomic contig, strain C57BL/6J | | |
| Mus_musculus | | >gi\|74229896\|gb\|CM000224.2\| Mus musculus chromosome 16, whole genome shotgun sequence | | |
| Mus_musculus | | >gi\|74229895\|gb\|CM000225.2\| Mus musculus chromosome 17, whole genome shotgun sequence | | |
| Mus_musculus | | >gi\|74229894\|gb\|CM000226.2\| Mus musculus chromosome 18, whole genome shotgun sequence | | |
| Mus_musculus | | >gi\|71913465\|gb\|CM000227.1\| Mus musculus chromosome 19, whole genome shotgun sequence | | |
| Mus_musculus | | >gi\|74229906\|gb\|CM000214.2\| Mus musculus chromosome 6, whole genome shotgun sequence | | |
| Mus_musculus | | >gi\|74229905\|gb\|CM000215.2\| Mus musculus chromosome 7, whole genome shotgun sequence | | |
| Mus_musculus | | >gi\|74229893\|gb\|CM000228.2\| Mus musculus chromosome X, whole genome shotgun sequence | | |
| Mus_musculus | | >gi\|71913463\|gb\|CM000229.1\| Mus musculus chromosome Y, whole genome shotgun sequence | | |
| Neurospora crassa | | gi\|164429762\|ref\|NW_001849831.1\| , etc. 210 contigs, whole genome shotgun sequence | | |
| Ornithorhynchus_anatinus | | >gi\|149712631\|ref\|NC_009094.1\|NC_009094 Ornithorhynchus anatinus chromosome 1, reference assembly (based on Ornithorhynchus_anatinus-5.0.1), whole genome shotgun sequence | | |
| Ornithorhynchus_anatinus | | >gi\|149714148\|ref\|NC_009103.1\|NC_009103 Ornithorhynchus anatinus chromosome 10, reference assembly (based on Ornithorhynchus_anatinus-5.0.1), whole genome shotgun sequence | | |
| Ornithorhynchus_anatinus | | >gi\|149715145\|ref\|NC_009104.1\|NC_009104 Ornithorhynchus anatinus chromosome 11, reference assembly (based on Ornithorhynchus_anatinus-5.0.1), whole genome shotgun sequence | | |
| Ornithorhynchus_anatinus | | >gi\|149716377\|ref\|NC_009105.1\|NC_009105 Ornithorhynchus anatinus chromosome 12, reference assembly (based on Ornithorhynchus_anatinus-5.0.1), whole genome shotgun sequence | | |
| Ornithorhynchus_anatinus | | >gi\|149716935\|ref\|NC_009107.1\|NC_009107 Ornithorhynchus anatinus chromosome 14, reference assembly (based on Ornithorhynchus_anatinus-5.0.1), whole genome shotgun sequence | | |
| Ornithorhynchus_anatinus | | >gi\|149717714\|ref\|NC_009108.1\|NC_009108 Ornithorhynchus anatinus chromosome 15, reference assembly (based on Ornithorhynchus_anatinus-5.0.1), whole genome shotgun sequence | | |
| Ornithorhynchus_anatinus | | >gi\|149717939\|ref\|NC_009110.1\|NC_009110 Ornithorhynchus anatinus chromosome 17, reference assembly (based on Ornithorhynchus_anatinus-5.0.1), whole genome shotgun sequence | | |
| Ornithorhynchus_anatinus | | >gi\|149719225\|ref\|NC_009111.1\|NC_009111 Ornithorhynchus anatinus chromosome 18, reference assembly (based on Ornithorhynchus_anatinus-5.0.1), whole genome shotgun sequence | | |
| Ornithorhynchus_anatinus | | >gi\|149721824\|ref\|NC_009095.1\|NC_009095 Ornithorhynchus anatinus chromosome 2, reference assembly (based on Ornithorhynchus_anatinus-5.0.1), whole genome shotgun sequence | | |
| Ornithorhynchus_anatinus | | >gi\|149722085\|ref\|NC_009112.1\|NC_009112 Ornithorhynchus anatinus chromosome 20, reference assembly (based on Ornithorhynchus_anatinus-5.0.1), whole genome shotgun sequence | | |
| Ornithorhynchus_anatinus | | >gi\|149725021\|ref\|NC_009096.1\|NC_009096 Ornithorhynchus anatinus chromosome 3, reference assembly (based on Ornithorhynchus_anatinus-5.0.1), whole genome shotgun sequence | | |
| Ornithorhynchus_anatinus | | >gi\|149727112\|ref\|NC_009097.1\|NC_009097 Ornithorhynchus anatinus chromosome 4, reference assembly (based on Ornithorhynchus_anatinus-5.0.1), whole genome shotgun sequence | | |
| Ornithorhynchus_anatinus | | >gi\|149728214\|ref\|NC_009098.1\|NC_009098 Ornithorhynchus anatinus chromosome 5, reference assembly (based on Ornithorhynchus_anatinus-5.0.1), whole genome shotgun sequence | | |
| Ornithorhynchus_anatinus | | >gi\|149729612\|ref\|NC_009099.1\|NC_009099 Ornithorhynchus anatinus chromosome 6, reference assembly (based on Ornithorhynchus_anatinus-5.0.1), whole genome shotgun sequence | | |
| Ornithorhynchus_anatinus | | >gi\|149731469\|ref\|NC_009100.1\|NC_009100 Ornithorhynchus anatinus chromosome 7, reference assembly (based on Ornithorhynchus_anatinus-5.0.1), whole genome shotgun sequence | | |
| Ornithorhynchus_anatinus | | >gi\|149737093\|ref\|NC_009114.1\|NC_009114 Ornithorhynchus anatinus chromosome X1, reference assembly (based on Ornithorhynchus_anatinus-5.0.1), whole genome shotgun sequence | | |
| Ornithorhynchus_anatinus | | >gi\|149737330\|ref\|NC_009115.1\|NC_009115 Ornithorhynchus anatinus chromosome X2, reference assembly (based on Ornithorhynchus_anatinus-5.0.1), whole genome shotgun sequence | | |
| Ornithorhynchus_anatinus | | >gi\|149737646\|ref\|NC_009116.1\|NC_009116 Ornithorhynchus anatinus chromosome X3, reference assembly (based on Ornithorhynchus_anatinus-5.0.1), whole genome shotgun sequence | | |
| Ornithorhynchus_anatinus | | >gi\|149742078\|ref\|NC_009118.1\|NC_009118 Ornithorhynchus anatinus chromosome X5, reference assembly (based on Ornithorhynchus_anatinus-5.0.1), whole genome shotgun sequence | | |
| Oryctolagus_cuniculus | | >gi\|5835526\|ref\|NC_001913.1\| Oryctolagus cuniculus mitochondrion, complete genome | | |
| Oryctolagus_cuniculus | | >gi\|283562148\|ref\|NC_013669.1\| Oryctolagus cuniculus breed Thorbecke inbred chromosome 1, OryCun2.0, whole genome shotgun sequence | | |
| Oryctolagus_cuniculus | | >gi\|283562139\|ref\|NC_013678.1\| Oryctolagus cuniculus breed Thorbecke inbred chromosome 10, OryCun2.0, whole genome shotgun sequence | | |
| Oryctolagus_cuniculus | | >gi\|283562138\|ref\|NC_013679.1\| Oryctolagus cuniculus breed Thorbecke inbred chromosome 11, OryCun2.0, whole genome shotgun sequence | | |
| Oryctolagus_cuniculus | | >gi\|283562137\|ref\|NC_013680.1\| Oryctolagus cuniculus breed Thorbecke inbred chromosome 12, OryCun2.0, whole genome shotgun sequence | | |
| Oryctolagus_cuniculus | | >gi\|283562136\|ref\|NC_013681.1\| Oryctolagus cuniculus breed Thorbecke inbred chromosome 13, OryCun2.0, whole genome shotgun sequence | | |
| Oryctolagus_cuniculus | | >gi\|283562135\|ref\|NC_013682.1\| Oryctolagus cuniculus breed Thorbecke inbred chromosome 14, OryCun2.0, whole genome shotgun sequence | | |
| Oryctolagus_cuniculus | | >gi\|283562134\|ref\|NC_013683.1\| Oryctolagus cuniculus breed Thorbecke inbred chromosome 15, OryCun2.0, whole genome shotgun sequence | | |
| Oryctolagus_cuniculus | | >gi\|283562133\|ref\|NC_013684.1\| Oryctolagus cuniculus breed Thorbecke inbred chromosome 16, OryCun2.0, whole genome shotgun sequence | | |
| Oryctolagus_cuniculus | | >gi\|283562132\|ref\|NC_013685.1\| Oryctolagus cuniculus breed Thorbecke inbred chromosome 17, OryCun2.0, whole genome shotgun sequence | | |
| Oryctolagus_cuniculus | | >gi\|283562131\|ref\|NC_013686.1\| Oryctolagus cuniculus breed Thorbecke inbred chromosome 18, OryCun2.0, whole genome shotgun sequence | | |
| Oryctolagus_cuniculus | | >gi\|283562130\|ref\|NC_013687.1\| Oryctolagus cuniculus breed Thorbecke inbred chromosome 19, OryCun2.0, whole genome shotgun sequence | | |
| Oryctolagus_cuniculus | | >gi\|283562147\|ref\|NC_013670.1\| Oryctolagus cuniculus breed Thorbecke inbred chromosome 2, OryCun2.0, whole genome shotgun sequence | | |
| Oryctolagus_cuniculus | | >gi\|283562129\|ref\|NC_013688.1\| Oryctolagus cuniculus breed Thorbecke inbred chromosome 20, OryCun2.0, whole genome shotgun sequence | | |
| Oryctolagus_cuniculus | | >gi\|283562128\|ref\|NC_013689.1\| Oryctolagus cuniculus breed Thorbecke inbred chromosome 21, OryCun2.0, whole genome shotgun sequence | | |
| Oryctolagus_cuniculus | | >gi\|283562146\|ref\|NC_013671.1\| Oryctolagus cuniculus breed Thorbecke inbred chromosome 3, OryCun2.0, whole genome shotgun sequence | | |
| Oryctolagus_cuniculus | | >gi\|283562145\|ref\|NC_013672.1\| Oryctolagus cuniculus breed Thorbecke inbred chromosome 4, OryCun2.0, whole genome shotgun sequence | | |
| Oryctolagus_cuniculus | | >gi\|283562144\|ref\|NC_013673.1\| Oryctolagus cuniculus breed Thorbecke inbred chromosome 5, OryCun2.0, whole genome shotgun sequence | | |
| Oryctolagus_cuniculus | | >gi\|283562143\|ref\|NC_013674.1\| Oryctolagus cuniculus breed Thorbecke inbred chromosome 6, OryCun2.0, whole genome shotgun sequence | | |
| Oryctolagus_cuniculus | | >gi\|283562142\|ref\|NC_013675.1\| Oryctolagus cuniculus breed Thorbecke inbred chromosome 7, OryCun2.0, whole genome shotgun sequence | | |
| Oryctolagus_cuniculus | | >gi\|283562141\|ref\|NC_013676.1\| Oryctolagus cuniculus breed Thorbecke inbred chromosome 8, OryCun2.0, whole genome shotgun sequence | | |
| Oryctolagus_cuniculus | | >gi\|283562140\|ref\|NC_013677.1\| Oryctolagus cuniculus breed Thorbecke inbred chromosome 9, OryCun2.0, whole genome shotgun sequence | | |
| Oryctolagus_cuniculus | | >gi\|283562127\|ref\|NC_013690.1\| Oryctolagus cuniculus breed Thorbecke inbred chromosome X, OryCun2.0, whole genome shotgun sequence | | |
| Oryctolagus_cuniculus | | >gi\|283554652\|ref\|NW_003162542.1\| Oryctolagus cuniculus breed Thorbecke inbred unplaced genomic scaffold, OryCun2.0 chrUn3219, whole genome shotgun sequence | | |
| Oryza_sativa_japonica | | >ref\|NC_008394.1\|:1-43261740 Oryza sativa (japonica cultivar-group) genomic DNA, chromosome 1 | | |
| Oryza_sativa_japonica | | >ref\|NC_008403.1\|:1-22685906 Oryza sativa (japonica cultivar-group) genomic DNA, chromosome 10 | | |
| Oryza_sativa_japonica | | >ref\|NC_008404.1\|:1-28386948 Oryza sativa (japonica cultivar-group) genomic DNA, chromosome 11 | | |
| Oryza_sativa_japonica | | >ref\|NC_008405.1\|:1-27566993 Oryza sativa (japonica cultivar-group) genomic DNA, chromosome 12 | | |
| Oryza_sativa_japonica | | >ref\|NC_008395.1\|:1-35954743 Oryza sativa (japonica cultivar-group) genomic DNA, chromosome 2 | | |
| Oryza_sativa_japonica | | >ref\|NC_008396.1\|:1-36192742 Oryza sativa (japonica cultivar-group) genomic DNA, chromosome 3 | | |
| Oryza_sativa_japonica | | >ref\|NC_008397.1\|:1-35498469 Oryza sativa (japonica cultivar-group) genomic DNA, chromosome 4 | | |
| Oryza_sativa_japonica | | >ref\|NC_008398.1\|:1-29737217 Oryza sativa (japonica cultivar-group) genomic DNA, chromosome 5 | | |
| Oryza_sativa_japonica | | >ref\|NC_008399.1\|:1-30731886 Oryza sativa (japonica cultivar-group) genomic DNA, chromosome 6 | | |
| Oryza_sativa_japonica | | >ref\|NC_008400.1\|:1-29644043 Oryza sativa (japonica cultivar-group) genomic DNA, chromosome 7 | | |
| Oryza_sativa_japonica | | >ref\|NC_008401.1\|:1-28434780 Oryza sativa (japonica cultivar-group) genomic DNA, chromosome 8 | | |
| Oryza_sativa_japonica | | >ref\|NC_008402.1\|:1-22696651 Oryza sativa (japonica cultivar-group) genomic DNA, chromosome 9 | | |
| Pan_troglodytes | | >gi\|114795050\|ref\|NC_006468.2\|NC_006468 Pan troglodytes chromosome 1, reference assembly (based on Pan_troglodytes-2.1) | | |
| Pan_troglodytes | | >gi\|114795051\|ref\|NC_006477.2\|NC_006477 Pan troglodytes chromosome 10, reference assembly (based on Pan_troglodytes-2.1) | | |
| Pan_troglodytes | | >gi\|114795052\|ref\|NC_006478.2\|NC_006478 Pan troglodytes chromosome 11, reference assembly (based on Pan_troglodytes-2.1) | | |
| Pan_troglodytes | | >gi\|114795053\|ref\|NC_006479.2\|NC_006479 Pan troglodytes chromosome 12, reference assembly (based on Pan_troglodytes-2.1) | | |
| Pan_troglodytes | | >gi\|114795054\|ref\|NC_006480.2\|NC_006480 Pan troglodytes chromosome 13, reference assembly (based on Pan_troglodytes-2.1) | | |
| Pan_troglodytes | | >gi\|114795055\|ref\|NC_006481.2\|NC_006481 Pan troglodytes chromosome 14, reference assembly (based on Pan_troglodytes-2.1) | | |
| Pan_troglodytes | | >gi\|114795056\|ref\|NC_006482.2\|NC_006482 Pan troglodytes chromosome 15, reference assembly (based on Pan_troglodytes-2.1) | | |
| Pan_troglodytes | | >gi\|114795057\|ref\|NC_006483.2\|NC_006483 Pan troglodytes chromosome 16, reference assembly (based on Pan_troglodytes-2.1) | | |
| Pan_troglodytes | | >gi\|114795065\|ref\|NC_006484.2\|NC_006484 Pan troglodytes chromosome 17, reference assembly (based on Pan_troglodytes-2.1) | | |
| Pan_troglodytes | | >gi\|114795066\|ref\|NC_006485.2\|NC_006485 Pan troglodytes chromosome 18, reference assembly (based on Pan_troglodytes-2.1) | | |
| Pan_troglodytes | | >gi\|114795187\|ref\|NC_006486.2\|NC_006486 Pan troglodytes chromosome 19, reference assembly (based on Pan_troglodytes-2.1) | | |
| Pan_troglodytes | | >gi\|114795211\|ref\|NC_006487.2\|NC_006487 Pan troglodytes chromosome 20, reference assembly (based on Pan_troglodytes-2.1) | | |
| Pan_troglodytes | | >gi\|114795212\|ref\|NC_006488.2\|NC_006488 Pan troglodytes chromosome 21, reference assembly (based on Pan_troglodytes-2.1) | | |
| Pan_troglodytes | | >gi\|114795213\|ref\|NC_006489.2\|NC_006489 Pan troglodytes chromosome 22, reference assembly (based on Pan_troglodytes-2.1) | | |
| Pan_troglodytes | | >gi\|114795440\|ref\|NC_006469.2\|NC_006469 Pan troglodytes chromosome 2A, reference assembly (based on Pan_troglodytes-2.1) | | |
| Pan_troglodytes | | >gi\|114796131\|ref\|NC_006470.2\|NC_006470 Pan troglodytes chromosome 2B, reference assembly (based on Pan_troglodytes-2.1) | | |
| Pan_troglodytes | | >gi\|114796132\|ref\|NC_006490.2\|NC_006490 Pan troglodytes chromosome 3, reference assembly (based on Pan_troglodytes-2.1) | | |
| Pan_troglodytes | | >gi\|114796133\|ref\|NC_006471.2\|NC_006471 Pan troglodytes chromosome 4, reference assembly (based on Pan_troglodytes-2.1) | | |
| Pan_troglodytes | | >gi\|114796134\|ref\|NC_006472.2\|NC_006472 Pan troglodytes chromosome 5, reference assembly (based on Pan_troglodytes-2.1) | | |
| Pan_troglodytes | | >gi\|114796135\|ref\|NC_006473.2\|NC_006473 Pan troglodytes chromosome 6, reference assembly (based on Pan_troglodytes-2.1) | | |
| Pan_troglodytes | | >gi\|114796136\|ref\|NC_006474.2\|NC_006474 Pan troglodytes chromosome 7, reference assembly (based on Pan_troglodytes-2.1) | | |
| Pan_troglodytes | | >gi\|114796137\|ref\|NC_006475.2\|NC_006475 Pan troglodytes chromosome 8, reference assembly (based on Pan_troglodytes-2.1) | | |
| Pan_troglodytes | | >gi\|114796138\|ref\|NC_006476.2\|NC_006476 Pan troglodytes chromosome 9, reference assembly (based on Pan_troglodytes-2.1) | | |
| Pan_troglodytes | | >gi\|114796139\|ref\|NC_006491.2\|NC_006491 Pan troglodytes chromosome X, reference assembly (based on Pan_troglodytes-2.1) | | |
| Pan_troglodytes | | >gi\|114796141\|ref\|NC_006492.2\|NC_006492 Pan troglodytes chromosome Y, reference assembly (based on Pan_troglodytes-2.1) | | |
| Plasmodium_falciparum | | >gi\|254922366\|gb\|AE014185.2\| Plasmodium falciparum 3D7 chromosome 10, complete sequence | | |
| Pongo_abelii | | >gi\|5835834\|ref\|NC_002083.1\| Pongo abelii mitochondrion, complete genome | | |
| Pongo_abelii | | >gi\|241864942\|ref\|NC_012591.1\| Pongo abelii chromosome 1, P_pygmaeus_2.0.2 | | |
| Pongo_abelii | | >gi\|241864932\|ref\|NC_012601.1\| Pongo abelii chromosome 10, P_pygmaeus_2.0.2 | | |
| Pongo_abelii | | >gi\|241864931\|ref\|NC_012602.1\| Pongo abelii chromosome 11, P_pygmaeus_2.0.2 | | |
| Pongo_abelii | | >gi\|241864930\|ref\|NC_012603.1\| Pongo abelii chromosome 12, P_pygmaeus_2.0.2 | | |
| Pongo_abelii | | >gi\|241864929\|ref\|NC_012604.1\| Pongo abelii chromosome 13, P_pygmaeus_2.0.2 | | |
| Pongo_abelii | | >gi\|241864928\|ref\|NC_012605.1\| Pongo abelii chromosome 14, P_pygmaeus_2.0.2 | | |
| Pongo_abelii | | >gi\|241864927\|ref\|NC_012606.1\| Pongo abelii chromosome 15, P_pygmaeus_2.0.2 | | |
| Pongo_abelii | | >gi\|241864926\|ref\|NC_012607.1\| Pongo abelii chromosome 16, P_pygmaeus_2.0.2 | | |
| Pongo_abelii | | >gi\|241864925\|ref\|NC_012608.1\| Pongo abelii chromosome 17, P_pygmaeus_2.0.2 | | |
| Pongo_abelii | | >gi\|241864924\|ref\|NC_012609.1\| Pongo abelii chromosome 18, P_pygmaeus_2.0.2 | | |
| Pongo_abelii | | >gi\|241864923\|ref\|NC_012610.1\| Pongo abelii chromosome 19, P_pygmaeus_2.0.2 | | |
| Pongo_abelii | | >gi\|241864922\|ref\|NC_012611.1\| Pongo abelii chromosome 20, P_pygmaeus_2.0.2 | | |
| Pongo_abelii | | >gi\|241864921\|ref\|NC_012612.1\| Pongo abelii chromosome 21, P_pygmaeus_2.0.2 | | |
| Pongo_abelii | | >gi\|241864895\|ref\|NC_012613.1\| Pongo abelii chromosome 22, P_pygmaeus_2.0.2 | | |
| Pongo_abelii | | >gi\|241864941\|ref\|NC_012592.1\| Pongo abelii chromosome 2A, P_pygmaeus_2.0.2 | | |
| Pongo_abelii | | >gi\|241864940\|ref\|NC_012593.1\| Pongo abelii chromosome 2B, P_pygmaeus_2.0.2 | | |
| Pongo_abelii | | >gi\|241864939\|ref\|NC_012594.1\| Pongo abelii chromosome 3, P_pygmaeus_2.0.2 | | |
| Pongo_abelii | | >gi\|241864938\|ref\|NC_012595.1\| Pongo abelii chromosome 4, P_pygmaeus_2.0.2 | | |
| Pongo_abelii | | >gi\|241864937\|ref\|NC_012596.1\| Pongo abelii chromosome 5, P_pygmaeus_2.0.2 | | |
| Pongo_abelii | | >gi\|241864936\|ref\|NC_012597.1\| Pongo abelii chromosome 6, P_pygmaeus_2.0.2 | | |
| Pongo_abelii | | >gi\|241864935\|ref\|NC_012598.1\| Pongo abelii chromosome 7, P_pygmaeus_2.0.2 | | |
| Pongo_abelii | | >gi\|241864934\|ref\|NC_012599.1\| Pongo abelii chromosome 8, P_pygmaeus_2.0.2 | | |
| Pongo_abelii | | >gi\|241864933\|ref\|NC_012600.1\| Pongo abelii chromosome 9, P_pygmaeus_2.0.2 | | |
| Pongo_abelii | | >gi\|241864894\|ref\|NC_012614.1\| Pongo abelii chromosome X, P_pygmaeus_2.0.2 | | |
| Pongo_abelii | | >gi\|240988231\|ref\|NW_002972004.1\| Pongo abelii chromosome X unlocalized genomic scaffold, P_pygmaeus_2.0.2 | | |
| Pongo_abelii | | >gi\|240968845\|ref\|NW_002973204.1\| Pongo abelii unplaced genomic scaffold, P_pygmaeus_2.0.2 | | |
| Populus_trichocarpa | | >gi\|156627641\|gb\|AC209224.1\| Populus trichocarpa chromosome POP064-N07, complete sequence | | |
| Populus_trichocarpa | | >gi\|88853762\|gb\|AC182672.2\| Populus trichocarpa chromosome Pop1-25F8, complete sequence | | |
| Populus_trichocarpa | | >gi\|116047841\|gb\|CM000337.1\| Populus trichocarpa linkage group I chromosome, whole genome shotgun sequence | | |
| Populus_trichocarpa | | >gi\|116047832\|gb\|CM000346.1\| Populus trichocarpa linkage group X chromosome, whole genome shotgun sequence | | |
| Populus_trichocarpa | | >gi\|116047831\|gb\|CM000347.1\| Populus trichocarpa linkage group XI chromosome, whole genome shotgun sequence | | |
| Populus_trichocarpa | | >gi\|116047830\|gb\|CM000348.1\| Populus trichocarpa linkage group XII chromosome, whole genome shotgun sequence | | |
| Populus_trichocarpa | | >gi\|116047829\|gb\|CM000349.1\| Populus trichocarpa linkage group XIII chromosome, whole genome shotgun sequence | | |
| Populus_trichocarpa | | >gi\|116047828\|gb\|CM000350.1\| Populus trichocarpa linkage group XIV chromosome, whole genome shotgun sequence | | |
| Populus_trichocarpa | | >gi\|116047827\|gb\|CM000351.1\| Populus trichocarpa linkage group XV chromosome, whole genome shotgun sequence | | |
| Populus_trichocarpa | | >gi\|116047826\|gb\|CM000352.1\| Populus trichocarpa linkage group XVI chromosome, whole genome shotgun sequence | | |
| Populus_trichocarpa | | >gi\|116047825\|gb\|CM000353.1\| Populus trichocarpa linkage group XVII chromosome, whole genome shotgun sequence | | |
| Populus_trichocarpa | | >gi\|116047824\|gb\|CM000354.1\| Populus trichocarpa linkage group XVIII chromosome, whole genome shotgun sequence | | |
| Populus_trichocarpa | | >gi\|116047823\|gb\|CM000355.1\| Populus trichocarpa linkage group XIX chromosome, whole genome shotgun sequence | | |
| Populus_trichocarpa | | >gi\|116047839\|gb\|CM000339.1\| Populus trichocarpa linkage group III chromosome, whole genome shotgun sequence | | |
| Populus_trichocarpa | | >gi\|116047838\|gb\|CM000340.1\| Populus trichocarpa linkage group IV chromosome, whole genome shotgun sequence | | |
| Populus_trichocarpa | | >gi\|116047837\|gb\|CM000341.1\| Populus trichocarpa linkage group V chromosome, whole genome shotgun sequence | | |
| Populus_trichocarpa | | >gi\|116047836\|gb\|CM000342.1\| Populus trichocarpa linkage group VI chromosome, whole genome shotgun sequence | | |
| Populus_trichocarpa | | >gi\|116047835\|gb\|CM000343.1\| Populus trichocarpa linkage group VII chromosome, whole genome shotgun sequence | | |
| Populus_trichocarpa | | >gi\|116047834\|gb\|CM000344.1\| Populus trichocarpa linkage group VIII chromosome, whole genome shotgun sequence | | |
| Populus_trichocarpa | | >gi\|116047833\|gb\|CM000345.1\| Populus trichocarpa linkage group IX chromosome, whole genome shotgun sequence | | |
| Rattus__norvegicus | | >gi\|109644432\|ref\|AC_000069.1\| Rattus norvegicus chromosome 1, alternate assembly Rn_Celera, whole genome shotgun sequence | | |
| Rattus__norvegicus | | >gi\|109649548\|ref\|AC_000078.1\| Rattus norvegicus chromosome 10, alternate assembly Rn_Celera, whole genome shotgun sequence | | |
| Rattus__norvegicus | | >gi\|109649549\|ref\|AC_000079.1\| Rattus norvegicus chromosome 11, alternate assembly Rn_Celera, whole genome shotgun sequence | | |
| Rattus__norvegicus | | >gi\|109649550\|ref\|AC_000080.1\| Rattus norvegicus chromosome 12, alternate assembly Rn_Celera, whole genome shotgun sequence | | |
| Rattus__norvegicus | | >gi\|109649551\|ref\|AC_000081.1\| Rattus norvegicus chromosome 13, alternate assembly Rn_Celera, whole genome shotgun sequence | | |
| Rattus__norvegicus | | >gi\|109649552\|ref\|AC_000082.1\| Rattus norvegicus chromosome 14, alternate assembly Rn_Celera, whole genome shotgun sequence | | |
| Rattus__norvegicus | | >gi\|109649553\|ref\|AC_000083.1\| Rattus norvegicus chromosome 15, alternate assembly Rn_Celera, whole genome shotgun sequence | | |
| Rattus__norvegicus | | >gi\|109649554\|ref\|AC_000084.1\| Rattus norvegicus chromosome 16, alternate assembly Rn_Celera, whole genome shotgun sequence | | |
| Rattus__norvegicus | | >gi\|109649555\|ref\|AC_000085.1\| Rattus norvegicus chromosome 17, alternate assembly Rn_Celera, whole genome shotgun sequence | | |
| Rattus__norvegicus | | >gi\|109649556\|ref\|AC_000086.1\| Rattus norvegicus chromosome 18, alternate assembly Rn_Celera, whole genome shotgun sequence | | |
| Rattus__norvegicus | | >gi\|109649557\|ref\|AC_000087.1\| Rattus norvegicus chromosome 19, alternate assembly Rn_Celera, whole genome shotgun sequence | | |
| Rattus__norvegicus | | >gi\|109649558\|ref\|AC_000070.1\| Rattus norvegicus chromosome 2, alternate assembly Rn_Celera, whole genome shotgun sequence | | |
| Rattus__norvegicus | | >gi\|109649721\|ref\|AC_000088.1\| Rattus norvegicus chromosome 20, alternate assembly Rn_Celera, whole genome shotgun sequence | | |
| Rattus__norvegicus | | >gi\|109649722\|ref\|AC_000071.1\| Rattus norvegicus chromosome 3, alternate assembly Rn_Celera, whole genome shotgun sequence | | |
| Rattus__norvegicus | | >gi\|109652486\|ref\|AC_000072.1\| Rattus norvegicus chromosome 4, alternate assembly Rn_Celera, whole genome shotgun sequence | | |
| Rattus__norvegicus | | >gi\|109657628\|ref\|AC_000073.1\| Rattus norvegicus chromosome 5, alternate assembly Rn_Celera, whole genome shotgun sequence | | |
| Rattus__norvegicus | | >gi\|109658137\|ref\|AC_000074.1\| Rattus norvegicus chromosome 6, alternate assembly Rn_Celera, whole genome shotgun sequence | | |
| Rattus__norvegicus | | >gi\|109658138\|ref\|AC_000075.1\| Rattus norvegicus chromosome 7, alternate assembly Rn_Celera, whole genome shotgun sequence | | |
| Rattus__norvegicus | | >gi\|109658147\|ref\|AC_000076.1\| Rattus norvegicus chromosome 8, alternate assembly Rn_Celera, whole genome shotgun sequence | | |
| Rattus__norvegicus | | >gi\|109658148\|ref\|AC_000077.1\| Rattus norvegicus chromosome 9, alternate assembly Rn_Celera, whole genome shotgun sequence | | |
| Rattus__norvegicus | | >gi\|109658149\|ref\|AC_000089.1\| Rattus norvegicus chromosome X, alternate assembly Rn_Celera, whole genome shotgun sequence | | |
| Rattus__norvegicus | | >gi\|109522708\|ref\|NW_001091847.1\| Rattus norvegicus unplaced genomic scaffold, alternate assembly Rn_Celera CRA_213000034259910, whole genome shotgun sequence | | |
| Rattus__norvegicus | | >gi\|110189714\|ref\|NC_001665.2\| Rattus norvegicus strain BN/SsNHsdMCW mitochondrion, complete genome | | |
| Rattus__norvegicus | | >gi\|62750345\|ref\|NC_005100.2\| Rattus norvegicus strain BN/SsNHsdMCW chromosome 1, RGSC_v3.4 | | |
| Rattus__norvegicus | | >gi\|62750810\|ref\|NC_005109.2\| Rattus norvegicus strain BN/SsNHsdMCW chromosome 10, RGSC_v3.4 | | |
| Rattus__norvegicus | | >gi\|62750811\|ref\|NC_005110.2\| Rattus norvegicus strain BN/SsNHsdMCW chromosome 11, RGSC_v3.4 | | |
| Rattus__norvegicus | | >gi\|62750812\|ref\|NC_005111.2\| Rattus norvegicus strain BN/SsNHsdMCW chromosome 12, RGSC_v3.4 | | |
| Rattus__norvegicus | | >gi\|62750813\|ref\|NC_005112.2\| Rattus norvegicus strain BN/SsNHsdMCW chromosome 13, RGSC_v3.4 | | |
| Rattus__norvegicus | | >gi\|62750814\|ref\|NC_005113.2\| Rattus norvegicus strain BN/SsNHsdMCW chromosome 14, RGSC_v3.4 | | |
| Rattus__norvegicus | | >gi\|62750815\|ref\|NC_005114.2\| Rattus norvegicus strain BN/SsNHsdMCW chromosome 15, RGSC_v3.4 | | |
| Rattus__norvegicus | | >gi\|62750816\|ref\|NC_005115.2\| Rattus norvegicus strain BN/SsNHsdMCW chromosome 16, RGSC_v3.4 | | |
| Rattus__norvegicus | | >gi\|62750817\|ref\|NC_005116.2\| Rattus norvegicus strain BN/SsNHsdMCW chromosome 17, RGSC_v3.4 | | |
| Rattus__norvegicus | | >gi\|62750818\|ref\|NC_005117.2\| Rattus norvegicus strain BN/SsNHsdMCW chromosome 18, RGSC_v3.4 | | |
| Rattus__norvegicus | | >gi\|62750819\|ref\|NC_005118.2\| Rattus norvegicus strain BN/SsNHsdMCW chromosome 19, RGSC_v3.4 | | |
| Rattus__norvegicus | | >gi\|62750359\|ref\|NC_005101.2\| Rattus norvegicus strain BN/SsNHsdMCW chromosome 2, RGSC_v3.4 | | |
| Rattus__norvegicus | | >gi\|62750820\|ref\|NC_005119.2\| Rattus norvegicus strain BN/SsNHsdMCW chromosome 20, RGSC_v3.4 | | |
| Rattus__norvegicus | | >gi\|62750360\|ref\|NC_005102.2\| Rattus norvegicus strain BN/SsNHsdMCW chromosome 3, RGSC_v3.4 | | |
| Rattus__norvegicus | | >gi\|62750804\|ref\|NC_005103.2\| Rattus norvegicus strain BN/SsNHsdMCW chromosome 4, RGSC_v3.4 | | |
| Rattus__norvegicus | | >gi\|62750805\|ref\|NC_005104.2\| Rattus norvegicus strain BN/SsNHsdMCW chromosome 5, RGSC_v3.4 | | |
| Rattus__norvegicus | | >gi\|62750806\|ref\|NC_005105.2\| Rattus norvegicus strain BN/SsNHsdMCW chromosome 6, RGSC_v3.4 | | |
| Rattus__norvegicus | | >gi\|62750807\|ref\|NC_005106.2\| Rattus norvegicus strain BN/SsNHsdMCW chromosome 7, RGSC_v3.4 | | |
| Rattus__norvegicus | | >gi\|62750808\|ref\|NC_005107.2\| Rattus norvegicus strain BN/SsNHsdMCW chromosome 8, RGSC_v3.4 | | |
| Rattus__norvegicus | | >gi\|62750809\|ref\|NC_005108.2\| Rattus norvegicus strain BN/SsNHsdMCW chromosome 9, RGSC_v3.4 | | |
| Rattus__norvegicus | | >gi\|62750821\|ref\|NC_005120.2\| Rattus norvegicus strain BN/SsNHsdMCW chromosome X, RGSC_v3.4 | | |
| Rattus__norvegicus | | >gi\|34881901\|ref\|NW_048060.1\| Rattus norvegicus strain BN/SsNHsdMCW chromosome X unlocalized genomic scaffold, RGSC_v3.4 | | |
| Rattus__norvegicus | | >gi\|34882973\|ref\|NW_047942.1\| Rattus norvegicus strain BN/SsNHsdMCW unplaced genomic scaffold, RGSC_v3.4 | | |
| Saccharomyces_cerevisiae_uid128 | | >Saccharomyces cerevisiae S288c chromosome I, complete sequence. | | |
| Saccharomyces_cerevisiae_uid128 | | >Saccharomyces cerevisiae S288c chromosome II, complete sequence. | | |
| Saccharomyces_cerevisiae_uid128 | | >Saccharomyces cerevisiae S288c chromosome III, complete sequence. | | |
| Saccharomyces_cerevisiae_uid128 | | >Saccharomyces cerevisiae S288c chromosome IV, complete sequence. | | |
| Saccharomyces_cerevisiae_uid128 | | >Saccharomyces cerevisiae S288c chromosome V, complete sequence. | | |
| Saccharomyces_cerevisiae_uid128 | | >Saccharomyces cerevisiae S288c chromosome VI, complete sequence. | | |
| Saccharomyces_cerevisiae_uid128 | | >Saccharomyces cerevisiae S288c chromosome VII, complete sequence. | | |
| Saccharomyces_cerevisiae_uid128 | | >Saccharomyces cerevisiae S288c chromosome VIII, complete sequence. | | |
| Saccharomyces_cerevisiae_uid128 | | >Saccharomyces cerevisiae S288c chromosome IX, complete sequence. | | |
| Saccharomyces_cerevisiae_uid128 | | >Saccharomyces cerevisiae S288c chromosome X, complete sequence. | | |
| Saccharomyces_cerevisiae_uid128 | | >Saccharomyces cerevisiae S288c chromosome XI, complete sequence. | | |
| Saccharomyces_cerevisiae_uid128 | | >Saccharomyces cerevisiae S288c chromosome XII, complete sequence. | | |
| Saccharomyces_cerevisiae_uid128 | | >Saccharomyces cerevisiae S288c chromosome XIII, complete sequence. | | |
| Saccharomyces_cerevisiae_uid128 | | >Saccharomyces cerevisiae S288c chromosome XIV, complete sequence. | | |
| Saccharomyces_cerevisiae_uid128 | | >Saccharomyces cerevisiae S288c chromosome XV, complete sequence. | | |
| Saccharomyces_cerevisiae_uid128 | | >Saccharomyces cerevisiae S288c chromosome XVI, complete sequence. | | |
| Saccharomyces_cerevisiae_uid128 | | >Saccharomyces cerevisiae S288c mitochondrion, complete genome. | | |
| Schizosaccharomyces_pombe | | >Schizosaccharomyces pombe mitochondrion, complete genome. | | |
| Schizosaccharomyces_pombe | | >Schizosaccharomyces pombe 972h- chromosome III, complete sequence. | | |
| Schizosaccharomyces_pombe | | >Schizosaccharomyces pombe 972h- chromosome II, complete sequence. | | |
| Schizosaccharomyces_pombe | | >Schizosaccharomyces pombe 972h- chromosome I, complete sequence. | | |
| Solanum_lycopersicum | | >gi\|290755767\|gb\|AC238920.11\| and GI for 1352 clones (not listed). | | |
| Solanum_lycopersicum | | gi\|322718807\|gb\|CM001064.1\| Solanum lycopersicum chromosome 1, whole genome shotgun sequence | | |
| Solanum_lycopersicum | | gi\|322718806\|gb\|CM001065.1\| Solanum lycopersicum chromosome 2, whole genome shotgun sequence  gi\|322718806\|gb\|CM001065.1\| Solanum lycopersicum chromosome 2, whole genome shotgun sequence | | |
| Solanum_lycopersicum | | gi\|322718806\|gb\|CM001065.1\| Solanum lycopersicum chromosome 2, whole genome shotgun sequence | | |
| Solanum_lycopersicum | | gi\|322718804\|gb\|CM001067.1\| Solanum lycopersicum chromosome 4, whole genome shotgun sequence | | |
| Solanum_lycopersicum | | gi\|322718803\|gb\|CM001068.1\| Solanum lycopersicum chromosome 5, whole genome shotgun sequence | | |
| Solanum_lycopersicum | | gi\|322718802\|gb\|CM001069.1\| Solanum lycopersicum chromosome 6, whole genome shotgun sequence (and chr 8.) | | |
| Solanum_lycopersicum | | gi\|322718801\|gb\|CM001070.1\| Solanum lycopersicum chromosome 7, whole genome shotgun sequence  gi\|322718799\|gb\|CM001072.1\| Solanum lycopersicum chromosome 9, whole genome shotgun sequence  gi\|322718798\|gb\|CM001073.1\| Solanum lycopersicum chromosome 10, whole genome shotgun sequence; and Chr 12  gi\|322718797\|gb\|CM001074.1\| Solanum lycopersicum chromosome 11, whole genome shotgun sequence |  |  |
| Solanum_tuberosum | >chr01 PGSC_DM_v4.03_pseudomolecules.fasta.zip | | |  |
| Solanum_tuberosum | >chr02 | | |  |
| Solanum_tuberosum | >chr03 | | |  |
| Solanum_tuberosum | >chr04 | | |  |
| Solanum_tuberosum | >chr05 | | |  |
| Solanum_tuberosum | >chr06 | | |  |
| Solanum_tuberosum | >chr07 | | |  |
| Solanum_tuberosum | >chr08 | | |  |
| Solanum_tuberosum | >chr09 | | |  |
| Solanum_tuberosum | >chr10 | | |  |
| Solanum_tuberosum | >chr11 | | |  |
| Solanum_tuberosum | >chr12 | | |  |
| Solanum_phureja_DM | | >PGSC0003DMO000068169 | | |
| Solanum_tuberosum_RH_bacs | | >gi\|197252128\|gb\|AC232062.1\| Solanum tuberosum strain Diploid genotype RH89-039-16 chromosome 1 clone RH084F08, *** SEQUENCING IN PROGRESS ***, 15 unordered pieces. | | |
| Sorghum_bicolor | | >gi\|242042636\|ref\|NC_012870.1\| Sorghum bicolor chromosome 1, whole genome shotgun sequence | | |
| Sorghum_bicolor | | >gi\|242097191\|ref\|NC_012879.1\| Sorghum bicolor chromosome 10, whole genome shotgun sequence | | |
| Sorghum_bicolor | | >gi\|242060088\|ref\|NC_012872.1\| Sorghum bicolor chromosome 3, whole genome shotgun sequence | | |
| Sorghum_bicolor | | >gi\|242067135\|ref\|NC_012873.1\| Sorghum bicolor chromosome 4, whole genome shotgun sequence | | |
| Sorghum_bicolor | | >gi\|242072114\|ref\|NC_012874.1\| Sorghum bicolor chromosome 5, whole genome shotgun sequence | | |
| Sorghum_bicolor | | >gi\|242077817\|ref\|NC_012875.1\| Sorghum bicolor chromosome 6, whole genome shotgun sequence | | |
| Sorghum_bicolor | | >gi\|242082450\|ref\|NC_012876.1\| Sorghum bicolor chromosome 7, whole genome shotgun sequence | | |
| Sorghum_bicolor | | >gi\|242086505\|ref\|NC_012877.1\| Sorghum bicolor chromosome 8, whole genome shotgun sequence | | |
| Sorghum_bicolor | | >gi\|242091636\|ref\|NC_012878.1\| Sorghum bicolor chromosome 9, whole genome shotgun sequence | | |
| Sus_scrofa | | >gi\|5835862\|ref\|NC_000845.1\| Sus scrofa mitochondrion, complete genome | | |
| Sus_scrofa | | >gi\|298162961\|ref\|NC_010443.2\| Sus scrofa breed mixed chromosome 1, Sscrofa9.2 | | |
| Sus_scrofa | | >gi\|298162952\|ref\|NC_010452.1\| Sus scrofa breed mixed chromosome 10, Sscrofa9.2 | | |
| Sus_scrofa | | >gi\|298162951\|ref\|NC_010453.2\| Sus scrofa breed mixed chromosome 11, Sscrofa9.2 | | |
| Sus_scrofa | | >gi\|298162950\|ref\|NC_010454.1\| Sus scrofa breed mixed chromosome 12, Sscrofa9.2 | | |
| Sus_scrofa | | >gi\|298162949\|ref\|NC_010455.2\| Sus scrofa breed mixed chromosome 13, Sscrofa9.2 | | |
| Sus_scrofa | | >gi\|298162948\|ref\|NC_010456.2\| Sus scrofa breed mixed chromosome 14, Sscrofa9.2 | | |
| Sus_scrofa | | >gi\|298162947\|ref\|NC_010457.2\| Sus scrofa breed mixed chromosome 15, Sscrofa9.2 | | |
| Sus_scrofa | | >gi\|298162946\|ref\|NC_010458.1\| Sus scrofa breed mixed chromosome 16, Sscrofa9.2 | | |
| Sus_scrofa | | >gi\|298162945\|ref\|NC_010459.2\| Sus scrofa breed mixed chromosome 17, Sscrofa9.2 | | |
| Sus_scrofa | | >gi\|298162944\|ref\|NC_010460.1\| Sus scrofa breed mixed chromosome 18, Sscrofa9.2 | | |
| Sus_scrofa | | >gi\|298162960\|ref\|NC_010444.1\| Sus scrofa breed mixed chromosome 2, Sscrofa9.2 | | |
| Sus_scrofa | | >gi\|298162959\|ref\|NC_010445.1\| Sus scrofa breed mixed chromosome 3, Sscrofa9.2 | | |
| Sus_scrofa | | >gi\|298162958\|ref\|NC_010446.2\| Sus scrofa breed mixed chromosome 4, Sscrofa9.2 | | |
| Sus_scrofa | | >gi\|298162957\|ref\|NC_010447.2\| Sus scrofa breed mixed chromosome 5, Sscrofa9.2 | | |
| Sus_scrofa | | >gi\|298162956\|ref\|NC_010448.1\| Sus scrofa breed mixed chromosome 6, Sscrofa9.2 | | |
| Sus_scrofa | | >gi\|298162955\|ref\|NC_010449.2\| Sus scrofa breed mixed chromosome 7, Sscrofa9.2 | | |
| Sus_scrofa | | >gi\|298162954\|ref\|NC_010450.1\| Sus scrofa breed mixed chromosome 8, Sscrofa9.2 | | |
| Sus_scrofa | | >gi\|298162953\|ref\|NC_010451.1\| Sus scrofa breed mixed chromosome 9, Sscrofa9.2 | | |
| Sus_scrofa | | >gi\|298162943\|ref\|NC_010461.2\| Sus scrofa breed mixed chromosome X, Sscrofa9.2 | | |
| Taeniopygia_guttata | | >gi\|224381666\|ref\|NC_011462.1\|NC_011462 Taeniopygia guttata chromosome 1, reference assembly (based on Taeniopygia_guttata-3.2.4), whole genome shotgun sequence | | |
| Taeniopygia_guttata | | >gi\|224381667\|ref\|NC_011474.1\|NC_011474 Taeniopygia guttata chromosome 10, reference assembly (based on Taeniopygia_guttata-3.2.4), whole genome shotgun sequence | | |
| Taeniopygia_guttata | | >gi\|224381668\|ref\|NC_011475.1\|NC_011475 Taeniopygia guttata chromosome 11, reference assembly (based on Taeniopygia_guttata-3.2.4), whole genome shotgun sequence | | |
| Taeniopygia_guttata | | >gi\|224381669\|ref\|NC_011476.1\|NC_011476 Taeniopygia guttata chromosome 12, reference assembly (based on Taeniopygia_guttata-3.2.4), whole genome shotgun sequence | | |
| Taeniopygia_guttata | | >gi\|224381670\|ref\|NC_011477.1\|NC_011477 Taeniopygia guttata chromosome 13, reference assembly (based on Taeniopygia_guttata-3.2.4), whole genome shotgun sequence | | |
| Taeniopygia_guttata | | >gi\|224381671\|ref\|NC_011478.1\|NC_011478 Taeniopygia guttata chromosome 14, reference assembly (based on Taeniopygia_guttata-3.2.4), whole genome shotgun sequence | | |
| Taeniopygia_guttata | | >gi\|224381672\|ref\|NC_011479.1\|NC_011479 Taeniopygia guttata chromosome 15, reference assembly (based on Taeniopygia_guttata-3.2.4), whole genome shotgun sequence | | |
| Taeniopygia_guttata | | >gi\|224381673\|ref\|NC_011480.1\|NC_011480 Taeniopygia guttata chromosome 16, reference assembly (based on Taeniopygia_guttata-3.2.4), whole genome shotgun sequence | | |
| Taeniopygia_guttata | | >gi\|224381674\|ref\|NC_011481.1\|NC_011481 Taeniopygia guttata chromosome 17, reference assembly (based on Taeniopygia_guttata-3.2.4), whole genome shotgun sequence | | |
| Taeniopygia_guttata | | >gi\|224381675\|ref\|NC_011482.1\|NC_011482 Taeniopygia guttata chromosome 18, reference assembly (based on Taeniopygia_guttata-3.2.4), whole genome shotgun sequence | | |
| Taeniopygia_guttata | | >gi\|224381676\|ref\|NC_011483.1\|NC_011483 Taeniopygia guttata chromosome 19, reference assembly (based on Taeniopygia_guttata-3.2.4), whole genome shotgun sequence | | |
| Taeniopygia_guttata | | >gi\|224381677\|ref\|NC_011463.1\|NC_011463 Taeniopygia guttata chromosome 1A, reference assembly (based on Taeniopygia_guttata-3.2.4), whole genome shotgun sequence | | |
| Taeniopygia_guttata | | >gi\|224381678\|ref\|NC_011464.1\|NC_011464 Taeniopygia guttata chromosome 1B, reference assembly (based on Taeniopygia_guttata-3.2.4), whole genome shotgun sequence | | |
| Taeniopygia_guttata | | >gi\|224381679\|ref\|NC_011465.1\|NC_011465 Taeniopygia guttata chromosome 2, reference assembly (based on Taeniopygia_guttata-3.2.4), whole genome shotgun sequence | | |
| Taeniopygia_guttata | | >gi\|224381680\|ref\|NC_011484.1\|NC_011484 Taeniopygia guttata chromosome 20, reference assembly (based on Taeniopygia_guttata-3.2.4), whole genome shotgun sequence | | |
| Taeniopygia_guttata | | >gi\|224381681\|ref\|NC_011485.1\|NC_011485 Taeniopygia guttata chromosome 21, reference assembly (based on Taeniopygia_guttata-3.2.4), whole genome shotgun sequence | | |
| Taeniopygia_guttata | | >gi\|224381682\|ref\|NC_011486.1\|NC_011486 Taeniopygia guttata chromosome 22, reference assembly (based on Taeniopygia_guttata-3.2.4), whole genome shotgun sequence | | |
| Taeniopygia_guttata | | >gi\|224381683\|ref\|NC_011487.1\|NC_011487 Taeniopygia guttata chromosome 23, reference assembly (based on Taeniopygia_guttata-3.2.4), whole genome shotgun sequence | | |
| Taeniopygia_guttata | | >gi\|224381684\|ref\|NC_011488.1\|NC_011488 Taeniopygia guttata chromosome 24, reference assembly (based on Taeniopygia_guttata-3.2.4), whole genome shotgun sequence | | |
| Taeniopygia_guttata | | >gi\|224381685\|ref\|NC_011489.1\|NC_011489 Taeniopygia guttata chromosome 25, reference assembly (based on Taeniopygia_guttata-3.2.4), whole genome shotgun sequence | | |
| Taeniopygia_guttata | | >gi\|224381686\|ref\|NC_011490.1\|NC_011490 Taeniopygia guttata chromosome 26, reference assembly (based on Taeniopygia_guttata-3.2.4), whole genome shotgun sequence | | |
| Taeniopygia_guttata | | >gi\|224381687\|ref\|NC_011491.1\|NC_011491 Taeniopygia guttata chromosome 27, reference assembly (based on Taeniopygia_guttata-3.2.4), whole genome shotgun sequence | | |
| Taeniopygia_guttata | | >gi\|224381688\|ref\|NC_011492.1\|NC_011492 Taeniopygia guttata chromosome 28, reference assembly (based on Taeniopygia_guttata-3.2.4), whole genome shotgun sequence | | |
| Taeniopygia_guttata | | >gi\|224381689\|ref\|NC_011466.1\|NC_011466 Taeniopygia guttata chromosome 3, reference assembly (based on Taeniopygia_guttata-3.2.4), whole genome shotgun sequence | | |
| Taeniopygia_guttata | | >gi\|224381690\|ref\|NC_011467.1\|NC_011467 Taeniopygia guttata chromosome 4, reference assembly (based on Taeniopygia_guttata-3.2.4), whole genome shotgun sequence | | |
| Taeniopygia_guttata | | >gi\|224381691\|ref\|NC_011468.1\|NC_011468 Taeniopygia guttata chromosome 4A, reference assembly (based on Taeniopygia_guttata-3.2.4), whole genome shotgun sequence | | |
| Taeniopygia_guttata | | >gi\|224381692\|ref\|NC_011469.1\|NC_011469 Taeniopygia guttata chromosome 5, reference assembly (based on Taeniopygia_guttata-3.2.4), whole genome shotgun sequence | | |
| Taeniopygia_guttata | | >gi\|224381693\|ref\|NC_011470.1\|NC_011470 Taeniopygia guttata chromosome 6, reference assembly (based on Taeniopygia_guttata-3.2.4), whole genome shotgun sequence | | |
| Taeniopygia_guttata | | >gi\|224381694\|ref\|NC_011471.1\|NC_011471 Taeniopygia guttata chromosome 7, reference assembly (based on Taeniopygia_guttata-3.2.4), whole genome shotgun sequence | | |
| Taeniopygia_guttata | | >gi\|224381695\|ref\|NC_011472.1\|NC_011472 Taeniopygia guttata chromosome 8, reference assembly (based on Taeniopygia_guttata-3.2.4), whole genome shotgun sequence | | |
| Taeniopygia_guttata | | >gi\|224381696\|ref\|NC_011473.1\|NC_011473 Taeniopygia guttata chromosome 9, reference assembly (based on Taeniopygia_guttata-3.2.4), whole genome shotgun sequence | | |
| Taeniopygia_guttata | | >gi\|224381697\|ref\|NC_011494.1\|NC_011494 Taeniopygia guttata linkage group 2, reference assembly (based on Taeniopygia_guttata-3.2.4), whole genome shotgun sequence | | |
| Taeniopygia_guttata | | >gi\|224381698\|ref\|NC_011495.1\|NC_011495 Taeniopygia guttata linkage group 5, reference assembly (based on Taeniopygia_guttata-3.2.4), whole genome shotgun sequence | | |
| Taeniopygia_guttata | | >gi\|224381699\|ref\|NC_011496.1\|NC_011496 Taeniopygia guttata linkage group E22, reference assembly (based on Taeniopygia_guttata-3.2.4), whole genome shotgun sequence | | |
| Taeniopygia_guttata | | >gi\|224381700\|ref\|NC_011493.1\|NC_011493 Taeniopygia guttata chromosome Z, reference assembly (based on Taeniopygia_guttata-3.2.4), whole genome shotgun sequence | | |
| Takifugu_rubripes | | >gi\|22430444\|emb\|CAAB01012381.1\| Fugu rubripes whole genome shotgun assembly CONTIG_63105, whole genome shotgun sequence | | |
| Tribolium_castaneum | | >gi\|189313711\|ref\|NC_007425.2\|NC_007425 Tribolium castaneum linkage group 10, reference assembly (based on Tcas_3.0), whole genome shotgun sequence | | |
| Tribolium_castaneum | | >gi\|189313713\|ref\|NC_007417.2\|NC_007417 Tribolium castaneum linkage group 2, reference assembly (based on Tcas_3.0), whole genome shotgun sequence | | |
| Tribolium_castaneum | | >gi\|189313714\|ref\|NC_007418.2\|NC_007418 Tribolium castaneum linkage group 3, reference assembly (based on Tcas_3.0), whole genome shotgun sequence | | |
| Tribolium_castaneum | | >gi\|189313718\|ref\|NC_007423.2\|NC_007423 Tribolium castaneum linkage group 8, reference assembly (based on Tcas_3.0), whole genome shotgun sequence | | |
| Tribolium_castaneum | | >gi\|189313712\|ref\|NC_007416.2\|NC_007416 Tribolium castaneum linkage group 1=X, ref shotgun | | |
| Tribolium_castaneum | | >gi\|189313719\|ref\|NC_007424.2\|NC_007424 Tribolium castaneum linkage group 9, ref shotgun | | |
| Tribolium_castaneum | | >gi\|91192192\|ref\|NC_007419.1\|NC_007419 Tribolium castaneum linkage group 4, ref assembly shotgun | | |
| Tribolium_castaneum | | >gi\|189313716\|ref\|NC_007421.2\|NC_007421 Tribolium castaneum linkage group 6, ref | | |
| Tribolium_castaneum | | >gi\|189313717\|ref\|NC_007422.2\|NC_007422 Tribolium castaneum linkage group 7, ref | | |
| Trypanosoma_brucei | | >gi\|261325979\|emb\|FN554964.1\| Trypanosoma brucei gambiense DAL972 chromosome 1, complete sequence | | |
| Trypanosoma_cruzi | | >gi\|322830686\|gb\|ADWP01000001.1\| Trypanosoma cruzi strain Sylvio X10/1 sylviocontig_195, whole genome shotgun sequence | | |
| Xenopus_Silurana_tropicalis | | >gi\|58618664\|ref\|NC_006839.1\| Xenopus (Silurana) tropicalis mitochondrion, complete genome | | |
| Xenopus_Silurana_tropicalis | | str_ref_v4.2_chrUn.fa | | |
| Zea_mays | | >gi\|284930231\|gb\|CM000777.2\| Zea mays chromosome 1 | | |
| Zea_mays | | >gi\|284930230\|gb\|CM000778.2\| Zea mays chromosome 2 | | |
| Zea_mays | | >gi\|284930229\|gb\|CM000779.1\| Zea mays chromosome 3 | | |
| Zea_mays | | >gi\|284930228\|gb\|CM000780.1\| Zea mays chromosome 4 | | |
| Zea_mays | | >gi\|284930227\|gb\|CM000781.1\| Zea mays chromosome 5 | | |
| Zea_mays | | >gi\|284930226\|gb\|CM000782.1\| Zea mays chromosome 6 | | |
| Zea_mays | | >gi\|284930225\|gb\|CM000783.1\| Zea mays chromosome 7 | | |
| Zea_mays | | >gi\|284930224\|gb\|CM000784.1\| Zea mays chromosome 8 | | |
| Zea_mays | | >gi\|284930223\|gb\|CM000785.1\| Zea mays chromosome 9 | | |
| Zea_mays | | >gi\|284930222\|gb\|CM000786.1\| Zea mays chromosome 10 | | |
|  | |  | | |
|  | | | | |
