# Supplementary material for: Motif types, motif locations and base composition patterns around the RNA polyadenylation site in microorganisms, plants and animals
Source: BMC Evol Biol. 2014 Jul 23;14:162. doi: 10.1186/s12862-014-0162-7 (PMC4360255; doi:10.1186/s12862-014-0162-7)
Supplement: Additional file 2: — Predominance ranking of the upstream AAUAAA motif among all 4096 hexanucleotide motifs. [file s12862-014-0162-7-S2.docx]

Li and Du (2014) Motif types, motif locations and base composition patterns around the RNA polyadenylation site in microorganisms, plants and animals. BMC Evol. Biol.

**Additional file 2.** Predominance ranking of the upstream AAUAAA motif among all 4096 hexanucleotide motifs

| Species/group | AAUAAA (%)^1^ | Ranking |
| --- | --- | --- |
| *Apis mellifera* | 53.48 | 1st |
| *Bos taurus* | 63.61 | 1st |
| *Chlamydomonas reinhardtii* | 1.14 | 2 142nd |
| *Caenorhabditis elegans* | 50.64 | 1st |
| *Callithrix jacchus* | 55.7 | 1st |
| *Canis lupus* | 68.52 | 1st |
| *Ciona intestinalis* | 48.43 | 1st |
| *Danio rerio* | 61.73 | 1st |
| *Drosophila melanogaster* | 59.01 | 1st |
| *Equus caballus* | 46.39 | 1st |
| Fungi | 19.6 | 1st |
| *Gallus gallus* | 58.76 | 1st |
| *Homo sapiens* | 58.24 | 1st |
| *Medicago truncatula* | 11.76 | 16th |
| *Mus musculus* | 61.19 | 1st |
| *Oryctolagus cuniculus* | 59.82 | 1st |
| *Oryza sativa* | 15.3 | 1st |
| *Phytophthora infestans* | 11.1 | 4th |
| *Pongo abelii* | 58.52 | 1st |
| *Populus trichocarpa* | 11.16 | 4th |
| *Rattus norvegicus* | 55.96 | 1st |
| *Solanum lycopersicum* | 11.11 | 3rd |
| *Sorghum bicolor* | 11.16 | 1st |
| *Sus scrofa* | 40.52 | 1st |
| *Taeniopygia guttata* | 59.9 | 1st |
| *Trypanosoma cruzi* | 7.69 | 66th |
| Viruses | 32.79 | 1st |
| *Zea mays* | 9.03 | 1st |

^1^See Table 2 for the number of unique polyadenylation sites mapped. The exact percentage of the AAUAAA motif may vary depending on the mRNA datasets used.
