# Supplementary material for: Motif types, motif locations and base composition patterns around the RNA polyadenylation site in microorganisms, plants and animals
Source: BMC Evol Biol. 2014 Jul 23;14:162. doi: 10.1186/s12862-014-0162-7 (PMC4360255; doi:10.1186/s12862-014-0162-7)
Supplement: Additional file 3: — Top five most frequent pentanucleotides in each species in the 48-base upstream region. [file s12862-014-0162-7-S3.docx]

Li and Du (2014) Motif types, motif locations and base composition patterns around the RNA polyadenylation site in microorganisms, plants and animals. BMC Evol. Biol.

**Additional file 3.** Top five most frequent pentanucleotides in each species in the 48 base upstream region (Positions -3 to -50).

| Species | Total mRNA | | 1st (%) | | 2nd (%) | | 3rd (%) | | 4th (%) | | 5th (%) | |  |
| --- | --- | --- | --- | --- | --- | --- | --- | --- | --- | --- | --- | --- | --- |
| *Apis mellifera* | |  | | ATAAA | | AATAA | | AAATA | | AAAAT | | TAAAA | |
|  | | 187 | | 65.78 | | 61.5 | | 49.2 | | 45.45 | | 45.45 | |
| *Bos taurus* | |  | | ATAAA | | AATAA | | TAAAA | | AAATA | | AAAAT | |
|  | | 2679 | | 68.98 | | 66.74 | | 43.67 | | 41.96 | | 35.95 | |
| *Brachypodium distachyon* | | | | TTTTG | | TTTGT | | TTGTT | | TGTTT | | AATAA | |
|  | | 91 | | 28.57 | | 27.47 | | 26.37 | | 21.98 | | 20.88 | |
| *Caenorhabditis elegans* | |  | | ATAAA | | AATAA | | ATTTT | | TTTTT | | AAATT | |
|  | | 389 | | 71.47 | | 56.04 | | 46.79 | | 46.79 | | 44.73 | |
| *Callithrix jacchus* | |  | | AATAA | | ATAAA | | TAAAA | | AAATA | | AAAAT | |
|  | | 79 | | 65.82 | | 56.96 | | 44.3 | | 43.04 | | 36.71 | |
| *Canis lupus familiaris* | |  | | ATAAA | | AATAA | | AAATA | | TAAAA | | AAAAT | |
|  | | 108 | | 72.22 | | 70.37 | | 44.44 | | 36.11 | | 34.26 | |
| *Ciona intestinalis* | |  | | ATAAA | | AATAA | | AAATA | | TAAAA | | AAAAT | |
|  | | 287 | | 54.36 | | 53.66 | | 49.48 | | 37.63 | | 32.75 | |
| *Danio rerio* | |  | | ATAAA | | AATAA | | AAATA | | TAAAA | | TAAAT | |
|  | | 7246 | | 67.26 | | 64.9 | | 41.68 | | 40.73 | | 34.96 | |
| *Drosophila melanogaster* | | | | ATAAA | | AATAA | | AAATA | | TAAAT | | TAAAA | |
|  | | 954 | | 66.25 | | 64.26 | | 52.73 | | 42.66 | | 42.24 | |
| *Equus caballus* | |  | | ATAAA | | AATAA | | TAAAA | | AAAAT | | AAATA | |
|  | | 97 | | 50.52 | | 49.48 | | 37.11 | | 35.05 | | 34.02 | |
| *Gallus gallus* | |  | | ATAAA | | AATAA | | TAAAA | | AAATA | | AAAAT | |
|  | | 788 | | 64.72 | | 63.07 | | 44.29 | | 43.78 | | 35.15 | |
| *Homo sapiens* | |  | | ATAAA | | AATAA | | TAAAA | | AAATA | | AAAAT | |
|  | | 30499 | | 64.38 | | 61.6 | | 40.97 | | 40.6 | | 34.34 | |
| *Medicago truncatula* | |  | | ATTTT | | TATTT | | TTTTG | | TTTAT | | TTATT | |
|  | | 136 | | 38.24 | | 36.76 | | 35.29 | | 30.88 | | 30.88 | |
| *Mus musculus* | |  | | ATAAA | | AATAA | | TAAAA | | AAATA | | AAAAT | |
|  | | 8709 | | 67.07 | | 64.13 | | 40.87 | | 40.67 | | 33.38 | |
| *Oryctolagus cuniculus* | |  | | ATAAA | | AATAA | | AAATA | | TAAAA | | TAAAT | |
|  | | 224 | | 63.39 | | 62.95 | | 38.84 | | 36.61 | | 33.93 | |
| *Oryza sativa* | |  | | AATAA | | ATAAA | | TTGTT | | TGTTT | | TATAT | |
|  | | 693 | | 24.68 | | 20.35 | | 20.06 | | 18.61 | | 18.33 | |
| *Pongo abelii* | |  | | ATAAA | | AATAA | | TAAAA | | AAATA | | AAAAT | |
|  | | 1965 | | 63.66 | | 61.27 | | 40.66 | | 39.54 | | 33.84 | |
| *Populus trichocarpa* | |  | | ATTTT | | TATTT | | TTTAT | | TTTTT | | TTTTG | |
|  | | 1371 | | 30.71 | | 27.35 | | 25.89 | | 24.95 | | 24.73 | |
| *Protists Pf Tb* | |  | | AAAAT | | AATAA | | ATAAA | | TTTAT | | AAATA | |
|  | | 73 | | 36.99 | | 31.51 | | 31.51 | | 30.14 | | 28.77 | |
| *Rattus norvegicus* | |  | | ATAAA | | AATAA | | TAAAA | | AAATA | | AAAAT | |
|  | | 14263 | | 61.21 | | 58.86 | | 37.93 | | 36.43 | | 30.1 | |
| *Solanum lycopersicum* | |  | | TATTT | | ATTTT | | TTTGT | | TTTAT | | TTTTT | |
|  | | 450 | | 28.44 | | 27.33 | | 27.33 | | 27.11 | | 26 | |
| *Sorghum bicolor* | |  | | AATAA | | TGTTT | | TTTTG | | TTTGT | | ATAAA | |
|  | | 1685 | | 22.31 | | 20.36 | | 19.23 | | 19.11 | | 18.52 | |
| *Sus scrofa* | |  | | ATAAA | | AATAA | | TAAAA | | AAATA | | AAAAA | |
|  | | 8114 | | 44.75 | | 43.27 | | 34.58 | | 31.03 | | 30.07 | |
| *Taeniopygia guttata* | |  | | ATAAA | | AATAA | | TAAAA | | AAATA | | AAAAT | |
|  | | 808 | | 63.74 | | 61.88 | | 42.7 | | 42.7 | | 34.16 | |
| *Trypanosoma cruzi* | |  | | TTTTT | | TGTTT | | TTTGT | | GTTTT | | TTTTG | |
|  | | 52 | | 50 | | 46.15 | | 44.23 | | 42.31 | | 42.31 | |
| *Zea mays* | |  | | TGTTT | | TTTGT | | TTGTT | | AATAA | | ATTTT | |
|  | 10491 | | 19.77 | | 18.91 | | 18.49 | | 18.39 | | 18.32 | |  |
