# Supplementary material for: Motif types, motif locations and base composition patterns around the RNA polyadenylation site in microorganisms, plants and animals
Source: BMC Evol Biol. 2014 Jul 23;14:162. doi: 10.1186/s12862-014-0162-7 (PMC4360255; doi:10.1186/s12862-014-0162-7)
Supplement: Additional file 4: — Frequencies of known downstream motifs. [file s12862-014-0162-7-S4.docx]

Li and Du (2014) Motif types, motif locations and base composition patterns around the RNA polyadenylation site in microorganisms, plants and animals. BMC Evol. Biol.

**Additional file 5**. Frequencies of known motifs

| Species | UUAUUU (%) | CCUCCC (%) | UGUGUG (%) | GUGUGU (%) | UGUUUG (%) | AUGCGU (%) |
| --- | --- | --- | --- | --- | --- | --- |
| *Bos taurus* | 11.58 | 1.88 | 5.59 | 5.23 | 4.96 | 0.4 |
| *Danio rerio* | 15.94 | 0.24 | 8 | 6.77 | 8.6 | 0.61 |
| *Drosophila melanogaster* | 15.43 | 0.32 | 2.05 | 2.27 | 4.21 | 0.86 |
| *Homo sapiens* | 10.14 | 1.74 | 6.25 | 5.07 | 5.05 | 0.33 |
| *Mus musculus* | 9.65 | 1.86 | 8.35 | 6.64 | 5.82 | 0.25 |
| *Pongo abelii* | 10.52 | 1.78 | 6.03 | 4.92 | 4.92 | 0.62 |
| *Rattus norvegicus* | 7.73 | 1.42 | 6.84 | 5.96 | 5.08 | 0.23 |
| *Sus scrofa* | 6.38 | 1.64 | 4.32 | 3.73 | 3.32 | 0.21 |
| *Populus trichocarpa* | 9.65 | 1.09 | 2.39 | 2.09 | 5.57 | 0.9 |
| *Sorghum bicolor* | 6.15 | 0.41 | 5.05 | 3.04 | 7.68 | 0.62 |
| *Zea mays* | 6.45 | 0.64 | 4.29 | 3.34 | 7.28 | 1.12 |
| Mean | 9.97 | 1.18 | 5.38 | 4.46 | 5.68 | 0.56 |
